# Phylogenetic Analysis Reveals a Cryptic Species *Blastomyces gilchristii*, sp. nov. within the Human Pathogenic Fungus *Blastomyces dermatitidis*

**DOI:** 10.1371/journal.pone.0059237

**Published:** 2013-03-22

**Authors:** Elizabeth M. Brown, Lisa R. McTaggart, Sean X. Zhang, Donald E. Low, David A. Stevens, Susan E. Richardson

**Affiliations:** 1 Public Health Laboratories Toronto, Public Health Ontario, Toronto, Ontario, Canada; 2 Department of Microbiology, Mount Sinai Hospital, Toronto, Ontario, Canada; 3 Department of Laboratory Medicine and Pathobiology, University of Toronto, Toronto, Ontario, Canada; 4 Department of Pathology, Division of Medical Microbiology, The Johns Hopkins University School of Medicine, Baltimore, Maryland, United States of America; 5 Division of Infectious Diseases, Department of Medicine, Santa Clara Valley Medical Center, San Jose, California, United States of America; 6 Division of Microbiology, Department of Paediatric Laboratory Medicine, The Hospital for Sick Children, Toronto, Ontario, Canada; Duke University Medical Center, United States of America

## Abstract

**Background:**

Analysis of the population genetic structure of microbial species is of fundamental importance to many scientific disciplines because it can identify cryptic species, reveal reproductive mode, and elucidate processes that contribute to pathogen evolution. Here, we examined the population genetic structure and geographic differentiation of the sexual, dimorphic fungus *Blastomyces dermatitidis*, the causative agent of blastomycosis.

**Methodology/Principal Findings:**

Criteria for Genealogical Concordance Phylogenetic Species Recognition (GCPSR) applied to seven nuclear loci (*arf6, chs2*, *drk1, fads, pyrF, tub1,* and *its-2)* from 78 clinical and environmental isolates identified two previously unrecognized phylogenetic species. Four of seven single gene phylogenies examined (*chs2*, *drk1, pyrF,* and *its-2)* supported the separation of Phylogenetic Species 1 (PS1) and Phylogenetic Species 2 (PS2) which were also well differentiated in the concatenated *chs2-drk1-fads-pyrF-tub1-arf6-its2* genealogy with all isolates falling into one of two evolutionarily independent lineages. Phylogenetic species were genetically distinct with interspecific divergence 4-fold greater than intraspecific divergence and a high Fst value (0.772, *P*<0.001) indicative of restricted gene flow between PS1 and PS2. Whereas panmixia expected of a single freely recombining population was not observed, recombination was detected when PS1 and PS2 were assessed separately, suggesting reproductive isolation. Random mating among PS1 isolates, which were distributed across North America, was only detected after partitioning isolates into six geographic regions. The PS2 population, found predominantly in the hyper-endemic regions of northwestern Ontario, Wisconsin, and Minnesota, contained a substantial clonal component with random mating detected only among unique genotypes in the population.

**Conclusions/Significance:**

These analyses provide evidence for a genetically divergent clade within *Blastomyces dermatitidis*, which we use to describe a novel species, *Blastomyces gilchristii* sp. nov. In addition, we discuss the value of population genetic and phylogenetic analyses as a foundation for disease surveillance, understanding pathogen evolution, and discerning phenotypic differences between phylogenetic species.

## Introduction

Microorganisms are the most abundant life forms on earth in terms of protoplasmic biomass [1], [2] and second only to insects in estimated species diversity [3]. Yet, only an estimated 1–10% of all microbial species have been described [3]. Among fungi, only ∼74,000 species are known even though biodiversity species estimates range from 500,000 to 9.9 million [4]. The differentiation of fungal species has historically relied on morphologic, phenotypic, and reproductive characterization that satisfies a biological species concept [5], [6]. However, the advent of DNA sequencing and advancement of phylogenetic analysis has provided a powerful way to study the differentiation of fungal species at a more fundamental level by examining evolutionary processes such as population-level gene flow and genetic isolation that lead to the evolution of differential phenotypic characteristics. More recently, fungal species have been defined according to the phylogenetic species concept through multilocus sequence analysis of populations of individuals, rather than by tedious pair-wise mating tests or infrequent observation of reproductive structures that fulfill a biological species definition [7], [8]. Genealogical concordance phylogenetic species recognition (GCPSR) is a phylogenetic technique that defines species boundaries and examines reproductive mode. It is based on the principle that multiple gene phylogenies will be concordant (shared topology, due to fixation of formerly polymorphic loci following genetic isolation and drift) between species and will be discordant (conflicting topology, due to recombination and mutation) within a species [8], [9]. A key strength of this approach is that it does not require populations to be predefined, but instead can be used to identify populations and barriers to gene flow among populations *de novo* [9]. Applying GCPSR together with other measures of genetic recombination and gene flow, cryptic species and sexual recombination have been identified among the human pathogens *Histoplasma* [10], *Coccidioides* [9], [11]–[13], *Paracoccidioides* [14], [15], and *Absidia* [16], the filamentious fungi *Neurospora* [5], *Aspergillus* [17], [18], and *Penicillium* [19], and the mycorrhizal fungus *Cenococcum* [20]. Such analyses provide a strong genetic foundation for studies aiming to correlate pathogenic properties with species identity, historical biogeographic events with evolutionary origin [10], [21], and phylogeographic distribution with strain tracking and disease survillance [22]. Yet GCPSR studies have only begun to examine the many clinically significant fungal pathogens for cryptic biodiversity. Further development and application of these molecular phylogenetic methods to other genera are needed to describe novel fungal species and to understand their population structure and the genetic processes that contribute to their evolution.

In this study, we examined the population genetic structure and geographic differentiation of *Blastomyces dermatitidis* (teleomorph: *Ajellomyces dermatitidis*), the causative agent of blastomycosis, a rare but often-fatal systemic pyogranulomatous infection [23], [24]. *B. dermatitidis* is endemic to Africa, India, and to regions of North America bordering the Ohio and Mississippi river valleys, the Great Lakes, and the St. Lawrence River [23], [25]. Recently, there has been an increase in the number of blastomycosis cases reported in North America [26]–[30]. In Ontario, Canada, a statistically significant increase in the number of laboratory confirmed cases was observed between 1994 (5 cases) and 2002 (71 cases) [28]. It remains uncertain whether these recent increases represent the emergence of a more virulent pathogen population, exploitation of a novel host or niche by the pathogen, an environmentally driven increase in pathogen population size, or perhaps increased population exposure due to travel to endemic regions [28], [31], [32]. To date, little is known about the genetic structure and geographic differentiation of the species which would help elucidate the causes of these epidemiological changes. Although genotypic diversity among *B. dermatitidis* isolates has long been recognized [33], [34], only a single study using multilocus microsatellite analysis has assessed population genetic structure by delineating isolates from Wisconsin, U.S.A. into two genetically distinct groups [35]. However since the isolates were from a restricted locale, the global population genetic structure of *B. dermatitidis* remains unknown. Furthermore, genetic recombination between the two groups, a prerequisite for the recognition of cryptic species, was not assessed.

We applied population genetic and phylogenetic analyses to sequence data of multiple genetic loci from a worldwide collection of *B. dermatitidis* isolates to identify intra-specific populations and potentially cryptic phylogenetic species, to describe the extent of genetic recombination within and between species, and to investigate whether genetic populations display a phylogeographic distribution. Applying GCPSR criteria to sequences of seven nuclear loci we identified two previously unrecognized phylogenetic species, with reduced gene flow between taxa and recombination detected within but not between phylogenetic species. Our analyses provide evidence for the existence of two genetically distinct monophyletic clades within *B. dermatitidis*, which we use to describe a novel cryptic species, *Blastomyces gilchristii* sp. nov. Finally, we discuss the inherent value of applying these methods to various fungal genera to aid disease surveillance through strain-tracking, to understand pathogen evolution and disease epidemics, and to correlate differences in virulence or other phenotypic properties with phylogenetic species.

## Materials and Methods

### *B. dermatitidis* Isolates and Culture Growth Conditions

Seventy-eight geographically and temporally diverse *B. dermatitidis* strains isolated from human, canine, and environmental sources were studied (**Table S1**). Region of isolation represents the site of patient diagnosis, patient residence, or the site of environmental isolation. Strains were selected to represent all traditional endemic regions, in addition to diverse geographic localities within Ontario, Canada. For analysis purposes, the isolates were divided into six regional analysis groups to represent geographic concentrations of human cases of blastomycosis separated by ecological barriers or physical distance: (1) northwestern Ontario (21 isolates from Kenora, Ontario and the area within a 400 km radius); (2) central and southern Ontario (12 isolates); (3) Alberta and Saskatchewan (5 isolates); (4) Wisconsin, Minnesota (20 isolates); (5) areas of southeastern and central United States (South Carolina, Georgia, Louisiana, Mississippi, Kentucky, and Illinois (17 isolates)) and (6) southern Africa (South Africa, Rwanda, and Zimbabwe (3 isolates)). Ontario isolates were partitioned from those of Wisconsin and Minnesota to reflect the physical barrier of the Great Lakes separating these two geographic areas. Likewise, isolates from Ontario were split into two analysis groups to better reflect two distinct ecological zones: the Canadian shield of northwestern Ontario and the mixed wood plains of central and southern Ontario (http://www.mnr.gov.on.ca/en/Business/Biodiversity/2ColumnSubPage/STEL02_166891.html, accessed 2011 Nov 12).

Isolates were grown on potato dextrose agar (BD, Mississauga, ON) at 25°C for 3–7 days. Strains were then sub-cultured onto Formula D agar [36] and incubated at 37°C for 1–3 weeks until sufficient yeast growth was present for extraction of DNA.

### DNA Extraction, PCR Amplification, and Nucleotide Sequence Determination

Genomic DNA was extracted from yeast cells using the MasterPure yeast DNA purification kit (Epicentre Biotechnologies, Madison, WI), according to the manufacturers protocol with the following modifications. Approximately 10–15 acid-washed glass beads (Sigma-Aldrich, Oakville, ON) were added to each tube of lysis buffer inoculum. Samples were subsequently vortexed for 2 minutes using the Disruptor Genie (Scientific Industries Inc., Bohemia, New York) and incubated at 65°C for 45 minutes. Extracted DNA was suspended in 160 µl of TE buffer (Epicentre Biotechnologies).

Regions from seven nuclear genes were selected for multi locus sequence typing (MLST): (1) chitin synthase (*chs2*) [37], (2) histidine kinase (*drk1*) [38], (3) fatty acid desaturase (*fads*), (4) orotidine 5′-phosphate decarboxylase (*pyrF*), (5) alpha tubulin (*tub1*) [39], (6) ADP ribosylation factor 6 (*arf6*), and (7) internal transcribed spacer 2 (*its2)* [39]. Exons, introns, 5′ and 3′ untranslated regions were utilized to maximize nucleotide sequence variation among isolates.

PCR amplification of the target regions was performed using the Platinum Pfx DNA Polymerase kit (Life Technologies, Carlsbad, CA). Each PCR reaction contained 1.0 U of Platinum Pfx DNA Polymerase, 1× amplification buffer, 1000 µM MgSO_4_, 300 µM deoxynucleotide triphosphate mix (Life Technologies), 0.2 µM forward primer, 0.2 µM reverse primer (see **Table 1**), and 2 µl of genomic DNA in a 50 µl reaction. Reactions were denatured at 94°C for 2 minutes, followed by 35 cycles of 94°C for 30 seconds, 55–57°C for 30 seconds (see **Table 1**), and 68°C for 1 minute, followed by a final extension of 68°C for 5 minutes. PCR products were diluted 1∶20 in distilled deionized H_2_O in preparation for traditional Sanger sequencing.

**Table 1:** Summary of the MLST locus characteristics applied to 78 *B. dermatitidis* isolates.

Locus	Primer Sequence (5′ 3′)^a^	PCR annealingtemperature(°C)	Locussize(bp)	No. ofpolymorphicnucleotidesites	No. nucleotide sites				No. ofpolymorphicamino acidsites^b^
					FixedbetweenPS	SharedbetweenPS	Polymorphicwithin		
							PS1	PS2	
*arf6*	ATTTCCTTTCCCTGCGTGAG	56.5	250	3 (1.2%)	0	0	2	1	0
	CGGCCGGAGATATAGCATTA								
*drk1*	TCTGAATATGGCCTCGGAAG	56.5	646	13 (2.01%)	7	0	5	1	3
	TCGAATCCTCCCATAACAGG								
*tub1*	CTCTCCGCCCAATCTTCAT	55.5	650–655^c^	11 (1.69%)	0	1	10	0	1
	TGGCTCAAGATCGCAGTAGA								
*pyrF*	CAGACAGCTTCCCCAAAAACA	56.0	482	6 (1.24%)	1	1	4	0	3
	ACAAACCCCAGCACAAACCCT								
*chs2*	TGCTCCCTGCTTTCCTCCT	55.0	572	7 (1.22%)	3	0	2	2	6
	ACAACCCATACCGACACCAT								
*fads*	GCACGACCCTGATATCCAAC	57.0	637	3 (0.47%)	0	0	2	1	3
	TAGCCACATCCCCCAGTTTG								
*its2*	GCATCGATGAAGAACGCAGC	56.0	336	3 (0.89%)	1	0	1	1	0
	TCCTCCGCTTATTGATATGC								

a All primer sequences were designed for this study; with the exception of *its2* primers designed by [52].

b The *arf6* and *its-2* loci are located in untranslated regions of the gene and therefore do not encode amino acids.

c The *tub1* locus of contains a 5 bp insertion in 2 of the 78 isolates.

Bi-directional cycle sequencing reactions were performed using PCR primers and the BigDye ® Terminator v3.1 Cycle Sequence Kit (Life Technologies). Sequencing reactions contained 1 µl of BigDye ® Terminator v3.1, 1× of sequencing buffer, 0.5 µM of PCR primer, and 2 µl of diluted template DNA in a 20 µl reaction. Reactions were cycled according to the manufacturer’s instructions and the products were subsequently sequenced by capillary electrophoresis utilizing the 3730xl DNA Analyzer (Life Technologies).

### Sequence, Polymorphism, and Population Genetic Analysis

DNA sequences were trimmed, aligned, and primer sequences were removed using Bionumerics v.6.1 (Applied Maths, Sint-Martens-Latem, Belgium). Nucleotide polymorphisms were visually identified from sequence alignments of each gene region. The ratio of non-synonymous to synonymous amino acid changes (*d_N_/d_S_*) was calculated for each complete gene sequence to ensure that locus segments selected were under neutral selective pressure [40]. DnaSP v.5 was used to estimate the average pairwise divergence between isolates (pi) and the average pairwise divergence between populations (Dxy) [41]. The extent of gene flow between populations was inferred from the fixation index (Fst) calculated using Multilocus v.1.3b [42]. The signficance of the Fst value was assessed by comparing the original observed data set to the distribution of Fst values for 1000 randomized data sets in which alleles had been artificially re-sampled at each locus using Multilocus v.1.3.b [42].

### Phylogenetic Analysis

In Bionumerics v.6.1 (Applied Maths), majority consensus maximum parsimony trees were constructed for each gene region and the 3573 bp concatenated sequence of all nuclear sequences in the following order *chs2-drk1-fads-pyrF-tub1-arf6-its2*, with midpoint rooting applied to the concatenated gene phylogeny. Similarly, Bayesian analyses were performed using MrBayes v3.1 [43] for each gene region and the concatenated *chs2-drk1-fads-pyrF-tub1-arf6-its2* sequences. For each analysis, parameters and prior were modified to reflect the evolutionary model that best represented the data according to jModeltest [44] (JC for *arf6*, *fads*, and *tub1*, F81 for *chs2* and *pyrF*, and K80 for *drk1* and *its2*). The concatenated data set was partitioned to reflect each gene region with all parameters, priors and evolutionary rates estimated independently for each locus. Four Markov chains were run independently in two separate analyses for 1 000 000 generations, with samples taken every 100 generations. All analyses were performed twice to ensure that they were not trapped at local maxima. For each replicate, posterior probability values and the overall tree topology were compared to ensure analyses converged on a similar phylogeny.

To assess the robustness of clades identified by phylogenetic analyses, maximum parsimony bootstrap (MPB) values were calculated with 1000 randomizations in Bionumerics v.6.1 (Applied Maths) and Bayesian posterior probabilities (BPP) values were estimating using MrBayes v3.1 [43]. Consistency indices (CI) for majority consensus maximum parsimony trees were calculated using Bionumerics v6.1 (Applied Maths).

### Recombination and Linkage Disequilibrium Analysis

To assess mixis as an implicit measure of recombination, the degree of association among loci was quantified using the Index of Association (I_A_) [45], [46] and r_d_ values calculated with Multilocus v.1.3b [42]. Values were calculated for all isolates, for isolates within each clade identified in the phylogenetic analysis, and for isolates within each geographic regional analysis group. Based on these groupings, values were calculated for both the full data set and when the data set was reduced to unique genotypes (i.e. corrected for clonal genotypes) [46]. Complete panmixia expected in a fully recombining population will generate values near 0, while I_A_ and r_d_ values that are significantly greater than 0 signify deviation from panmixia, that is linkage disequilibrium [46]. To assess the significance of I_A_ and r_d_, the values for the observed data were compared to the distribution of I_A_ or r_d_ values calculated for 1000 randomized data sets that had been artificially recombined by re-sampling alleles at each locus without replacement with single nucleotide polymorphisms (SNP) at each locus shuffled together as a single linkage group [11]. The null hypothesis of panmixia was rejected if the I_A_ or r_d_ values of the observed data set were significantly larger than the distribution of I_A_ and r_d_ values for the randomized data set [11].

The Parsimony Tree Permutation Length Test (PTPLT) was performed to detect random mating in the phylogenetic data [11]. Phylogenetic trees were constructed based on multilocus genotypes in PAUP using parsimony, with loci treated as loci and alleles as character states [42]. Statistical significance was determined by comparing the actual length of the observed tree to the distribution of tree lengths of 1000 data sets that were artificially recombined as described above. Values were calculated for all isolates in the population, in addition to isolates within each clade identified in the phylogenetic analysis, and isolates within each geographic region. As above, this test was performed for both the full data set and when each dataset was reduced to unique genotypes [46].

To explicitly detect recombination in the sequence data, phylogenetic network analysis using split decomposition [47] was performed with SplitsTree v.4 [48], using the pairwise genetic distances of the concatenated *chs2-drk1-fads-pyrF-tub1-arf6-its2* sequences estimated in Bionumerics (Applied Maths). Bootstrapping values for the split decomposition network was calculated with 1000 replicates. The Phi test for recombination [49] was applied to the entire population and each phylogenetic species in SplitsTree v.4.

Finally, the simple four-gametes test was performed within each locus and between loci for each phylogenetic species using DnaSP v.5 [41]. Data were considered to have failed the simple four-gametes test if the minimum number of recombination events (R_M_) needed to explain the alleles was ≥1. [50].

### Mating Type Locus Determination

The mating type of each isolate was determined by amplifying the MAT loci as described by Meece et al. [35]. PCR products were analyzed by gel electrophoresis on a 1.5% agarose gel with 0.5 mg/L ethidium bromide. An isolate was considered positive for a specific mating type if a band was present for one locus and absent for the other mating type locus [35]. Mating type strains ATCC 18188 (+, containing the α box allele) and ATCC 18187 (-, containing the HMG box allele) available from the CBS-KNAW Fungal Biodiversity Centre (Utrecht, The Netherlands) and the American Type Culture Collection (Manassas, VA) were used to confirm the expected presence/absence of the a mating type locus. Mating type frequencies were assessed against the null hypothesis of a 1∶1 ratio using the chi square test with a significance level of *P*<0.05.

### Geographic Mapping

The distribution of isolates from Ontario and Wisconsin pertaining to each *B. dermatitidis* phylogenetic species was mapped using ArcGIS software (ESRI, Toronto, ON) and maps imported from the U.S. Geological Survey (http://data.geocomm.com/readme/usgs/atlas/html/statesmt.html, accessed 2011 Apr 20) and CanMap Route Logistics 2009v.4.

### Nomenclature

The electronic version of this article in Portable Document Format (PDF) will represent a published work according to the International Code of Nomenclature for algae, fungi, and plants, and hence the new names contained in the electronic version are effectively published under that Code from the electronic edition alone, so there is no longer any need to provide print copies.

In addition, new names contained in this work have been submitted to MycoBank from where they will be made available to the Global Names Index. The unique MycoBank number can be resolved and the associated information viewed through any standard web browser by appending the MycoBank number contained in this publication to the prefix http://www.mycobank.org/MycoTaxo.aspx?Link=T&Rec=MB803330. The online version of this work is archived and available from the following digital repositories: PubMed Central and LOCKSS.

## Results

### Polymorphism Analysis

Nucleotide sequences for the 7 loci analyzed in this study have been deposited in the Genbank database under the following accession numbers: *chs2* (JN561872–JN561949), *drk1* (JN561950–JN562027), *fads* (JN562028–JN562105), *pyrF* (JN562184–JN562261), *tub1* (JN562262–JN562339*), arf6* (JN561794–JN561871), and *its2* (JN562106–JN562183). Nucleotide sequence alignments are available in **Figure S1.**

A total of 46 nucleotide polymorphisms were identified in the 3573 bp of sequence analyzed, yielding an overall genetic diversity of 1.29%. The characteristics of each locus are summarized in **Table 1**. The number of polymorphic sites identified at each locus ranged from 3 in *fads, its2* and *arf6* to 13 in *drk1*. With the exception of the *tub1* locus, no insertions or deletions were detected in any of the sequenced fragments. A 5 bp insertion was detected in the *tub1* locus of 2 of the isolates. The insertion was viewed to have arisen from a single evolutionary event and counted as a single polymorphism. The ratio of nonsynonymous to synonymous amino acid substitutions (*d_N_/d_S_*) was below 1 for all genes, suggesting that all gene loci were under stabilizing selection [40]. Among the 78 isolates studied, 36 unique multilocus sequence types (ST) were identified. Of the 10 environmental isolates examined in our study, 7 were genetically identical to patient isolates and 3 had genetically unique sequence types.

### Identification of Phylogenetic Species within *B. dermatitidis*

**Figure 1:**
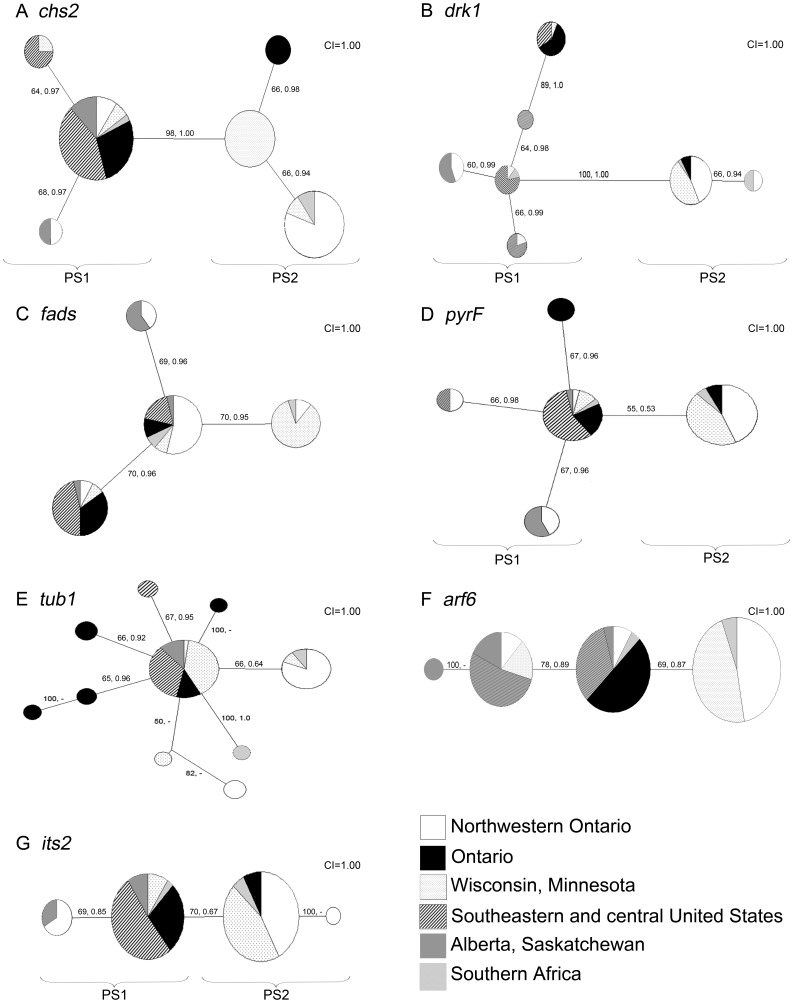
Genealogies of the seven MLST gene loci sequenced for 78 isolates of *B. dermatitidis*. Majority consensus maximum parsimony (MP) trees were constructed for each MLST locus region. (a) *chs2*, (b) *drk1*, (c) *fads*, (d) *pyrF*, (e) *tub1*, (f) *arf6*, and (g) *its2*. Isolates are coded by geographic region of isolation. For each group, the size of the patterned sector is proportional to the number of isolates from that geographic region. Values along branches represent maximum parsimony bootstrap values (MPB) and Bayesian posterior probability (BPP) values respectively. Branch values are provided where MP and Bayesian tree topology were in agreement.

**Figure 2:**
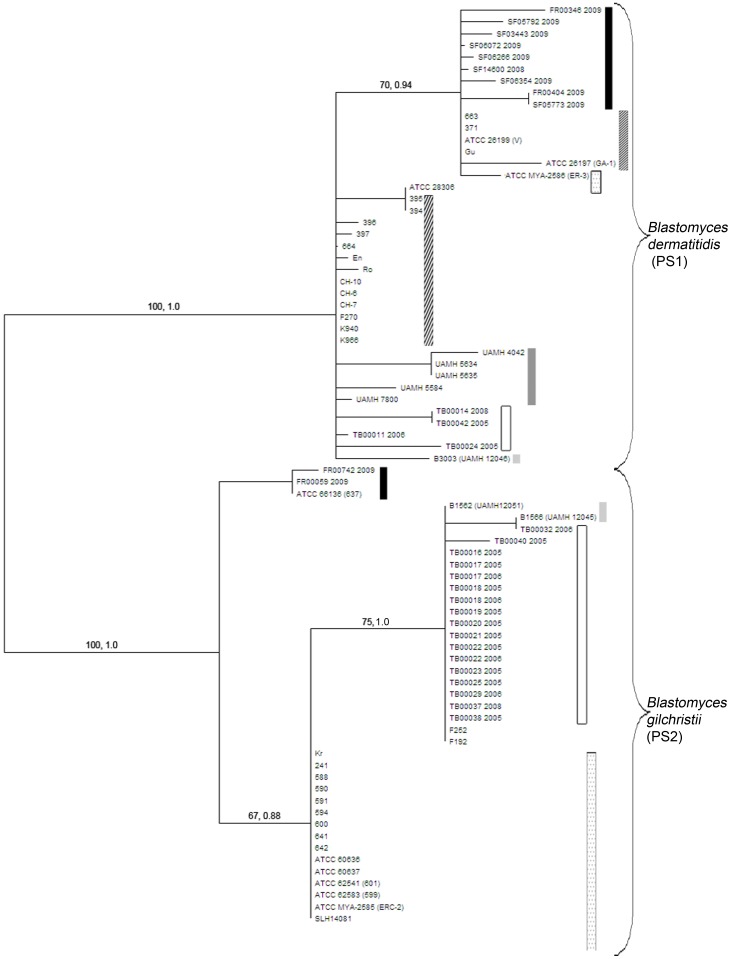
Maximum-parsimony tree constructed from the concatenated sequences of seven nuclear genes (*chs2-drk1-fads-pyrF-tub1-arf6-its2).* A majority consensus mid-point rooted maximum parsimony (MP) tree was constructed based on the concatenated sequence of seven MLST gene loci. The MP tree is displayed without logarithmic scaling so that genetic distance and geographic region of isolation could be viewed. Values along branches represent maximum parsimony bootstrap values (MPB) and Bayesian posterior probability (BPP) values respectively. Values for branches partitioning less than three isolates or those with MPB ≤70 and BPP≤0.95 are not shown. The tree is displayed with pattern coding for geographic regions as described in Figure 1. The MP tree displays a partition in the current species *B. dermatitidis* into two phylogenetic species: *B. dermatitidis* (PS1, clade 1) and *B. gilchristii* (PS2, clade 2).

Single gene phylogenies for each of the 7 loci were examined by constructing majority consensus maximum parsimony trees. Tree topologies derived from maximum parsimony and Bayesian analyses were very similar or identical for all loci with the exception of *tub1*, therefore only maximum parsimony trees for each locus are displayed with MPB and BPP values indicated on tree branches (**Figure 1**). The branching topology of the *tub1* maximum parsimony tree differed slightly from that predicted based on Bayesian analyses. For this locus, BPP values are displayed only on branches of the maximum parsimony tree that were also present in Bayesian analysis. **Figure 2** displays the *chs2-drk1-fads-pyrF-tub1-arf6-its2* concatenated maximum parsimony tree with MPB and BPP values displayed on the branches.

Two distinct monophyletic clades were identified by the phylogenetic analysis. To determine whether the clades represented independent evolutionary lineages and whether these lineages could be defined as phylogenetic species, we applied the criteria previously outlined by Dettman et al. [5] for phylogenetic species recognition by genealogical concordance and non-discordance (GCPSR). Briefly, a clade was recognized as an independent evolutionary lineage if it satisfied either of the following criteria: (1) Genealogical concordance: the clade was observed in the majority of single locus genealogies; or (2) Genealogical non-discordance: the clade was strongly supported by at least one genealogy with high MPB (≥70%) and BPP (≥0.95) values and was not contradicted by any other single locus genealogy with the same level of support [5]. Independent evolutionary lineages were defined as phylogenetic species if the concatenated *chs2-drk1-fads-pyrF-tub1-arf6-its2* genealogy revealed: (1) Genetic Differentiation: the phylogenetic species was distinct and well differentiated from other species; and (2) Exhaustive subdivision: all individuals in the population were placed within a phylogenetic species [5].

Four of the seven single-locus genealogies (*chs2*, *drk1, pyrF,* and *its-2)* separated clades 1 and 2 as independent evolutionary lineages, thereby satisfying criterion 1 of GCPSR (**Figure 1a, b, d, g**). The genealogies of *chs2* and *drk1* also strongly supported the separation of clades 1 and 2, with MPB ≥70% and BPP≥0.95 (**Figure 1a, b**). Therefore clades 1 and 2 represent independent monophyletic evolutionary lineages as recognized by genealogical concordance of single-locus genealogies [**5**].

To determine whether these two independent evolutionary lineages represent phylogenetic species, a majority consensus maximum parsimony tree for the concatenated sequence for all nuclear loci was examined (**Figure 2**). All isolates were partitioned into one of the two genetically distinct clades. The clades were well differentiated (MPB = 100%, BPP = 1.0 separating clade 1 from clade 2), with 12 fixed nucleotide differences separating clades 1 and 2. Based on the GCPSR criteria proposed by Dettman et al. [5], the currently defined species *B. dermatitidis* contains two phylogenetic species: Phylogenetic Species 1 (PS1) (clade 1) and Phylogenetic Species 2 (PS2) (clade 2) (**Figures 1** and **2**).

A substantial amount of internal phylogenetic structure was observed within PS1, which contained an internal clade. While the separation of this clade from PS1 was well supported by the *drk1* locus genealogy (**Figure 1b**, MPB = 89%, BPP = 1.0) and in the combined concatenated analyses (**Figure 2**, MPB = 70%, BPP = 1.0), this lineage was not supported as a monophyletic clade in any locus examined and therefore was not recognized as a phylogenetic species using the criteria applied by Dettman et al. [5].

The average pairwise divergence between phylogenetic species, 5.45×10^−3^ (σ = 5.40×10^−4^), was approximately 4-fold greater than the intraspecific pairwise divergence of either PS1 or PS2 (1.76×10^−3^ (σ = 1.20×10^−4^) and 0.69×10^−3^ (σ = 0.50×10^−4^) respectively).

To estimate gene flow between the phylogenetic species, Fst, which can range from 0 (no differentiation between populations due to unrestricted gene flow) to 1 (complete isolation due to the absence of gene flow) was calculated [20]. A statistically significant Fst value (0.77235, *P*<0.001) suggests restricted gene flow and genetic isolation between the two phylogenetic species.

### Assessment of Genetic Recombination and Linkage Disequilibrium

The extent of linkage disequilibrium and recombination between and within each phylogenetic species was assessed using (i) the Index of Association (I_A_) and r_d_ values; (ii) the Parsimony Tree Permutation Length Test (PTPLT); (iii) the Phi test and split decomposition analysis; and (iv) the four-gametes test and minimum number of recombination events (R_M_).

Panmixia as implicit evidence of recombination was assessed for all isolates, and separately within PS1 and PS2, by calculating I_A_ and r_d_ values both with and without correction for clone genotypes [46]. To account for recombination within but not between either phylogenetic species or distant geographic regions, the data were partitioned prior to randomization such that loci were shuffled within but not between the specified groups [11], [42] (**Table 2**). Panmixia was not detected among the 78 *B. dermatitidis* isolates when treated as a single population (*P*<0.001) or partitioned to mimic recombination within but not between either the two phylogenetic species (*P*<0.001) or the six geographic regions (*P*<0.001) (**Table 2**). Likewise, reducing the three datasets to unique genotypes also failed to detect panmixia (*P*<0.001) (**Table 2**). However, when the I_A_ and r_d_ values were calculated separately for each phylogenetic species, evidence for linkage disequilibrium disappeared for PS1 when the data were partitioned into six geographic regions, thereby implying recombination among isolates of the same geographic region (*P* = 0.121) (**Table 2** **)**. Panmixia was not detected among the 39 isolates of PS2 either with or without partitioning into 4 geographic regions (*P*<0.001). After reducing the dataset to 7 unique genotypes by removing clones [46] the I_A_ and r_d_ values (**Table 2**) declined but remained significant (*P* = 0.020) signifying a deviation from panmixia. These findings indicate that a considerable portion of the association between alleles was contained within the population structure separating PS1 from PS2.

**Table 2:** Linkage disequilibrium measurements for all isolates and unique genotypes in the entire population, the entire population partitioned into two phylogenetic species or six geographic regions, and the population within each phylogenetic species (*B. dermatitidis* (PS1) and *B. gilchristii* (PS2)).

		All Isolates			Unique Genotypes		
	Partition^a^	I_A_	r_d_	*P*-value^b^	I_A_	r_d_	*P*-value^b^
**All Isolates**	**None**	8.03	0.20	<0.001	5.14	0.12	<0.001
	**Phylogenetic Species (2)**	8.03	0.20	<0.001	5.14	0.12	<0.001
	**Geographic Regions (6)**	8.03	0.20	<0.001	6.06	0.14	<0.001
***B. dermatitidis*** ** (PS1)**	**None**	1.20	0.05	<0.001	1.04	0.04	<0.001
	**Geographic Regions (6)**	1.20	0.05	0.121	1.04	0.04	0.233
***B. gilchristii*** ** (PS2)**	**None**	1.67	0.26	<0.001	1.02	0.15	0.020
	**Geographic Regions (4)**	1.67	0.26	<0.001	n/a^c^	n/a	n/a

a Dataset partitioned into separate populations to mimic recombination within but not between either the two phylogenetic species or the 6 geographic regions.

b The statistical significance (*P* values) of each I_A_ or r_d_ value was assessed by comparing the I_A_ or r_d_ values calculated from the observed dataset to the distribution of I_A_ or r_d_ values for 1000 artificially re-sampled datasets.

c Due to the low number of unique genotypes in *B. gilchristii*, we were unable to partition the dataset by geographic region.

**Figure 3:**
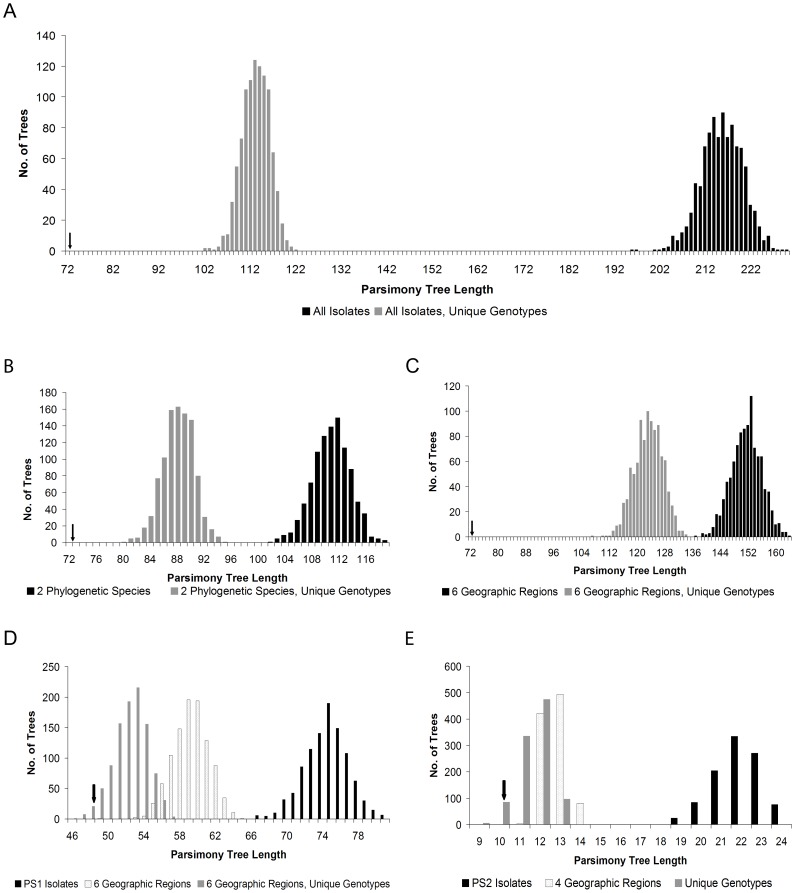
Parsimony Tree Permutation Length Test (PTPLT). A frequency distribution of tree lengths of maximum parsimony trees for 1000 artificially recombined datasets as compared to the observed tree length was calculated for: (a) All isolates in the *B. dermatitidis* population treated as a single population both with and without reduction to unique genotypes; (b) All isolates partitioned into two phylogenetic species (PS1) and (PS2) both with and without reduction to unique genotypes; (c) All isolates partitioned into 6 geographic regions both with and without reduction to unique genotypes; (d) Isolates of PS1 treated as a single population, partitioned into 6 geographic regions both with and without reduction to unique genotypes; (e) Isolates of PS2 treated as a single population, partitioned into 4 geographic regions, or reduced to unique genotypes (due to the low number of unique genotypes in PS2, we were unable to partition them by geographic region). Recombination was not detected between PS1 and PS2. Recombination was detected in PS1 (d) when partitioned by geographic region and reduced to unique genotypes and in PS2 (e) when reduced to unique genotypes.

The PTPLT was used to assess the extent of random mating among isolates by comparing the length of the observed parsimony tree to the lengths of trees constructed from 1000 artificial recombinations of the data **(** **Figure 3** **)**. In a clonal population, loci would be expected to have the same evolutionary topology and therefore the observed tree length should be significantly shorter than the distribution of tree lengths for the artificially recombined datasets [11]. Conversely, in a population undergoing random mating, different genealogies are expected to have varying topologies, and the observed tree length would be expected to be within the distribution of tree length randomizations [11]. The parsimony tree length of the observed data set was significantly shorter (*P*<0.001) than the distribution of tree lengths from 1000 permutations of the data randomly recombined across the entire population both with and without reduction of the dataset to unique genotypes (**Figure 3a**). Likewise, partitioning the data such that loci were randomized within but not between each of the two phylogenetic species or 6 geographic regions both with and without reduction to unique genotypes also generated distributions of tree lengths that were significantly shorter than the observed tree (*P*<0.001) (**Figure 3b, c**). PTPLT of all isolates of PS1 and PS2 analyzed separately also failed to detect random mating (*P*<0.001) (**Figure 3d, e**). However, partitioning by geographic region and reducing the dataset to 30 unique genotypes (i.e. clone correction [46]) rendered the observed tree length of PS1 isolates statistically undifferentiated from those of the artificially recombining population (**Figure 3d**) (*P* = 0.073). While PTPLT partitioned by geographic regions failed to detect random mating among the PS2 isolates, amalgamating the data from 39 isolates into 7 unique genotypes generated a distribution of tree lengths that were not statistically different from the observed tree (*P* = 0.092) (**Figure 3e**).

**Figure 4:**
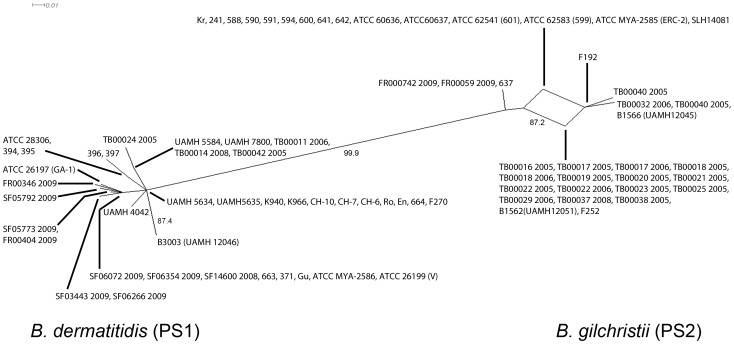
Phylogenetic network analysis **of the** ***B. dermatitidis.*** Split decomposition analysis was performed using the pairwise genetic distances of the concatenated *chs2-drk1-fads-pyrF-tub1-arf6-its2* sequences. Isolated split networks within *B. gilchristii* (PS2) suggests recombination within the *B. gilchristii* (PS2) but not between both populations. Bootstrapping values less than ≤0.75 are not shown.

The Phi test for recombination identified statistically significant evidence for recombination within PS1 (P<0.01). The Phi test did not identify statistically significant evidence for recombination within PS2 (P = 0.3069), however the split decomposition analysis revealed reticulate networks suggesting the possibility of recombination in PS2 independent of the PS1 population (**Figure 4**). Low genetic diversity, as was observed in PS2, is known to reduce the statistical power of the Phi test [51].

Finally, the simple four-gametes test was applied to explicitly assess recombination within PS1 and PS2. Although intralocus recombination was only observed within PS1, interlocus recombination between pairs of loci was observed in both PS1 and PS2, suggesting recombination is occurring within each phylogenetic species (**Table 3**).

**Table 3:** Intra- and interlocus simple four-gametes tests of each locus for *B. dermatitidis* (PS1) and *B. gilchristii* (PS2).

		*B. dermatitidis* (PS1)						
	Locus^a^	*chs2*	*drk1*	*fads*	*pyrF*	*tub1*	*arf6*	*its2*
***B. gilchristii*** ** (PS2)**	***chs2***	Pass:Pass^b^	**Fail**	**Fail**	**Fail**	**Fail**	**Fail**	Pass
	***drk1***	Pass	Pass:Pass	**Fail**	**Fail**	**Fail**	**Fail**	Pass
	***Fads***	**Fail**	Pass	Pass:Pass	**Fail**	**Fail**	**Fail**	**Fail**
	***pyrF***	**Fail**	Pass	**Fail**	**Fail:**Pass	**Fail**	**Fail**	**Fail**
	***tub1***	Pass	Pass	**Fail**	Pass	**Fail:**Pass	**Fail**	**Fail**
	***arf6***	Pass	Pass	Pass	**Fail**	Pass	Pass:Pass	**Fail**
	***its2***	Pass	Pass	Pass	Pass	Pass	Pass	Pass:Pass

a Results of the interlocus four-gametes test are presented as Pass (no recombination events detected) or Fail (>1 recombination events detected) along the upper right (B. dermatitidis) or lower left (B. gilchristii) of the table.

b The results along the diagonal represent the intralocus simple four-gametes test for B. dermatitidis:B. gilchristii.

In summary, the four-gametes test, PTPLT and split decomposition analysis detected random mating and recombination within PS2 when analyses were reduced to unique genotypes. Recombination, random mating, and linkage equilibrium was also detected within PS1 by the four-gametes test, the Phi test, the clone-corrected PTPLT and the I_A_ and r_d_ tests, but for the later two tests only when isolates were partitioned into 6 geographic regions, indicating that sexual reproduction among this group, which is broadly distributed across North America, may be limited by geographic distance. In contrast, none of these tests detected evidence of recombination in the PS1–PS2 combined dataset. Collectively, these analyses demonstrate that *B. dermatitidis* is not a single freely recombining species. Rather, recombination was only detected within each of the two phylogenetic species.

### Distribution of Mating Type Genes

Both mating types were found within each phylogenetic species (**Table S1**), however the distribution of mating types was significantly different from the expected 1∶1 ratio (PS1 *P* = 0.0112; PS2 *P = *0.0374), with each species containing more HMG box alleles. PS1 contained 25 HMG box positive isolates and 10 α box positive isolates, whereas PS2 contained 26 HMG box positive isolates and 13 α box positive isolates. Interestingly 8 of the 9 environmental isolates examined contained the HMG box group allele. Five of the PS2 ST’s in our study contained both mating type alleles.

### Phylogeography of *B. dermatitidis*

PS1 encompassed 39 isolates, from southeastern and central United States (17 isolates), southern and central Ontario (9 isolates), Alberta and Saskatchewan (5 isolates), Wisconsin and Minnesota (3 isolates), northwestern Ontario (4 isolates), and southern Africa (1 isolate). PS2 consisted of 39 isolates from northwestern Ontario (17 isolates), Wisconsin and Minnesota (17 isolates), 3 isolates from southern and central Ontario, and 2 additional isolates of southern African origin (**Figure 2**).

**Figure 5:**
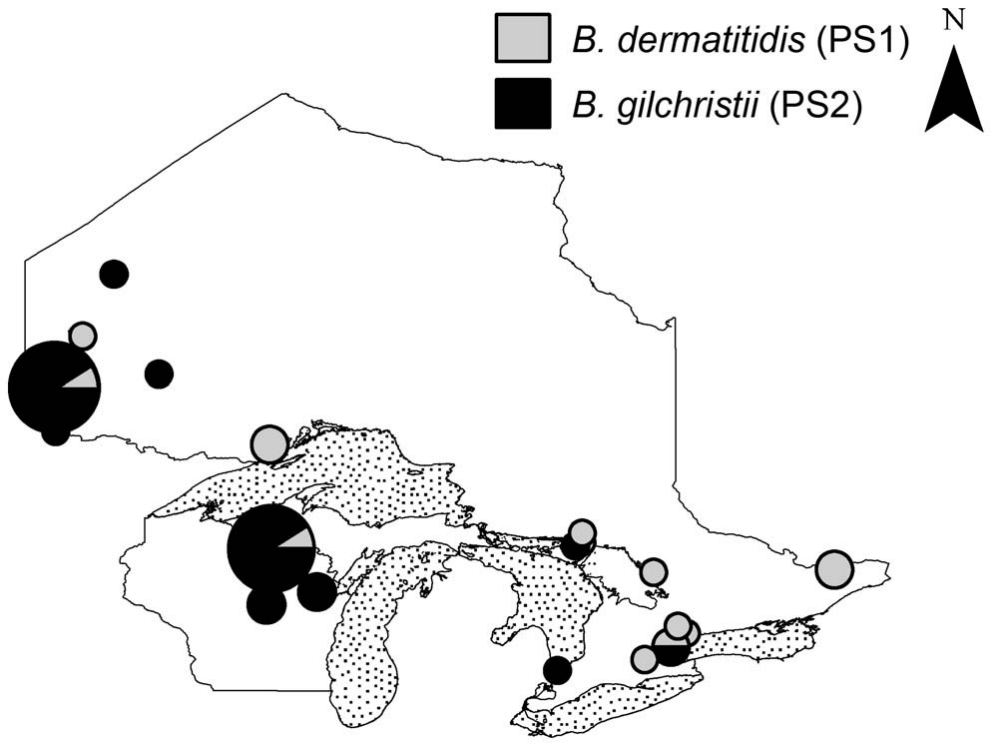
Geographic distribution of *B. dermatitidis* phylogenetic species across Ontario, Canada and Wisconsin, USA. Phylogenetic species are represented by circles with light grey (*B. dermatitidis*, PS1) and solid (*B. gilchristii*, PS2) fill corresponding to each phylogenetic species. Circle size is proportional to the number of isolates from each geographic site.

The geographic distribution of each phylogenetic species was geospatially mapped across Ontario and Wisconsin to illustrate species distribution across the most thoroughly sampled geographic region (**Figure 5**). PS2 was primarily restricted to northwestern Ontario, Wisconsin, and Minnesota (34 isolates) whereas isolates belonging to PS1 were found in these areas in addition to central and southern Ontario.

### Taxonomy

#### Blastomyces gilchristii

Brown E.M, McTaggart L.R., Zhang S.X., Low D.E., Stevens D.A., et Richardson S.E., sp. nov. [urn:lsid:mycobank.org:names:MB803330].

*Blastomyces gilchristii* can be diagnosed by the following nucleotide characters, which are fixed between *B. dermatitidis* and *B. gilchristii* respectively: chitin synthase at positions 422 (C:T), 461 (T:C) and 491 (G:A); histidine kinase at positions 139 (T:C), 235 (T:C), 469 (G:A), 544 (G:A), 586 (T:C), 595 (G:A), and 625 (C:T); orotidine 5′-phosphate decarboxylase at position 447 (T:C); and internal transcribed spacer 2 of rDNA at position 19 (T:C).

#### Diagnostic

Amplification of the internal transcribed spacer 2 of rDNA using the primers described by White et al. [52] can be used to distinguish *B. dermatitidis* (T) from *B. gilchristii* (C), which exhibit fixed nucleotide differences as noted at position 19.

#### Etymology

*Gilchristii*; after Thomas Casper Gilchrist who described the first case of blastomycosis in Baltimore, MD in 1894, and was the first to isolate the organism in 1898 with William Royal Stokes [53], [54].

#### Holotype

Public Health Ontario strain PHO TB00018/2005, isolated from a patient in Kenora, Ontario in 2005. Deposited in the CBS-KNAW Fungal Biodiversity Centre under accession number CBS 134223.

## Discussion

Using phylogenetic and population genetic analyses we identified two phylogenetic species within the pathogenic fungus *B. dermatitidis.* These phylogenetic species are genetically distinct with reduced gene flow between taxa and recombination detected within but not between phylogenetic species. Our analyses demonstrate the existence of two genetically isolated monophyletic clades within *B. dermatitidis*, which we have described as two species: *B. dermatitidis* (PS1) and *B. gilchristii* (PS2). Our study complements the small but growing number of reports demonstrating that GCPSR, together with other measures of genetic recombination and gene flow are capable of recognizing cryptic species and elucidating reproductive mode within a single morphologically defined fungal species [9], [10], [13]–[15], [17]–[19], [55]. Furthermore, fungal species recognized by this approach have been shown to correspond well with those recognized by mating tests indicative of biological species recognition [5], [6].

Four of the seven single-locus genealogies (*chs2*, *drk1, pyrF,* and *its-2*) examined provided strong evidence for the separation of *B. dermatitidis* (PS1) from *B. gilchristii* (PS2). These phylogenetic species were also well differentiated in the concatenated *chs2-drk1-fads-pyrF-tub1-arf6-its2* genealogy, with all genetic variants falling into one of the two lineages. The divergence between phylogenetic species is similar in magnitude to that found in *Coccidioides* spp. (*C. immitis* and *C. posadasii*) and *Paracoccidioides* spp. (S1, PS2, and PS3) [9], [14]. The high degree of genetic divergence separating *B. dermatitidis* (PS1) and *B. gilchristii* (PS2) (4-fold greater than intraspecific distances) and a high Fst value together suggest that these groups are well differentiated. Complementing the data on genetic diversity and gene flow, all the measures of recombination rejected the hypotheses of panmixia and random mating suggesting that *B. dermatitidis* is not a single freely recombining species. However, recombination was detected when each of the phylogenetic species was assessed separately. The four-gametes test and split decomposition analysis identified recombination within *B. gilchristii* (PS2) while random mating was detected by PTPLT when data were reduced to unique genotypes. Furthermore, the detection of *B. gilchristii* (PS2) isolates that are genetically identical by MLST but differ at the mating type loci suggests sexual reproduction on the smallest genetic scale, as was also observed in *Cryptococcus gattii* and *Penicillium marneffei* [56]–[58]. Recombination was also detected within *B. dermatitidis* (PS1) by the four-gametes test and the Phi test. Interestingly, random mating and linkage equilibrium in PS1 were only detected by the clone-corrected PTPLT and the I_A_ and r_d_ tests when isolates were partitioned into 6 geographic regions, indicating that sexual reproduction among this group, which is broadly distributed across North America, may be limited by geographic distance. Collectively, these results strongly support the separation of *B. dermatitidis* into two independent non-recombining phylogenetic species.

Across their North American range *B. dermatitidis* (PS1) isolates were obtained from all geographic regions sampled, whereas *B. gilchristii* (PS2) isolates were found predominately in northwestern Ontario, Wisconsin, and Minnesota. Of notable exception were three *B. gilchristii* isolates from southern Ontario. Additional studies are required to determine whether these cases are due to patient travel between regions or if they signal a larger geographic distribution of *B. gilchristii*. It is particularly noteworthy that the same locale (Eagle River, Wisconsin) can have environmental representatives of both species, underlining the need to study environmental isolates when studying human or animal outbreaks [33], [34]. The geographic overlap in phylogenetic species distribution observed in northwestern Ontario, Wisconsin, and Minnesota, is suggestive of a sympatric speciation event. A similar sympatric geographic distribution of cryptic species was also observed among the “Pb01-like” and S1 phylogenetic species of *Paracoccidioides* in central-western Brazil [14], [15] and among *C. immitis* and *C. posadasii* in southern California and Mexico [21].

It has been proposed that the natural reservoir for *B. dermatitidis* may be geographically restricted to a specific microenvironment [59]–[61]. It is possible that *B. gilchristii* (PS2) has adapted to a specific ecologic microniche in the hyper-endemic “disease hotspot” regions of northwestern Ontario, Wisconsin, and Minnesota, whereas *B. dermatitidis* (PS1) can inhabit a wider range of ecologic conditions with its distribution scattered throughout North America. This hypothesis fits well with described virulence and phenotypic characteristics of *B. dermatitidis*. Isolate SLH-14081, identified by our analysis as *B. gilchristii* (PS2), is highly virulent, causing infection in both immunocompetent humans and mice (http://www.broadinstitute.org/, accessed 2012 Dec 20). In contrast, *B. dermatitidis* (PS1) isolates ATCC MYA-2586 (ER-3) and ATCC 26197 (GA-1) have been characterized as avirulent or less virulent respectively, due to their impaired ability to cause illness or death in mice [61]–[63]. ATCC MYA-2586 (ER-3) sporulates well, which, if characteristic of *B. dermatitidis* (PS1) isolates may help explain its widespread geographic distribution (http://www.broadinstitute.org/, accessed 2012 Dec 20). Differences in growth on various media have also been documented for select *B. dermatitidis* (PS1) (ATCC MYA-2586 (ER-3)) and *B. gilchristii* (PS2) (ATCC MYA- 2585 (ERC-2)) isolates at 20°C and 37°C [61]. Extensive genotype-phenotype association studies involving many isolates of each species will be needed to assess whether differences in virulence, phenotype (e.g. morphology, growth rate, etc.), drug resistance, clinical manifestation, and prevalence exist between phylogenetic species and whether these characteristics may affect the ability of each phylogenetic species to survive in a specific environment. Standardized passage and storage history will be required to examine these correlations, as attenuated virulence among *Blastomyces* isolates has been documented [64]. Furthermore, these studies may succeed at revealing a genetic basis for variation in clinical symptoms and disease outcome observed for blastomycosis [24], [65], [66].

The majority of *B. gilchristii* (PS2) isolates in this study were obtained from regions considered hyper-endemic for blastomycosis namely the Kenora area of northwestern Ontario and the Eagle River region of Vilas County, Wisconsin [28], [33], [67]–[69]. In these areas, incidence rates exceed 100 cases per 100 000 with documented outbreaks [28], [60], [68], [70], [71]. Genetic analysis of *B. gilchristii* (PS2) isolates demonstrated low levels of genetic diversity with only 7 unique sequence types encompassing 8 unique polymorphisms in 39 isolates. Further, recombination analysis showed an “epidemic” population structure [46] with recombination detected only after accounting for the substantial clonal component of the population. This reduced diversity may reflect a recent speciation event, population bottleneck, or selective sweep for a preferred genotypic variant in the *B. gilchristii* (PS2) population [46], [55]. It is tempting to speculate that the high rates of blastomycosis in northwestern Ontario and Wisconsin are due to the recent emergence of *B. gilchristii* (PS2) as a more virulent or pathogenic species thereby generating a large number of clinical cases represented by only a few genotypes. Alternatively, exploitation of a novel niche by the pathogen and/or favourable environmental conditions may have caused an increase in *B. gilchristii* (PS2) population size [32]. Since sexual reproduction occurs only after mycelium development, local adaptation to a novel environment may affect the organism’s fitness and ability to mate thereby acting to sufficiently reduce gene flow between the species in sympatry [57], [72]. Adaptation to a new local habitat may have resulted in genetic incompatibility between species and/or geographic populations thereby facilitating a sympatric speciation event resulting in *B. dermatitidis* (PS1) and *B. gilchristii* (PS2) in spite of the observed overlapping geographic distributions [57], [72]. This hypothesis may explain why both phylogenetic species retain both mating type loci, where local adaptation and/or physical distance act to limit sexual reproduction to genetically and spatially similar individuals [57], [72]. Similar explanations have been proposed for spatially and genetically limited recombining populations of *P. marneffei* [57]. Furthermore resource competition can lead to habitat specialization particularly when linked to mating success as has been observed in *Penicillium chrysogenum* and related *Penicillium* spp. [19]. Virulence assays, resource competition studies, ecologic assessments of climatic and ecological niche factors, as well as examination of an epidemiologically well pedigreed group of isolates using additional and more variable genetic markers will be required to differentiate among these possibilities. Future studies will need to include patient isolates with detailed travel and residence history, since for the majority of strains examined the region of isolation represents the site of patient diagnosis, which may not be the site of acquisition. Additional isolates from regions underrepresented in our analyses (i.e. Africa and India) also need to be examined.

Interestingly, the phylogenetic species identified in our analyses correlate well with the genetic groups identified in previous studies. Thirty-four of the isolates included in our study had previously been characterized by PCR RFLP [33]. All PCR RFLP genotypic group A isolates examined in our study were identified as *B. gilchristii* (PS2). Furthermore, like *B. gilchristii* (PS2), genotypic group A isolates showed an analogous geographic distribution and lacked genetic variation compared with other genotypes [33]. Interestingly, two separate outbreak investigations in Wisconsin have identified genotypic group A as the predominant organism isolated from patients [33], [71]. Similarly, *B. gilchristii* (PS2) was responsible for the majority of Wisconsin and northwestern Ontario cases in our analysis. The *B. dermatitidis* (PS1) and *B. gilchristii* (PS2) designations of the isolates examined in this study do not correlate with PCR RFLP genotypic groups B and C, falling into either category [33].

Nine of the isolates included in our study were also examined previously by multilocus microsatellite analysis; however, the authors did not publish a multilocus microsatellite genetic group for four of these nine isolates [35]. Therefore we were only able to examine concordance of MLST and multilocus microsatellite analyses for five isolates, for which a multilocus microsatellite group designation was provided [35]. Three environmental isolates from Georgia (395, 396, and 397) were identified as *B. dermatitidis* (PS1) by MLST and Group 2 by multilocus microsatellite analysis, suggesting *B. dermatitidis* may represent multilocus microsatellite Group 2 [35]. Two isolates, ATCC 60636 and ATCC 60637, were designated *B. gilchristii* (PS2) by MLST and Group 1 by multilocus microsatellite analysis, and since both analyses report low allelic diversity in these genetic groups, it is possible that *B. gilchristii* (PS2) described here is equivalent to Group 1 identified by multilocus microsatellite analysis [35]. Studies using *Coccidioides* have shown that phylogenies inferred using genes and microsatellites were identical [13], [73]. Microsatellite analysis may be a less cumbersome and less expensive method of phylogenetic reconstruction compared to sequencing multiple genetic loci; however, microsatellite size limits may constrain the genetic distance that can accrue between taxa thus underestimating phylogenetic distance [13], [21]. Furthermore the high mutation rate may produce homoplasy confounding the phylogeny [13], [21]. Therefore, GCPSR using sequence data from multiple genetic loci is an essential and foundational step when describing cryptic species using phylogenetic analysis [13].

Earlier studies noted a number of differences between African (serotype 2) and North American (serotype 1) strains of *B. dermatitidis,* including differences in morphology, clinical manifestation, ease of mycelial-to-yeast phase conversion, and serotype [74]–[77]. However, 26S rRNA sequence data and DNA melting curve analysis demonstrated that the African serotype 2 strains are in fact more closely related to *Emmonsia* spp. than to the North American type (serotype 1) of *B. dermatitidis* [78]. The African strains examined here included both *B. dermatitidis* (PS1) and *B. gilchristii* (PS2) isolates and were previously characterized as serogroup 1 along with two North American strains ATCC 26199 (V) and ATCC 60636 [76]. Clearly, *B. dermatitidis* (PS1) and *B. gilchristii* (PS2) represent phylogenetic species within serotype 1 identified as *B. dermatitidis*.

The widespread application of the phylogenetic and population genetic approaches applied in this study represents an appealing strategy for unraveling the putative evolutionary differences between microbial species. Utilizing these methods, we can accurately assess variables affecting the microorganism’s evolution in a manner that is consistent with what is known of its reproductive behaviour, population structure, geographic distribution, and species boundaries. For instance, in an “epidemic” (outbreak), a temporary association among loci (i.e. clonality) would be observed in an organism that is effectively sexually recombining over the long term [46]. Alternately, a population containing more than one genetically isolated taxon may be mistakenly identified as non-recombining, if the species is not analyzed for barriers to gene flow and recombination [46], [79]. Using this knowledge can also help to determine which analysis methods will be most revealing for reliably investigating the relationships among isolates, since the level of recombination depends on both the pathogen under study and the population sampled. This knowledge is particularly useful in the context of molecular disease surveillance. Analyses such as Bayesian Assignment Tests and eBURST analysis have recently been applied to fungal pathogens with recombining and clonal reproductive structures respectively, to infer population structure and assign individuals of unknown origin to their likely source population [22]. We anticipate these approaches will be increasingly applied in public health settings for evaluating trends in disease characteristics and occurrence. Beyond identifying cryptic species, the methods applied here promise to define the population genetic structure and geographic differentiation of species and populations thereby providing a genetic foundation for investigating pathogen evolution and emergence, developing informative strain-tracking tools for disease surveillance, and associating phenotypic differences with phylogenetic species.

## Supporting Information

Figure S1 Sequence Alignments. Sequence alignments for the seven MLST genes sequences studied (*chs2, drk1, fads, pyrF, tub1, arf6*, and *its2)*. Untranslated regions (UTR), Exon and Introns are marked. Nucleotide polymorphisms are highlight in grey.
(PDF)

Table S1 Characteristics of *B. dermatitidis* isolates studied.
(PDF)
