# Correction: Phylogenetic Analysis Reveals a Cryptic Species *Blastomyces gilchristii*, sp. nov. within the Human Pathogenic Fungus *Blastomyces dermatitidis*

**DOI:** 10.1371/journal.pone.0168018

**Published:** 2016-12-09

**Authors:** Elizabeth M. Brown, Lisa R. McTaggart, Sean X. Zhang, Donald E. Low, David A. Stevens, Susan E. Richardson

In S1 Table, Fig 2 and Fig 4, the Isolate Identification Numbers for three of the strains are incorrect. CDC B1566 (UAMH 12045) should be CDC B1466 (UAMH 10245). B3003 (UAMH 12046) should be CDC B3003 (UAMH 10246). CDC B1562 (UAMH 12051) should be CDC B1562 (UAMH 10251). Please see the corrected S1 Table, Fig 2 and Fig 4 here.

**Fig 2:**
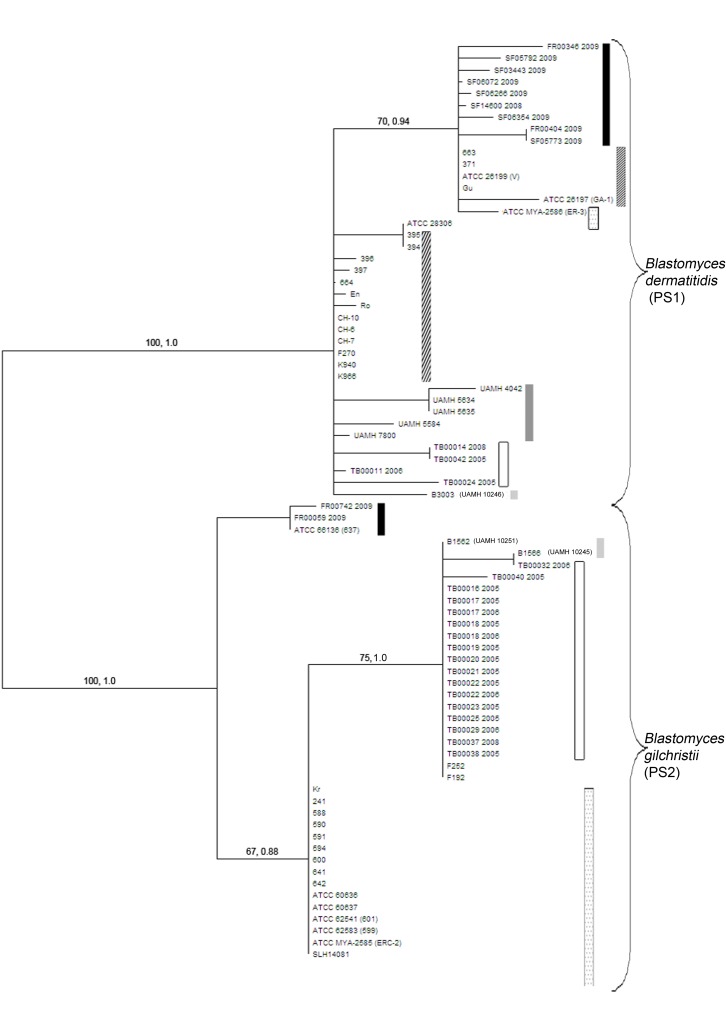
Maximum-parsimony tree constructed from the concatenated sequences of seven nuclear genes (*chs2-drk1-fads-pyrF-tub1-arf6-its2*). A majority consensus mid-point rooted maximum parsimony (MP) tree was constructed based on the concatenated sequence of seven MLST gene loci. The MP tree is displayed without logarithmic scaling so that genetic distance and geographic region of isolation could be viewed. Values along branches represent maximum parsimony bootstrap values (MPB) and Bayesian posterior probability (BPP) values respectively. Values for branches partitioning less than three isolates or those with MPB ≤70 and BPP≤0.95 are not shown. The tree is displayed with pattern coding for geographic regions as described in Fig 1. The MP tree displays a partition in the current species *B*. *dermatitidis* into two phylogenetic species: *B*. *dermatitidis* (PS1, clade 1) and *B*. *gilchristii* (PS2, clade 2).

**Fig 4:**
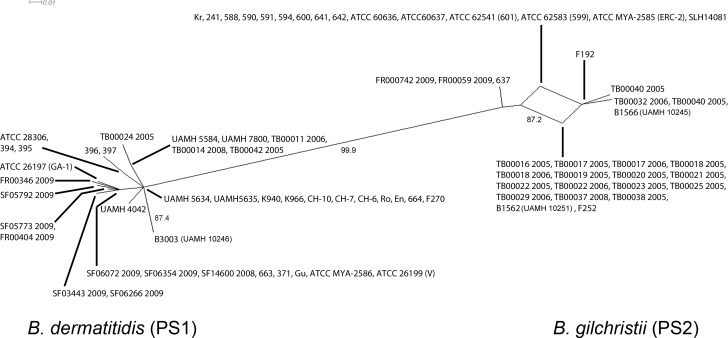
Phylogenetic network analysis of the *B*. *dermatitidis*. Split decomposition analysis was performed using the pairwise genetic distances of the concatenated *chs2-drk1-fads-pyrF-tub1-arf6-its2* sequences. Isolated split networks within *B*. *gilchristii* (PS2) suggests recombination within the *B*. *gilchristii* (PS2) but not between both populations. Bootstrapping values less than ≤0.75 are not shown.

## Supporting Information

S1 Table Characteristics of *B*. *dermatitidis* isolates studied. (DOCX)
