# Supplementary material for: Phylogenetic Analysis Reveals a Cryptic Species Blastomyces gilchristii, sp. nov. within the Human Pathogenic Fungus Blastomyces dermatitidis
Source: PLoS One. 2013 Mar 22;8(3):e59237. doi: 10.1371/journal.pone.0059237 (PMC3606480; doi:10.1371/journal.pone.0059237)
Supplement: Figure S1 — Sequence Alignments. Sequence alignments for the seven MLST genes sequences studied (chs2, drk1, fads, pyrF, tub1, arf6, and its2). Untranslated regions (UTR), Exon and Introns are marked. Nucleotide polymorphisms are highlight in grey. (PDF) [file pone.0059237.s001.pdf]

Figure S1: Nucleotide Sequence Alignments

arf6

UTR

396	TGCTGTGTTGTCCCTCAATCTCGGCTGAGCTGCGCCAGCCGAATGGTTGGTCGAGTGGAATTACTAACTTGTGTTTCAGCGTGTTTCTTATTCATTTACCTCGCAGGGCCTGACACTCCCTTAGTTTTCAATTATCGAACACCCATTTTTCTCCTCATCTGCTCCACCATATCAGGGCCTAGAAATAGATATCAACCGATTGGACTTTGCTTTATTGCCTGACACGCCATTCTTACGCTGCGTACTCCTC
ATCC 66136 (637)	TGCTGTGTTGTCCCTCAATCTCGGCTGAGCTGCGCCAGCCGAATGGTTGGTCGAGTGGAATTACTAACTTGTGTTTCAGCGTGTTTCTTATTCATTTACCTCGCAGGGCCTGACACTCCCTTAGTTTTCAATTATCGAACACCCATTTTTCTCCTCATCTGCTCCACCATATCAGGGCCTAGAAATAGATATCAACCGATTGGACTTTGCTTTATTGCCTGACACGCCATTCTTACGCTGCGTACTCCTC
B3003 (UAMH 12046)	TGCTGTGTTGTCCCTCAATCTCGGCTGAGCTGCGCCAGCCGAATGGTTGGTCGAGTGGAATTACTAACTTGTGTTTCAGCGTGTTTCTTATTCATTTACCTCGCAGGGCCTGACACTCCCTTAGTTTTCAATTATCGAACACCCATTTTTCTCCTCATCTGCTCCACCATATCAGGGCCTAGAAATAGATATCAACCGATTGGACTTTGCTTTATTGCCTGACACGCCATTCTTACGCTGCGTACTCCTC
CH-10	TGCTGTGTTGTCCCTCAATCTCGGCTGAGCTGCGCCAGCCGAATGGTTGGTCGAGTGGAATTACTAACTTGTGTTTCAGCGTGTTTCTTATTCATTTACCTCGCAGGGCCTGACACTCCCTTAGTTTTCAATTATCGAACACCCATTTTTCTCCTCATCTGCTCCACCATATCAGGGCCTAGAAATAGATATCAACCGATTGGACTTTGCTTTATTGCCTGACACGCCATTCTTACGCTGCGTACTCCTC
En	TGCTGTGTTGTCCCTCAATCTCGGCTGAGCTGCGCCAGCCGAATGGTTGGTCGAGTGGAATTACTAACTTGTGTTTCAGCGTGTTTCTTATTCATTTACCTCGCAGGGCCTGACACTCCCTTAGTTTTCAATTATCGAACACCCATTTTTCTCCTCATCTGCTCCACCATATCAGGGCCTAGAAATAGATATCAACCGATTGGACTTTGCTTTATTGCCTGACACGCCATTCTTACGCTGCGTACTCCTC
FR00346 2009	TGCTGTGTTGTCCCTCAATCTCGGCTGAGCTGCGCCAGCCGAATGGTTGGTCGAGTGGAATTACTAACTTGTGTTTCAGCGTGTTTCTTATTCATTTACCTCGCAGGGCCTGACACTCCCTTAGTTTTCAATTATCGAACACCCATTTTTCTCCTCATCTGCTCCACCATATCAGGGCCTAGAAATAGATATCAACCGATTGGACTTTGCTTTATTGCCTGACACGCCATTCTTACGCTGCGTACTCCTC
FR00742 2009	TGCTGTGTTGTCCCTCAATCTCGGCTGAGCTGCGCCAGCCGAATGGTTGGTCGAGTGGAATTACTAACTTGTGTTTCAGCGTGTTTCTTATTCATTTACCTCGCAGGGCCTGACACTCCCTTAGTTTTCAATTATCGAACACCCATTTTTCTCCTCATCTGCTCCACCATATCAGGGCCTAGAAATAGATATCAACCGATTGGACTTTGCTTTATTGCCTGACACGCCATTCTTACGCTGCGTACTCCTC
K940	TGCTGTGTTGTCCCTCAATCTCGGCTGAGCTGCGCCAGCCGAATGGTTGGTCGAGTGGAATTACTAACTTGTGTTTCAGCGTGTTTCTTATTCATTTACCTCGCAGGGCCTGACACTCCCTTAGTTTTCAATTATCGAACACCCATTTTTCTCCTCATCTGCTCCACCATATCAGGGCCTAGAAATAGATATCAACCGATTGGACTTTGCTTTATTGCCTGACACGCCATTCTTACGCTGCGTACTCCTC
Ro	TGCTGTGTTGTCCCTCAATCTCGGCTGAGCTGCGCCAGCCGAATGGTTGGTCGAGTGGAATTACTAACTTGTGTTTCAGCGTGTTTCTTATTCATTTACCTCGCAGGGCCTGACACTCCCTTAGTTTTCAATTATCGAACACCCATTTTTCTCCTCATCTGCTCCACCATATCAGGGCCTAGAAATAGATATCAACCGATTGGACTTTGCTTTATTGCCTGACACGCCATTCTTACGCTGCGTACTCCTC
SF03443 2009	TGCTGTGTTGTCCCTCAATCTCGGCTGAGCTGCGCCAGCCGAATGGTTGGTCGAGTGGAATTACTAACTTGTGTTTCAGCGTGTTTCTTATTCATTTACCTCGCAGGGCCTGACACTCCCTTAGTTTTCAATTATCGAACACCCATTTTTCTCCTCATCTGCTCCACCATATCAGGGCCTAGAAATAGATATCAACCGATTGGACTTTGCTTTATTGCCTGACACGCCATTCTTACGCTGCGTACTCCTC
SF05773 2009	TGCTGTGTTGTCCCTCAATCTCGGCTGAGCTGCGCCAGCCGAATGGTTGGTCGAGTGGAATTACTAACTTGTGTTTCAGCGTGTTTCTTATTCATTTACCTCGCAGGGCCTGACACTCCCTTAGTTTTCAATTATCGAACACCCATTTTTCTCCTCATCTGCTCCACCATATCAGGGCCTAGAAATAGATATCAACCGATTGGACTTTGCTTTATTGCCTGACACGCCATTCTTACGCTGCGTACTCCTC
SF05792 2009	TGCTGTGTTGTCCCTCAATCTCGGCTGAGCTGCGCCAGCCGAATGGTTGGTCGAGTGGAATTACTAACTTGTGTTTCAGCGTGTTTCTTATTCATTTACCTCGCAGGGCCTGACACTCCCTTAGTTTTCAATTATCGAACACCCATTTTTCTCCTCATCTGCTCCACCATATCAGGGCCTAGAAATAGATATCAACCGATTGGACTTTGCTTTATTGCCTGACACGCCATTCTTACGCTGCGTACTCCTC
SF06072 2009	TGCTGTGTTGTCCCTCAATCTCGGCTGAGCTGCGCCAGCCGAATGGTTGGTCGAGTGGAATTACTAACTTGTGTTTCAGCGTGTTTCTTATTCATTTACCTCGCAGGGCCTGACACTCCCTTAGTTTTCAATTATCGAACACCCATTTTTCTCCTCATCTGCTCCACCATATCAGGGCCTAGAAATAGATATCAACCGATTGGACTTTGCTTTATTGCCTGACACGCCATTCTTACGCTGCGTACTCCTC
SF06266 2009	TGCTGTGTTGTCCCTCAATCTCGGCTGAGCTGCGCCAGCCGAATGGTTGGTCGAGTGGAATTACTAACTTGTGTTTCAGCGTGTTTCTTATTCATTTACCTCGCAGGGCCTGACACTCCCTTAGTTTTCAATTATCGAACACCCATTTTTCTCCTCATCTGCTCCACCATATCAGGGCCTAGAAATAGATATCAACCGATTGGACTTTGCTTTATTGCCTGACACGCCATTCTTACGCTGCGTACTCCTC
SF06354 2009	TGCTGTGTTGTCCCTCAATCTCGGCTGAGCTGCGCCAGCCGAATGGTTGGTCGAGTGGAATTACTAACTTGTGTTTCAGCGTGTTTCTTATTCATTTACCTCGCAGGGCCTGACACTCCCTTAGTTTTCAATTATCGAACACCCATTTTTCTCCTCATCTGCTCCACCATATCAGGGCCTAGAAATAGATATCAACCGATTGGACTTTGCTTTATTGCCTGACACGCCATTCTTACGCTGCGTACTCCTC
SF14600 2008	TGCTGTGTTGTCCCTCAATCTCGGCTGAGCTGCGCCAGCCGAATGGTTGGTCGAGTGGAATTACTAACTTGTGTTTCAGCGTGTTTCTTATTCATTTACCTCGCAGGGCCTGACACTCCCTTAGTTTTCAATTATCGAACACCCATTTTTCTCCTCATCTGCTCCACCATATCAGGGCCTAGAAATAGATATCAACCGATTGGACTTTGCTTTATTGCCTGACACGCCATTCTTACGCTGCGTACTCCTC
TB00011 2006	TGCTGTGTTGTCCCTCAATCTCGGCTGAGCTGCGCCAGCCGAATGGTTGGTCGAGTGGAATTACTAACTTGTGTTTCAGCGTGTTTCTTATTCATTTACCTCGCAGGGCCTGACACTCCCTTAGTTTTCAATTATCGAACACCCATTTTTCTCCTCATCTGCTCCACCATATCAGGGCCTAGAAATAGATATCAACCGATTGGACTTTGCTTTATTGCCTGACACGCCATTCTTACGCTGCGTACTCCTC
TB00024 2005	TGCTGTGTTGTCCCTCAATCTCGGCTGAGCTGCGCCAGCCGAATGGTTGGTCGAGTGGAATTACTAACTTGTGTTTCAGCGTGTTTCTTATTCATTTACCTCGCAGGGCCTGACACTCCCTTAGTTTTCAATTATCGAACACCCATTTTTCTCCTCATCTGCTCCACCATATCAGGGCCTAGAAATAGATATCAACCGATTGGACTTTGCTTTATTGCCTGACACGCCATTCTTACGCTGCGTACTCCTC
UAMH 7800	TGCTGTGTTGTCCCTCAATCTCGGCTGAGCTGCGCCAGCCGAATGGTTGGTCGAGTGGAATTACTAACTTGTGTTTCAGCGTGTTTCTTATTCATTTACCTCGCAGGGCCTGACACTCCCTTAGTTTTCAATTATCGAACACCCATTTTTCTCCTCATCTGCTCCACCATATCAGGGCCTAGAAATAGATATCAACCGATTGGACTTTGCTTTATTGCCTGACACGCCATTCTTACGCTGCGTACTCCTC
371	TGCTGTGTTGTCCCTCAATCTCGGCTGAGCTGCGCCAGCCGAATGGTTGGTCGAGTGGAATTACTAACTTGTGTTTCAGCGTGTTTCTTATTCATTTACCTCGCAGGGCCTGACACTCCCTTAGTTTTCAATTATCGAACCCCCATTTTTCTCCTCATCTGCTCCACCATATCAGGGCCTAGAAATAGATATCAACCGATTGGACTTTGCTTTATTGCCTGACACGCCATTCTTACGCTGCGTACTCCTC
397	TGCTGTGTTGTCCCTCAATCTCGGCTGAGCTGCGCCAGCCGAATGGTTGGTCGAGTGGAATTACTAACTTGTGTTTCAGCGTGTTTCTTATTCATTTACCTCGCAGGGCCTGACACTCCCTTAGTTTTCAATTATCGAACCCCCATTTTTCTCCTCATCTGCTCCACCATATCAGGGCCTAGAAATAGATATCAACCGATTGGACTTTGCTTTATTGCCTGACACGCCATTCTTACGCTGCGTACTCCTC
663	TGCTGTGTTGTCCCTCAATCTCGGCTGAGCTGCGCCAGCCGAATGGTTGGTCGAGTGGAATTACTAACTTGTGTTTCAGCGTGTTTCTTATTCATTTACCTCGCAGGGCCTGACACTCCCTTAGTTTTCAATTATCGAACCCCCATTTTTCTCCTCATCTGCTCCACCATATCAGGGCCTAGAAATAGATATCAACCGATTGGACTTTGCTTTATTGCCTGACACGCCATTCTTACGCTGCGTACTCCTC
664	TGCTGTGTTGTCCCTCAATCTCGGCTGAGCTGCGCCAGCCGAATGGTTGGTCGAGTGGAATTACTAACTTGTGTTTCAGCGTGTTTCTTATTCATTTACCTCGCAGGGCCTGACACTCCCTTAGTTTTCAATTATCGAACCCCCATTTTTCTCCTCATCTGCTCCACCATATCAGGGCCTAGAAATAGATATCAACCGATTGGACTTTGCTTTATTGCCTGACACGCCATTCTTACGCTGCGTACTCCTC
ATCC 26197 (GA-1)	TGCTGTGTTGTCCCTCAATCTCGGCTGAGCTGCGCCAGCCGAATGGTTGGTCGAGTGGAATTACTAACTTGTGTTTCAGCGTGTTTCTTATTCATTTACCTCGCAGGGCCTGACACTCCCTTAGTTTTCAATTATCGAACCCCCATTTTTCTCCTCATCTGCTCCACCATATCAGGGCCTAGAAATAGATATCAACCGATTGGACTTTGCTTTATTGCCTGACACGCCATTCTTACGCTGCGTACTCCTC
ATCC 26199 (V)	TGCTGTGTTGTCCCTCAATCTCGGCTGAGCTGCGCCAGCCGAATGGTTGGTCGAGTGGAATTACTAACTTGTGTTTCAGCGTGTTTCTTATTCATTTACCTCGCAGGGCCTGACACTCCCTTAGTTTTCAATTATCGAACCCCCATTTTTCTCCTCATCTGCTCCACCATATCAGGGCCTAGAAATAGATATCAACCGATTGGACTTTGCTTTATTGCCTGACACGCCATTCTTACGCTGCGTACTCCTC
ATCC 28306	TGCTGTGTTGTCCCTCAATCTCGGCTGAGCTGCGCCAGCCGAATGGTTGGTCGAGTGGAATTACTAACTTGTGTTTCAGCGTGTTTCTTATTCATTTACCTCGCAGGGCCTGACACTCCCTTAGTTTTCAATTATCGAACCCCCATTTTTCTCCTCATCTGCTCCACCATATCAGGGCCTAGAAATAGATATCAACCGATTGGACTTTGCTTTATTGCCTGACACGCCATTCTTACGCTGCGTACTCCTC
ATCC MYA-2586 (ER-3)	TGCTGTGTTGTCCCTCAATCTCGGCTGAGCTGCGCCAGCCGAATGGTTGGTCGAGTGGAATTACTAACTTGTGTTTCAGCGTGTTTCTTATTCATTTACCTCGCAGGGCCTGACACTCCCTTAGTTTTCAATTATCGAACCCCCATTTTTCTCCTCATCTGCTCCACCATATCAGGGCCTAGAAATAGATATCAACCGATTGGACTTTGCTTTATTGCCTGACACGCCATTCTTACGCTGCGTACTCCTC
F270	TGCTGTGTTGTCCCTCAATCTCGGCTGAGCTGCGCCAGCCGAATGGTTGGTCGAGTGGAATTACTAACTTGTGTTTCAGCGTGTTTCTTATTCATTTACCTCGCAGGGCCTGACACTCCCTTAGTTTTCAATTATCGAACCCCCATTTTTCTCCTCATCTGCTCCACCATATCAGGGCCTAGAAATAGATATCAACCGATTGGACTTTGCTTTATTGCCTGACACGCCATTCTTACGCTGCGTACTCCTC
TB00042 2005	TGCTGTGTTGTCCCTCAATCTCGGCTGAGCTGCGCCAGCCGAATGGTTGGTCGAGTGGAATTACTAACTTGTGTTTCAGCGTGTTTCTTATTCATTTACCTCGCAGGGCCTGACACTCCCTTAGTTTTCAATTATCGAACCCCCATTTTTCTCCTCATCTGCTCCACCATATCAGGGCCTAGAAATAGATATCAACCGATTGGACTTTGCTTTATTGCCTGACACGCCATTCTTACGCTGCGTACTCCTC
UAMH 5584	TGCTGTGTTGTCCCTCAATCTCGGCTGAGCTGCGCCAGCCGAATGGTTGGTCGAGTGGAATTACTAACTTGTGTTTCAGCGTGTTTCTTATTCATTTACCTCGCAGGGCCTGACACTCCCTTAGTTTTCAATTATCGAACCCCCATTTTTCTCCTCATCTGCTCCACCATATCAGGGCCTAGAAATAGATATCAACCGATTGGACTTTGCTTTATTGCCTGACACGCCATTCTTACGCTGCGTACTCCTC
UAMH 5634	TGCTGTGTTGTCCCTCAATCTCGGCTGAGCTGCGCCAGCCGAATGGTTGGTCGAGTGGAATTACTAACTTGTGTTTCAGCGTGTTTCTTATTCATTTACCTCGCAGGGCCTGACACTCCCTTAGTTTTCAATTATCGAACCCCCATTTTTCTCCTCATCTGCTCCACCATATCAGGGCCTAGAAATAGATATCAACCGATTGGACTTTGCTTTATTGCCTGACACGCCATTCTTACGCTGCGTACTCCTC
UAMH 4042	TGCTGTGTTGTCCCTCAATCTCGGCTGAGCTGCGCCAGCCGAATGGTTGGTCGAGTGGAATTACTAACTTGTGTTTCAGCGTGTTTCTTATTCATTTACCTCGCACGGCCTGACACTCCCTTAGTTTTCAATTATCGAACCCCCATTTTTCTCCTCATCTGCTCCACCATATCAGGGCCTAGAAATAGATATCAACCGATTGGACTTTGCTTTATTGCCTGACACGCCATTCTTACGCTGCGTACTCCTC
ATCC 62541 (601)	TGCTGTGTTGTCCCTCAATCTCGGCTGAGCTGCGCCAGCCGAATGGTTGGTCGAGTGGAATTACTAACTTGTGTTTCAGCGTGTTTCTTATTCATTTACCTCGCAGGGCCTGACACTCCCTTAGTTTTCAATTATCGAACACCCATTTTTCTCCTCATCTGCTCCACCATATCAGGGCCTAGAAATAGATATCAACCGATTGGACTTTGCTTTATTGCCTGACACGCCATTCTTACGCTACGTACTCCTC
B1566 (UAMH 12045)	TGCTGTGTTGTCCCTCAATCTCGGCTGAGCTGCGCCAGCCGAATGGTTGGTCGAGTGGAATTACTAACTTGTGTTTCAGCGTGTTTCTTATTCATTTACCTCGCAGGGCCTGACACTCCCTTAGTTTTCAATTATCGAACACCCATTTTTCTCCTCATCTGCTCCACCATATCAGGGCCTAGAAATAGATATCAACCGATTGGACTTTGCTTTATTGCCTGACACGCCATTCTTACGCTACGTACTCCTC
TB00016 2005	TGCTGTGTTGTCCCTCAATCTCGGCTGAGCTGCGCCAGCCGAATGGTTGGTCGAGTGGAATTACTAACTTGTGTTTCAGCGTGTTTCTTATTCATTTACCTCGCAGGGCCTGACACTCCCTTAGTTTTCAATTATCGAACACCCATTTTTCTCCTCATCTGCTCCACCATATCAGGGCCTAGAAATAGATATCAACCGATTGGACTTTGCTTTATTGCCTGACACGCCATTCTTACGCTACGTACTCCTC
TB00040 2005	TGCTGTGTTGTCCCTCAATCTCGGCTGAGCTGCGCCAGCCGAATGGTTGGTCGAGTGGAATTACTAACTTGTGTTTCAGCGTGTTTCTTATTCATTTACCTCGCAGGGCCTGACACTCCCTTAGTTTTCAATTATCGAACACCCATTTTTCTCCTCATCTGCTCCACCATATCAGGGCCTAGAAATAGATATCAACCGATTGGACTTTGCTTTATTGCCTGACACGCCATTCTTACGCTACGTACTCCTC

Figure S1: Nucleotide Sequence Alignments (continued)

chs2

INTRON / EXON

B1566 (UAMH 12045)	GAGCCTTCAGCCACGCAGCTAACAATACTTTCAGAGCCAAGAAGATGTTCATGACCGGCATGGTTGTCTATTGTATAATAATGATGTACACGGTCTTTTCCGCTCTGTATATGGTGGTGACCCAATTAAAGCTCAGTGACCAACCTCTCGAAGAACGGCTGAAGCTGGGCAACAATACCTTCACCAACATCATCGTCTCGACGCTCTCAACCGTCGGCCTATATTTCTTCATGTCCTTCCTCTACCTC
TB00016 2005	GAGCCTTCAGCCACGCAGCTAACAATACTTTCAGAGCCAAGAAGATGTTCATGACCGGCATGGTTGTCTATTGTATAATAATGATGTACACGGTCTTTTCCGCTCTGTATATGGTGGTGACCCAATTAAAGCTCAGTGACCAACCTCTCGAAGAACGGCTGAAGCTGGGCAACAATACCTTCACCAACATCATCGTCTCGACGCTCTCAACCGTCGGCCTATATTTCTTCATGTCCTTCCTCTACCTC
TB00040 2005	GAGCCTTCAGCCACGCAGCTAACAATACTTTCAGAGCCAAGAAGATGTTCATGACCGGCATGGTTGTCTATTGTATAATAATGATGTACACGGTCTTTTCCGCTCTGTATATGGTGGTGACCCAATTAAAGCTCAGTGACCAACCTCTCGAAGAACGGCTGAAGCTGGGCAACAATACCTTCACCAACATCATCGTCTCGACGCTCTCAACCGTCGGCCTATATTTCTTCATGTCCTTCCTCTACCTC
ATCC 62541 (601)	GAGCCTTCAGCCACGCAGCTAACAATACTTTCAGAGCCAAGAAGATGTTCATGACCGGCATGGTTGTCTATTGTATAATAATGATGTACACGGTCTTTTCCGCTCTGTATATGGTGGTGACCCAATTAAAGCTCAGTGACCAACCTCTCGAAGAACGGCTGAAGCTGGGCAACAATACCTTCACCAACATCATCGTCTCGACCCTCTCAACCGTCGGCCTATATTTCTTCATGTCCTTCCTCTACCTC
ATCC 66136 (637)	GAGCCTTCAGCCACGCAGCTAACAATACTTTCAGAGCCAAGAAGATGTTCATGACCGGCATGGTTGTCTATTGTATAATAATGATGTACACGGTCTTTTCCGCTCTGTATATGGTGGTGACCCAATTAAAGCTCAGTGACCAACCTCTCGAAGAACGGCTGAAGCTGGGCAACAATACCTTCACCAACATCATCGTCTCGACCCTCTCAACCGTCGGCCTATATTTCTTCATGTCCTTCCTCTACCTC
FR00742 2009	GAGCCTTCAGCCACGCAGCTAACAATACTTTCAGAGCCAAGAAGATGTTCATGACCGGCATGGTTGTCTATTGTATAATAATGATGTACACGGTCTTTTCCGCTCTGTATATGGTGGTGACCCAATTAAAGCTCAGTGACCAACCTCTCGAAGAACGGCTGAAGCTGGGCAACAATACCTTCACCAACATCATCGTCTCGACCCTCTCAACCGTCGGCCTATATTTCTTCATGTCCTTCCTCTACCTC
TB00024 2005	GAGCCTTCAGCCACGCAGCTAACAATACTTTCAGAGCCAAGAAGATGTTCATGACCGGCATGGTTGTCTATTGTATAATAATGATGTACACGGTCTTTTCCGCTCTGTATATGGTGGTGACCCAATTAAAGCTCAGTGACCAACCTCTCGAAGAACGGCTGAAGCTGGGCAACAATACCTTCACCAACATCATCGTCTCGACCCTCTCAACCGTCGGCCTATATTTCTTCATGTCCTTCCTCTACCTC
UAMH 5584	GAGCCTTCAGCCACGCAGCTAACAATACTTTCAGAGCCAAGAAGATGTTCATGACCGGCATGGTTGTCTATTGTATAATAATGATGTACACGGTCTTTTCCGCTCTGTATATGGTGGTGACCCAATTAAAGCTCAGTGACCAACCTCTCGAAGAACGGCTGAAGCTGGGCAACAATACCTTCACCAACATCATCGTCTCGACCCTCTCAACCGTCGGCCTATATTTCTTCATGTCCTTCCTCTACCTC
371	GAGCCTTCAGCCACGCAGCTAACAATACTTTCAGAGCCAAGAAGATGTTCATGACCGGCATGGTTGTCTATTGTATAATAATGATGTACACGGTCTTTTCCGCTCTGTATATGGTGGTGACCCAATTAAAGCTCAGTGACCAACCTCTCGAAGAACGGCTGAAGCTGGGCAACAATACCTTCACCAACATCATCGTCTCGACCCTCTCAACCGTCGGCCTATATTTCTTCATGTCCTTCCTCTACCTC
396	GAGCCTTCAGCCACGCAGCTAACAATACTTTCAGAGCCAAGAAGATGTTCATGACCGGCATGGTTGTCTATTGTATAATAATGATGTACACGGTCTTTTCCGCTCTGTATATGGTGGTGACCCAATTAAAGCTCAGTGACCAACCTCTCGAAGAACGGCTGAAGCTGGGCAACAATACCTTCACCAACATCATCGTCTCGACCCTCTCAACCGTCGGCCTATATTTCTTCATGTCCTTCCTCTACCTC
397	GAGCCTTCAGCCACGCAGCTAACAATACTTTCAGAGCCAAGAAGATGTTCATGACCGGCATGGTTGTCTATTGTATAATAATGATGTACACGGTCTTTTCCGCTCTGTATATGGTGGTGACCCAATTAAAGCTCAGTGACCAACCTCTCGAAGAACGGCTGAAGCTGGGCAACAATACCTTCACCAACATCATCGTCTCGACCCTCTCAACCGTCGGCCTATATTTCTTCATGTCCTTCCTCTACCTC
663	GAGCCTTCAGCCACGCAGCTAACAATACTTTCAGAGCCAAGAAGATGTTCATGACCGGCATGGTTGTCTATTGTATAATAATGATGTACACGGTCTTTTCCGCTCTGTATATGGTGGTGACCCAATTAAAGCTCAGTGACCAACCTCTCGAAGAACGGCTGAAGCTGGGCAACAATACCTTCACCAACATCATCGTCTCGACCCTCTCAACCGTCGGCCTATATTTCTTCATGTCCTTCCTCTACCTC
664	GAGCCTTCAGCCACGCAGCTAACAATACTTTCAGAGCCAAGAAGATGTTCATGACCGGCATGGTTGTCTATTGTATAATAATGATGTACACGGTCTTTTCCGCTCTGTATATGGTGGTGACCCAATTAAAGCTCAGTGACCAACCTCTCGAAGAACGGCTGAAGCTGGGCAACAATACCTTCACCAACATCATCGTCTCGACCCTCTCAACCGTCGGCCTATATTTCTTCATGTCCTTCCTCTACCTC
ATCC 26199 (V)	GAGCCTTCAGCCACGCAGCTAACAATACTTTCAGAGCCAAGAAGATGTTCATGACCGGCATGGTTGTCTATTGTATAATAATGATGTACACGGTCTTTTCCGCTCTGTATATGGTGGTGACCCAATTAAAGCTCAGTGACCAACCTCTCGAAGAACGGCTGAAGCTGGGCAACAATACCTTCACCAACATCATCGTCTCGACCCTCTCAACCGTCGGCCTATATTTCTTCATGTCCTTCCTCTACCTC
ATCC MYA-2586 (ER-3)	GAGCCTTCAGCCACGCAGCTAACAATACTTTCAGAGCCAAGAAGATGTTCATGACCGGCATGGTTGTCTATTGTATAATAATGATGTACACGGTCTTTTCCGCTCTGTATATGGTGGTGACCCAATTAAAGCTCAGTGACCAACCTCTCGAAGAACGGCTGAAGCTGGGCAACAATACCTTCACCAACATCATCGTCTCGACCCTCTCAACCGTCGGCCTATATTTCTTCATGTCCTTCCTCTACCTC
B3003 (UAMH 12046)	GAGCCTTCAGCCACGCAGCTAACAATACTTTCAGAGCCAAGAAGATGTTCATGACCGGCATGGTTGTCTATTGTATAATAATGATGTACACGGTCTTTTCCGCTCTGTATATGGTGGTGACCCAATTAAAGCTCAGTGACCAACCTCTCGAAGAACGGCTGAAGCTGGGCAACAATACCTTCACCAACATCATCGTCTCGACCCTCTCAACCGTCGGCCTATATTTCTTCATGTCCTTCCTCTACCTC
CH-10	GAGCCTTCAGCCACGCAGCTAACAATACTTTCAGAGCCAAGAAGATGTTCATGACCGGCATGGTTGTCTATTGTATAATAATGATGTACACGGTCTTTTCCGCTCTGTATATGGTGGTGACCCAATTAAAGCTCAGTGACCAACCTCTCGAAGAACGGCTGAAGCTGGGCAACAATACCTTCACCAACATCATCGTCTCGACCCTCTCAACCGTCGGCCTATATTTCTTCATGTCCTTCCTCTACCTC
En	GAGCCTTCAGCCACGCAGCTAACAATACTTTCAGAGCCAAGAAGATGTTCATGACCGGCATGGTTGTCTATTGTATAATAATGATGTACACGGTCTTTTCCGCTCTGTATATGGTGGTGACCCAATTAAAGCTCAGTGACCAACCTCTCGAAGAACGGCTGAAGCTGGGCAACAATACCTTCACCAACATCATCGTCTCGACCCTCTCAACCGTCGGCCTATATTTCTTCATGTCCTTCCTCTACCTC
F270	GAGCCTTCAGCCACGCAGCTAACAATACTTTCAGAGCCAAGAAGATGTTCATGACCGGCATGGTTGTCTATTGTATAATAATGATGTACACGGTCTTTTCCGCTCTGTATATGGTGGTGACCCAATTAAAGCTCAGTGACCAACCTCTCGAAGAACGGCTGAAGCTGGGCAACAATACCTTCACCAACATCATCGTCTCGACCCTCTCAACCGTCGGCCTATATTTCTTCATGTCCTTCCTCTACCTC
FR00346 2009	GAGCCTTCAGCCACGCAGCTAACAATACTTTCAGAGCCAAGAAGATGTTCATGACCGGCATGGTTGTCTATTGTATAATAATGATGTACACGGTCTTTTCCGCTCTGTATATGGTGGTGACCCAATTAAAGCTCAGTGACCAACCTCTCGAAGAACGGCTGAAGCTGGGCAACAATACCTTCACCAACATCATCGTCTCGACCCTCTCAACCGTCGGCCTATATTTCTTCATGTCCTTCCTCTACCTC
K940	GAGCCTTCAGCCACGCAGCTAACAATACTTTCAGAGCCAAGAAGATGTTCATGACCGGCATGGTTGTCTATTGTATAATAATGATGTACACGGTCTTTTCCGCTCTGTATATGGTGGTGACCCAATTAAAGCTCAGTGACCAACCTCTCGAAGAACGGCTGAAGCTGGGCAACAATACCTTCACCAACATCATCGTCTCGACCCTCTCAACCGTCGGCCTATATTTCTTCATGTCCTTCCTCTACCTC
Ro	GAGCCTTCAGCCACGCAGCTAACAATACTTTCAGAGCCAAGAAGATGTTCATGACCGGCATGGTTGTCTATTGTATAATAATGATGTACACGGTCTTTTCCGCTCTGTATATGGTGGTGACCCAATTAAAGCTCAGTGACCAACCTCTCGAAGAACGGCTGAAGCTGGGCAACAATACCTTCACCAACATCATCGTCTCGACCCTCTCAACCGTCGGCCTATATTTCTTCATGTCCTTCCTCTACCTC
SF03443 2009	GAGCCTTCAGCCACGCAGCTAACAATACTTTCAGAGCCAAGAAGATGTTCATGACCGGCATGGTTGTCTATTGTATAATAATGATGTACACGGTCTTTTCCGCTCTGTATATGGTGGTGACCCAATTAAAGCTCAGTGACCAACCTCTCGAAGAACGGCTGAAGCTGGGCAACAATACCTTCACCAACATCATCGTCTCGACCCTCTCAACCGTCGGCCTATATTTCTTCATGTCCTTCCTCTACCTC
SF05773 2009	GAGCCTTCAGCCACGCAGCTAACAATACTTTCAGAGCCAAGAAGATGTTCATGACCGGCATGGTTGTCTATTGTATAATAATGATGTACACGGTCTTTTCCGCTCTGTATATGGTGGTGACCCAATTAAAGCTCAGTGACCAACCTCTCGAAGAACGGCTGAAGCTGGGCAACAATACCTTCACCAACATCATCGTCTCGACCCTCTCAACCGTCGGCCTATATTTCTTCATGTCCTTCCTCTACCTC
SF05792 2009	GAGCCTTCAGCCACGCAGCTAACAATACTTTCAGAGCCAAGAAGATGTTCATGACCGGCATGGTTGTCTATTGTATAATAATGATGTACACGGTCTTTTCCGCTCTGTATATGGTGGTGACCCAATTAAAGCTCAGTGACCAACCTCTCGAAGAACGGCTGAAGCTGGGCAACAATACCTTCACCAACATCATCGTCTCGACCCTCTCAACCGTCGGCCTATATTTCTTCATGTCCTTCCTCTACCTC
SF06072 2009	GAGCCTTCAGCCACGCAGCTAACAATACTTTCAGAGCCAAGAAGATGTTCATGACCGGCATGGTTGTCTATTGTATAATAATGATGTACACGGTCTTTTCCGCTCTGTATATGGTGGTGACCCAATTAAAGCTCAGTGACCAACCTCTCGAAGAACGGCTGAAGCTGGGCAACAATACCTTCACCAACATCATCGTCTCGACCCTCTCAACCGTCGGCCTATATTTCTTCATGTCCTTCCTCTACCTC
SF06266 2009	GAGCCTTCAGCCACGCAGCTAACAATACTTTCAGAGCCAAGAAGATGTTCATGACCGGCATGGTTGTCTATTGTATAATAATGATGTACACGGTCTTTTCCGCTCTGTATATGGTGGTGACCCAATTAAAGCTCAGTGACCAACCTCTCGAAGAACGGCTGAAGCTGGGCAACAATACCTTCACCAACATCATCGTCTCGACCCTCTCAACCGTCGGCCTATATTTCTTCATGTCCTTCCTCTACCTC
SF06354 2009	GAGCCTTCAGCCACGCAGCTAACAATACTTTCAGAGCCAAGAAGATGTTCATGACCGGCATGGTTGTCTATTGTATAATAATGATGTACACGGTCTTTTCCGCTCTGTATATGGTGGTGACCCAATTAAAGCTCAGTGACCAACCTCTCGAAGAACGGCTGAAGCTGGGCAACAATACCTTCACCAACATCATCGTCTCGACCCTCTCAACCGTCGGCCTATATTTCTTCATGTCCTTCCTCTACCTC
SF14600 2008	GAGCCTTCAGCCACGCAGCTAACAATACTTTCAGAGCCAAGAAGATGTTCATGACCGGCATGGTTGTCTATTGTATAATAATGATGTACACGGTCTTTTCCGCTCTGTATATGGTGGTGACCCAATTAAAGCTCAGTGACCAACCTCTCGAAGAACGGCTGAAGCTGGGCAACAATACCTTCACCAACATCATCGTCTCGACCCTCTCAACCGTCGGCCTATATTTCTTCATGTCCTTCCTCTACCTC
TB00011 2006	GAGCCTTCAGCCACGCAGCTAACAATACTTTCAGAGCCAAGAAGATGTTCATGACCGGCATGGTTGTCTATTGTATAATAATGATGTACACGGTCTTTTCCGCTCTGTATATGGTGGTGACCCAATTAAAGCTCAGTGACCAACCTCTCGAAGAACGGCTGAAGCTGGGCAACAATACCTTCACCAACATCATCGTCTCGACCCTCTCAACCGTCGGCCTATATTTCTTCATGTCCTTCCTCTACCTC
TB00042 2005	GAGCCTTCAGCCACGCAGCTAACAATACTTTCAGAGCCAAGAAGATGTTCATGACCGGCATGGTTGTCTATTGTATAATAATGATGTACACGGTCTTTTCCGCTCTGTATATGGTGGTGACCCAATTAAAGCTCAGTGACCAACCTCTCGAAGAACGGCTGAAGCTGGGCAACAATACCTTCACCAACATCATCGTCTCGACCCTCTCAACCGTCGGCCTATATTTCTTCATGTCCTTCCTCTACCTC
UAMH 4042	GAGCCTTCAGCCACGCAGCTAACAATACTTTCAGAGCCAAGAAGATGTTCATGACCGGCATGGTTGTCTATTGTATAATAATGATGTACACGGTCTTTTCCGCTCTGTATATGGTGGTGACCCAATTAAAGCTCAGTGACCAACCTCTCGAAGAACGGCTGAAGCTGGGCAACAATACCTTCACCAACATCATCGTCTCGACCCTCTCAACCGTCGGCCTATATTTCTTCATGTCCTTCCTCTACCTC
UAMH 5634	GAGCCTTCAGCCACGCAGCTAACAATACTTTCAGAGCCAAGAAGATGTTCATGACCGGCATGGTTGTCTATTGTATAATAATGATGTACACGGTCTTTTCCGCTCTGTATATGGTGGTGACCCAATTAAAGCTCAGTGACCAACCTCTCGAAGAACGGCTGAAGCTGGGCAACAATACCTTCACCAACATCATCGTCTCGACCCTCTCAACCGTCGGCCTATATTTCTTCATGTCCTTCCTCTACCTC
UAMH 7800	GAGCCTTCAGCCACGCAGCTAACAATACTTTCAGAGCCAAGAAGATGTTCATGACCGGCATGGTTGTCTATTGTATAATAATGATGTACACGGTCTTTTCCGCTCTGTATATGGTGGTGACCCAATTAAAGCTCAGTGACCAACCTCTCGAAGAACGGCTGAAGCTGGGCAACAATACCTTCACCAACATCATCGTCTCGACCCTCTCAACCGTCGGCCTATATTTCTTCATGTCCTTCCTCTACCTC
ATCC 26197 (GA-1)	GAGCCTTCAGCCACACAGCTAACAATACTTTCAGAGCCAAGAAGATGTTCATGACCGGCATGGTTGTCTATTGTATAATAATGATGTACACGGTCTTTTCCGCTCTGTATATGGTGGTGACCCAATTAAAGCTCAGTGACCAACCTCTCGAAGAACGGCTGAAGCTGGGCAACAATACCTTCACCAACATCATCGTCTCGACCCTCTCAACCGTCGGCCTATATTTCTTCATGTCCTTCCTCTACCTC
ATCC 28306	GAGCCTTCAGCCACACAGCTAACAATACTTTCAGAGCCAAGAAGATGTTCATGACCGGCATGGTTGTCTATTGTATAATAATGATGTACACGGTCTTTTCCGCTCTGTATATGGTGGTGACCCAATTAAAGCTCAGTGACCAACCTCTCGAAGAACGGCTGAAGCTGGGCAACAATACCTTCACCAACATCATCGTCTCGACCCTCTCAACCGTCGGCCTATATTTCTTCATGTCCTTCCTCTACCTC

chs2 (continued)

EXON

B1566 (UAMH 12045)	GATCCCTGGCACATGTTCACCTCGTCAGCGCAATACTTCGCCCTACTCCCTAGCTACATCTGCACCTTGCAGGTCTATGCCTTCTGCAACACCCACGACGTCACGTGGGGCACCAAGGGCGATAACATGATCCACACCGACCTCGGCGCCGCGCGCACCACGGGCTCCTCCACTGTCGAGCTGGAAATGCCCTCGGAGCAACTGGACATCGACAGCGGCTACGACGAGGCGCTACGGAACCTACGC
TB00016 2005	GATCCCTGGCACATGTTCACCTCGTCAGCGCAATACTTCGCCCTACTCCCTAGCTACATCTGCACCTTGCAGGTCTATGCCTTCTGCAACACCCACGACGTCACGTGGGGCACCAAGGGCGATAACATGATCCACACCGACCTCGGCGCCGCGCGCACCACGGGCTCCTCCACTGTCGAGCTGGAAATGCCCTCGGAGCAACTGGACATCGACAGCGGCTACGACGAGGCGCTACGGAACCTACGC
TB00040 2005	GATCCCTGGCACATGTTCACCTCGTCAGCGCAATACTTCGCCCTACTCCCTAGCTACATCTGCACCTTGCAGGTCTATGCCTTCTGCAACACCCACGACGTCACGTGGGGCACCAAGGGCGATAACATGATCCACACCGACCTCGGCGCCGCGCGCACCACGGGCTCCTCCACTGTCGAGCTGGAAATGCCCTCGGAGCAACTGGACATCGACAGCGGCTACGACGAGGCGCTACGGAACCTACGC
ATCC 62541 (601)	GATCCCTGGCACATGTTCACCTCGTCAGCGCAATACTTCGCCCTACTCCCTAGCTACATCTGCACCTTGCAGGTCTATGCCTTCTGCAACACCCACGACGTCACGTGGGGCACCAAGGGCGATAACATGATCCACACCGACCTCGGCGCCGCGCGCACCACGGGCTCCTCCACTGTCGAGCTGGAAATGCCCTCGGAGCAACTGGACATCGACAGCGGCTACGACGAGGCGCTACGGAACCTACGC
ATCC 66136 (637)	GATCCCTGGCACATGTTCACCTCGTCAGCGCAATACTTCGCCCTACTCCCTAGCTACATCTGCACCTTGCAGGTCTATGCCTTCTGCAACACCCACGACGTCACGTGGGGCACCAAGGGCGATAACATGATCCACACCGACCTCGGCGCCGCGCGCACCACGGGCTCCTCCACTGTCGAGCTGGAAATGCCCTCGGAGCAACTGGACATCGACAGCGGCTACGACGAGGCGCTACGGAACCTACGC
FR00742 2009	GATCCCTGGCACATGTTCACCTCGTCAGCGCAATACTTCGCCCTACTCCCTAGCTACATCTGCACCTTGCAGGTCTATGCCTTCTGCAACACCCACGACGTCACGTGGGGCACCAAGGGCGATAACATGATCCACACCGACCTCGGCGCCGCGCGCACCACGGGCTCCTCCACTGTCGAGCTGGAAATGCCCTCGGAGCAACTGGACATCGACAGCGGCTACGACGAGGCGCTACGGAACCTACGC
TB00024 2005	GATCCCTGGCACATGTTCACCTCGTCAGCGCAATACTTCGCGCTACTCCCTAGCTACATCTGCACCTTGCAGGTCTATGCCTTCTGCAACACCCACGACGTCACGTGGGGCACCAAGGGCGATAACATGATCCACACCGACCTCGGCGCCGCGCGCACCACGGGCTCCTCCACCGTCGAGCTGGAAATGCCCTCGGAGCAACTGGACATCGATAGCGGCTACGACGAGGCGCTACGGAACCTGCGC
UAMH 5584	GATCCCTGGCACATGTTCACCTCGTCAGCGCAATACTTCGCGCTACTCCCTAGCTACATCTGCACCTTGCAGGTCTATGCCTTCTGCAACACCCACGACGTCACGTGGGGCACCAAGGGCGATAACATGATCCACACCGACCTCGGCGCCGCGCGCACCACGGGCTCCTCCACCGTCGAGCTGGAAATGCCCTCGGAGCAACTGGACATCGATAGCGGCTACGACGAGGCGCTACGGAACCTGCGC
371	GATCCCTGGCACATGTTCACCTCGTCAGCGCAATACTTCGCCCTACTCCCTAGCTACATCTGCACCTTGCAGGTCTATGCCTTCTGCAACACCCACGACGTCACGTGGGGCACCAAGGGCGATAACATGATCCACACCGACCTCGGCGCCGCGCGCACCACGGGCTCCTCCACCGTCGAGCTGGAAATGCCCTCGGAGCAACTGGACATCGATAGCGGCTACGACGAGGCGCTACGGAACCTGCGC
396	GATCCCTGGCACATGTTCACCTCGTCAGCGCAATACTTCGCCCTACTCCCTAGCTACATCTGCACCTTGCAGGTCTATGCCTTCTGCAACACCCACGACGTCACGTGGGGCACCAAGGGCGATAACATGATCCACACCGACCTCGGCGCCGCGCGCACCACGGGCTCCTCCACCGTCGAGCTGGAAATGCCCTCGGAGCAACTGGACATCGATAGCGGCTACGACGAGGCGCTACGGAACCTGCGC
397	GATCCCTGGCACATGTTCACCTCGTCAGCGCAATACTTCGCCCTACTCCCTAGCTACATCTGCACCTTGCAGGTCTATGCCTTCTGCAACACCCACGACGTCACGTGGGGCACCAAGGGCGATAACATGATCCACACCGACCTCGGCGCCGCGCGCACCACGGGCTCCTCCACCGTCGAGCTGGAAATGCCCTCGGAGCAACTGGACATCGATAGCGGCTACGACGAGGCGCTACGGAACCTGCGC
663	GATCCCTGGCACATGTTCACCTCGTCAGCGCAATACTTCGCCCTACTCCCTAGCTACATCTGCACCTTGCAGGTCTATGCCTTCTGCAACACCCACGACGTCACGTGGGGCACCAAGGGCGATAACATGATCCACACCGACCTCGGCGCCGCGCGCACCACGGGCTCCTCCACCGTCGAGCTGGAAATGCCCTCGGAGCAACTGGACATCGATAGCGGCTACGACGAGGCGCTACGGAACCTGCGC
664	GATCCCTGGCACATGTTCACCTCGTCAGCGCAATACTTCGCCCTACTCCCTAGCTACATCTGCACCTTGCAGGTCTATGCCTTCTGCAACACCCACGACGTCACGTGGGGCACCAAGGGCGATAACATGATCCACACCGACCTCGGCGCCGCGCGCACCACGGGCTCCTCCACCGTCGAGCTGGAAATGCCCTCGGAGCAACTGGACATCGATAGCGGCTACGACGAGGCGCTACGGAACCTGCGC
ATCC 26199 (V)	GATCCCTGGCACATGTTCACCTCGTCAGCGCAATACTTCGCCCTACTCCCTAGCTACATCTGCACCTTGCAGGTCTATGCCTTCTGCAACACCCACGACGTCACGTGGGGCACCAAGGGCGATAACATGATCCACACCGACCTCGGCGCCGCGCGCACCACGGGCTCCTCCACCGTCGAGCTGGAAATGCCCTCGGAGCAACTGGACATCGATAGCGGCTACGACGAGGCGCTACGGAACCTGCGC
ATCC MYA-2586 (ER-3)	GATCCCTGGCACATGTTCACCTCGTCAGCGCAATACTTCGCCCTACTCCCTAGCTACATCTGCACCTTGCAGGTCTATGCCTTCTGCAACACCCACGACGTCACGTGGGGCACCAAGGGCGATAACATGATCCACACCGACCTCGGCGCCGCGCGCACCACGGGCTCCTCCACCGTCGAGCTGGAAATGCCCTCGGAGCAACTGGACATCGATAGCGGCTACGACGAGGCGCTACGGAACCTGCGC
B3003 (UAMH 12046)	GATCCCTGGCACATGTTCACCTCGTCAGCGCAATACTTCGCCCTACTCCCTAGCTACATCTGCACCTTGCAGGTCTATGCCTTCTGCAACACCCACGACGTCACGTGGGGCACCAAGGGCGATAACATGATCCACACCGACCTCGGCGCCGCGCGCACCACGGGCTCCTCCACCGTCGAGCTGGAAATGCCCTCGGAGCAACTGGACATCGATAGCGGCTACGACGAGGCGCTACGGAACCTGCGC
CH-10	GATCCCTGGCACATGTTCACCTCGTCAGCGCAATACTTCGCCCTACTCCCTAGCTACATCTGCACCTTGCAGGTCTATGCCTTCTGCAACACCCACGACGTCACGTGGGGCACCAAGGGCGATAACATGATCCACACCGACCTCGGCGCCGCGCGCACCACGGGCTCCTCCACCGTCGAGCTGGAAATGCCCTCGGAGCAACTGGACATCGATAGCGGCTACGACGAGGCGCTACGGAACCTGCGC
En	GATCCCTGGCACATGTTCACCTCGTCAGCGCAATACTTCGCCCTACTCCCTAGCTACATCTGCACCTTGCAGGTCTATGCCTTCTGCAACACCCACGACGTCACGTGGGGCACCAAGGGCGATAACATGATCCACACCGACCTCGGCGCCGCGCGCACCACGGGCTCCTCCACCGTCGAGCTGGAAATGCCCTCGGAGCAACTGGACATCGATAGCGGCTACGACGAGGCGCTACGGAACCTGCGC
F270	GATCCCTGGCACATGTTCACCTCGTCAGCGCAATACTTCGCCCTACTCCCTAGCTACATCTGCACCTTGCAGGTCTATGCCTTCTGCAACACCCACGACGTCACGTGGGGCACCAAGGGCGATAACATGATCCACACCGACCTCGGCGCCGCGCGCACCACGGGCTCCTCCACCGTCGAGCTGGAAATGCCCTCGGAGCAACTGGACATCGATAGCGGCTACGACGAGGCGCTACGGAACCTGCGC
FR00346 2009	GATCCCTGGCACATGTTCACCTCGTCAGCGCAATACTTCGCCCTACTCCCTAGCTACATCTGCACCTTGCAGGTCTATGCCTTCTGCAACACCCACGACGTCACGTGGGGCACCAAGGGCGATAACATGATCCACACCGACCTCGGCGCCGCGCGCACCACGGGCTCCTCCACCGTCGAGCTGGAAATGCCCTCGGAGCAACTGGACATCGATAGCGGCTACGACGAGGCGCTACGGAACCTGCGC
K940	GATCCCTGGCACATGTTCACCTCGTCAGCGCAATACTTCGCCCTACTCCCTAGCTACATCTGCACCTTGCAGGTCTATGCCTTCTGCAACACCCACGACGTCACGTGGGGCACCAAGGGCGATAACATGATCCACACCGACCTCGGCGCCGCGCGCACCACGGGCTCCTCCACCGTCGAGCTGGAAATGCCCTCGGAGCAACTGGACATCGATAGCGGCTACGACGAGGCGCTACGGAACCTGCGC
Ro	GATCCCTGGCACATGTTCACCTCGTCAGCGCAATACTTCGCCCTACTCCCTAGCTACATCTGCACCTTGCAGGTCTATGCCTTCTGCAACACCCACGACGTCACGTGGGGCACCAAGGGCGATAACATGATCCACACCGACCTCGGCGCCGCGCGCACCACGGGCTCCTCCACCGTCGAGCTGGAAATGCCCTCGGAGCAACTGGACATCGATAGCGGCTACGACGAGGCGCTACGGAACCTGCGC
SF03443 2009	GATCCCTGGCACATGTTCACCTCGTCAGCGCAATACTTCGCCCTACTCCCTAGCTACATCTGCACCTTGCAGGTCTATGCCTTCTGCAACACCCACGACGTCACGTGGGGCACCAAGGGCGATAACATGATCCACACCGACCTCGGCGCCGCGCGCACCACGGGCTCCTCCACCGTCGAGCTGGAAATGCCCTCGGAGCAACTGGACATCGATAGCGGCTACGACGAGGCGCTACGGAACCTGCGC
SF05773 2009	GATCCCTGGCACATGTTCACCTCGTCAGCGCAATACTTCGCCCTACTCCCTAGCTACATCTGCACCTTGCAGGTCTATGCCTTCTGCAACACCCACGACGTCACGTGGGGCACCAAGGGCGATAACATGATCCACACCGACCTCGGCGCCGCGCGCACCACGGGCTCCTCCACCGTCGAGCTGGAAATGCCCTCGGAGCAACTGGACATCGATAGCGGCTACGACGAGGCGCTACGGAACCTGCGC
SF05792 2009	GATCCCTGGCACATGTTCACCTCGTCAGCGCAATACTTCGCCCTACTCCCTAGCTACATCTGCACCTTGCAGGTCTATGCCTTCTGCAACACCCACGACGTCACGTGGGGCACCAAGGGCGATAACATGATCCACACCGACCTCGGCGCCGCGCGCACCACGGGCTCCTCCACCGTCGAGCTGGAAATGCCCTCGGAGCAACTGGACATCGATAGCGGCTACGACGAGGCGCTACGGAACCTGCGC
SF06072 2009	GATCCCTGGCACATGTTCACCTCGTCAGCGCAATACTTCGCCCTACTCCCTAGCTACATCTGCACCTTGCAGGTCTATGCCTTCTGCAACACCCACGACGTCACGTGGGGCACCAAGGGCGATAACATGATCCACACCGACCTCGGCGCCGCGCGCACCACGGGCTCCTCCACCGTCGAGCTGGAAATGCCCTCGGAGCAACTGGACATCGATAGCGGCTACGACGAGGCGCTACGGAACCTGCGC
SF06266 2009	GATCCCTGGCACATGTTCACCTCGTCAGCGCAATACTTCGCCCTACTCCCTAGCTACATCTGCACCTTGCAGGTCTATGCCTTCTGCAACACCCACGACGTCACGTGGGGCACCAAGGGCGATAACATGATCCACACCGACCTCGGCGCCGCGCGCACCACGGGCTCCTCCACCGTCGAGCTGGAAATGCCCTCGGAGCAACTGGACATCGATAGCGGCTACGACGAGGCGCTACGGAACCTGCGC
SF06354 2009	GATCCCTGGCACATGTTCACCTCGTCAGCGCAATACTTCGCCCTACTCCCTAGCTACATCTGCACCTTGCAGGTCTATGCCTTCTGCAACACCCACGACGTCACGTGGGGCACCAAGGGCGATAACATGATCCACACCGACCTCGGCGCCGCGCGCACCACGGGCTCCTCCACCGTCGAGCTGGAAATGCCCTCGGAGCAACTGGACATCGATAGCGGCTACGACGAGGCGCTACGGAACCTGCGC
SF14600 2008	GATCCCTGGCACATGTTCACCTCGTCAGCGCAATACTTCGCCCTACTCCCTAGCTACATCTGCACCTTGCAGGTCTATGCCTTCTGCAACACCCACGACGTCACGTGGGGCACCAAGGGCGATAACATGATCCACACCGACCTCGGCGCCGCGCGCACCACGGGCTCCTCCACCGTCGAGCTGGAAATGCCCTCGGAGCAACTGGACATCGATAGCGGCTACGACGAGGCGCTACGGAACCTGCGC
TB00011 2006	GATCCCTGGCACATGTTCACCTCGTCAGCGCAATACTTCGCCCTACTCCCTAGCTACATCTGCACCTTGCAGGTCTATGCCTTCTGCAACACCCACGACGTCACGTGGGGCACCAAGGGCGATAACATGATCCACACCGACCTCGGCGCCGCGCGCACCACGGGCTCCTCCACCGTCGAGCTGGAAATGCCCTCGGAGCAACTGGACATCGATAGCGGCTACGACGAGGCGCTACGGAACCTGCGC
TB00042 2005	GATCCCTGGCACATGTTCACCTCGTCAGCGCAATACTTCGCCCTACTCCCTAGCTACATCTGCACCTTGCAGGTCTATGCCTTCTGCAACACCCACGACGTCACGTGGGGCACCAAGGGCGATAACATGATCCACACCGACCTCGGCGCCGCGCGCACCACGGGCTCCTCCACCGTCGAGCTGGAAATGCCCTCGGAGCAACTGGACATCGATAGCGGCTACGACGAGGCGCTACGGAACCTGCGC
UAMH 4042	GATCCCTGGCACATGTTCACCTCGTCAGCGCAATACTTCGCCCTACTCCCTAGCTACATCTGCACCTTGCAGGTCTATGCCTTCTGCAACACCCACGACGTCACGTGGGGCACCAAGGGCGATAACATGATCCACACCGACCTCGGCGCCGCGCGCACCACGGGCTCCTCCACCGTCGAGCTGGAAATGCCCTCGGAGCAACTGGACATCGATAGCGGCTACGACGAGGCGCTACGGAACCTGCGC
UAMH 5634	GATCCCTGGCACATGTTCACCTCGTCAGCGCAATACTTCGCCCTACTCCCTAGCTACATCTGCACCTTGCAGGTCTATGCCTTCTGCAACACCCACGACGTCACGTGGGGCACCAAGGGCGATAACATGATCCACACCGACCTCGGCGCCGCGCGCACCACGGGCTCCTCCACCGTCGAGCTGGAAATGCCCTCGGAGCAACTGGACATCGATAGCGGCTACGACGAGGCGCTACGGAACCTGCGC
UAMH 7800	GATCCCTGGCACATGTTCACCTCGTCAGCGCAATACTTCGCCCTACTCCCTAGCTACATCTGCACCTTGCAGGTCTATGCCTTCTGCAACACCCACGACGTCACGTGGGGCACCAAGGGCGATAACATGATCCACACCGACCTCGGCGCCGCGCGCACCACGGGCTCCTCCACCGTCGAGCTGGAAATGCCCTCGGAGCAACTGGACATCGATAGCGGCTACGACGAGGCGCTACGGAACCTGCGC
ATCC 26197 (GA-1)	GATCCCTGGCACATGTTCACCTCGTCAGCGCAATACTTCGCCCTACTCCCTAGCTACATCTGCACCTTGCAGGTCTATGCCTTCTGCAACACCCACGACGTCACGTGGGGCACCAAGGGCGATAACATGATCCACACCGACCTCGGCGCCGCGCGCACCACGGGCTCCTCCACCGTCGAGCTGGAAATGCCCTCGGAGCAACTGGACATCGATAGCGGCTACGACGAGGCGCTACGGAACCTGCGC
ATCC 28306	GATCCCTGGCACATGTTCACCTCGTCAGCGCAATACTTCGCCCTACTCCCTAGCTACATCTGCACCTTGCAGGTCTATGCCTTCTGCAACACCCACGACGTCACGTGGGGCACCAAGGGCGATAACATGATCCACACCGACCTCGGCGCCGCGCGCACCACGGGCTCCTCCACCGTCGAGCTGGAAATGCCCTCGGAGCAACTGGACATCGATAGCGGCTACGACGAGGCGCTACGGAACCTGCGC

Figure S1: Nucleotide Sequence Alignments (continued)

chs2 (continued)

	EXON
B1566 (UAMH 12045)	GACCGGCTCGAGGTGGCCCCGCCCGAGCTCTCAGAGGCGCAGATGCAGGAAGACTACTACCGCGCCGTGCGCACGTAC
TB00016 2005	GACCGGCTCGAGGTGGCCCCGCCCGAGCTCTCAGAGGCGCAGATGCAGGAAGACTACTACCGCGCCGTGCGCACGTAC
TB00040 2005	GACCGGCTCGAGGTGGCCCCGCCCGAGCTCTCAGAGGCGCAGATGCAGGAAGACTACTACCGCGCCGTGCGCACGTAC
ATCC 62541 (601)	GACCGGCTCGAGGTGGCCCCGCCCGAGCTCTCAGAGGCGCAGATGCAGGAAGACTACTACCGCGCCGTGCGCACGTAC
ATCC 66136 (637)	GACCGGCTCGAGGTGGCCCCGCCCGAGCTCTCAGAGGCGCAGATGCAGGAAGATTACTACCGCGCCGTGCGCACGTAC
FR00742 2009	GACCGGCTCGAGGTGGCCCCGCCCGAGCTCTCAGAGGCGCAGATGCAGGAAGATTACTACCGCGCCGTGCGCACGTAC
TB00024 2005	GACCGGCTCGAGGTGGCCCCGCCCGAGCTCTCAGAGGCGCAGATGCAGGAAGACTACTACCGCGCCGTGCGCACGTAC
UAMH 5584	GACCGGCTCGAGGTGGCCCCGCCCGAGCTCTCAGAGGCGCAGATGCAGGAAGACTACTACCGCGCCGTGCGCACGTAC
371	GACCGGCTCGAGGTGGCCCCGCCCGAGCTCTCAGAGGCGCAGATGCAGGAAGACTACTACCGCGCCGTGCGCACGTAC
396	GACCGGCTCGAGGTGGCCCCGCCCGAGCTCTCAGAGGCGCAGATGCAGGAAGACTACTACCGCGCCGTGCGCACGTAC
397	GACCGGCTCGAGGTGGCCCCGCCCGAGCTCTCAGAGGCGCAGATGCAGGAAGACTACTACCGCGCCGTGCGCACGTAC
663	GACCGGCTCGAGGTGGCCCCGCCCGAGCTCTCAGAGGCGCAGATGCAGGAAGACTACTACCGCGCCGTGCGCACGTAC
664	GACCGGCTCGAGGTGGCCCCGCCCGAGCTCTCAGAGGCGCAGATGCAGGAAGACTACTACCGCGCCGTGCGCACGTAC
ATCC 26199 (V)	GACCGGCTCGAGGTGGCCCCGCCCGAGCTCTCAGAGGCGCAGATGCAGGAAGACTACTACCGCGCCGTGCGCACGTAC
ATCC MYA-2586 (ER-3)	GACCGGCTCGAGGTGGCCCCGCCCGAGCTCTCAGAGGCGCAGATGCAGGAAGACTACTACCGCGCCGTGCGCACGTAC
B3003 (UAMH 12046)	GACCGGCTCGAGGTGGCCCCGCCCGAGCTCTCAGAGGCGCAGATGCAGGAAGACTACTACCGCGCCGTGCGCACGTAC
CH-10	GACCGGCTCGAGGTGGCCCCGCCCGAGCTCTCAGAGGCGCAGATGCAGGAAGACTACTACCGCGCCGTGCGCACGTAC
En	GACCGGCTCGAGGTGGCCCCGCCCGAGCTCTCAGAGGCGCAGATGCAGGAAGACTACTACCGCGCCGTGCGCACGTAC
F270	GACCGGCTCGAGGTGGCCCCGCCCGAGCTCTCAGAGGCGCAGATGCAGGAAGACTACTACCGCGCCGTGCGCACGTAC
FR00346 2009	GACCGGCTCGAGGTGGCCCCGCCCGAGCTCTCAGAGGCGCAGATGCAGGAAGACTACTACCGCGCCGTGCGCACGTAC
K940	GACCGGCTCGAGGTGGCCCCGCCCGAGCTCTCAGAGGCGCAGATGCAGGAAGACTACTACCGCGCCGTGCGCACGTAC
Ro	GACCGGCTCGAGGTGGCCCCGCCCGAGCTCTCAGAGGCGCAGATGCAGGAAGACTACTACCGCGCCGTGCGCACGTAC
SF03443 2009	GACCGGCTCGAGGTGGCCCCGCCCGAGCTCTCAGAGGCGCAGATGCAGGAAGACTACTACCGCGCCGTGCGCACGTAC
SF05773 2009	GACCGGCTCGAGGTGGCCCCGCCCGAGCTCTCAGAGGCGCAGATGCAGGAAGACTACTACCGCGCCGTGCGCACGTAC
SF05792 2009	GACCGGCTCGAGGTGGCCCCGCCCGAGCTCTCAGAGGCGCAGATGCAGGAAGACTACTACCGCGCCGTGCGCACGTAC
SF06072 2009	GACCGGCTCGAGGTGGCCCCGCCCGAGCTCTCAGAGGCGCAGATGCAGGAAGACTACTACCGCGCCGTGCGCACGTAC
SF06266 2009	GACCGGCTCGAGGTGGCCCCGCCCGAGCTCTCAGAGGCGCAGATGCAGGAAGACTACTACCGCGCCGTGCGCACGTAC
SF06354 2009	GACCGGCTCGAGGTGGCCCCGCCCGAGCTCTCAGAGGCGCAGATGCAGGAAGACTACTACCGCGCCGTGCGCACGTAC
SF14600 2008	GACCGGCTCGAGGTGGCCCCGCCCGAGCTCTCAGAGGCGCAGATGCAGGAAGACTACTACCGCGCCGTGCGCACGTAC
TB00011 2006	GACCGGCTCGAGGTGGCCCCGCCCGAGCTCTCAGAGGCGCAGATGCAGGAAGACTACTACCGCGCCGTGCGCACGTAC
TB00042 2005	GACCGGCTCGAGGTGGCCCCGCCCGAGCTCTCAGAGGCGCAGATGCAGGAAGACTACTACCGCGCCGTGCGCACGTAC
UAMH 4042	GACCGGCTCGAGGTGGCCCCGCCCGAGCTCTCAGAGGCGCAGATGCAGGAAGACTACTACCGCGCCGTGCGCACGTAC
UAMH 5634	GACCGGCTCGAGGTGGCCCCGCCCGAGCTCTCAGAGGCGCAGATGCAGGAAGACTACTACCGCGCCGTGCGCACGTAC
UAMH 7800	GACCGGCTCGAGGTGGCCCCGCCCGAGCTCTCAGAGGCGCAGATGCAGGAAGACTACTACCGCGCCGTGCGCACGTAC
ATCC 26197 (GA-1)	GACCGGCTCGAGGTGGCCCCGCCCGAGCTCTCAGAGGCGCAGATGCAGGAAGACTACTACCGCGCCGTGCGCACGTAC
ATCC 28306	GACCGGCTCGAGGTGGCCCCGCCCGAGCTCTCAGAGGCGCAGATGCAGGAAGACTACTACCGCGCCGTGCGCACGTAC

Figure S1: Nucleotide Sequence Alignments (continued)

drk1

EXON

TB00011 2006	TAGCTTCCACTTCACGTGCGTTGTAGAACTGGCAGAGCAGTCTATCAGCATGATTAGCGCGTCCCTCCTGCCCTACAAAAACCACCGTGTTCTGTTCATCGACAAAGGCCAGACCGGTGGCCACGCAGAGGAAATTACTGAAATGCTGAAGCAGCTTGACCTAGAGCCCATTGTTGTGAAGGATGAATCGCAGGTACCACCACCGGAAATTCAGGATCCCACAGGCAAGGATTCTGGACATGC
TB00024 2005	TAGCTTCCACTTCACGTGCGTTGTAGAACTGGCAGAGCAGTCTATCAGCATGATTAGCGCGTCCCTCCTGCCCTACAAAAACCACCGTGTTCTGTTCATCGACAAAGGCCAGACCGGTGGCCACGCAGAGGAAATTACTGAAATGCTGAAGCAGCTTGACCTAGAGCCCATTGTTGTGAAGGATGAATCGCAGGTACCACCACCGGAAATTCAGGATCCCACAGGCAAGGATTCTGGACATGC
TB00042 2005	TAGCTTCCACTTCACGTGCGTTGTAGAACTGGCAGAGCAGTCTATCAGCATGATTAGCGCGTCCCTCCTGCCCTACAAAAACCACCGTGTTCTGTTCATCGACAAAGGCCAGACCGGTGGCCACGCAGAGGAAATTACTGAAATGCTGAAGCAGCTTGACCTAGAGCCCATTGTTGTGAAGGATGAATCGCAGGTACCACCACCGGAAATTCAGGATCCCACAGGCAAGGATTCTGGACATGC
UAMH 4042	TAGCTTCCACTTCACGTGCGTTGTAGAACTGGCAGAGCAGTCTATCAGCATGATTAGCGCGTCCCTCCTGCCCTACAAAAACCACCGTGTTCTGTTCATCGACAAAGGCCAGACCGGTGGCCACGCAGAGGAAATTACTGAAATGCTGAAGCAGCTTGACCTAGAGCCCATTGTTGTGAAGGATGAATCGCAGGTACCACCACCGGAAATTCAGGATCCCACAGGCAAGGATTCTGGACATGC
UAMH 5584	TAGCTTCCACTTCACGTGCGTTGTAGAACTGGCAGAGCAGTCTATCAGCATGATTAGCGCGTCCCTCCTGCCCTACAAAAACCACCGTGTTCTGTTCATCGACAAAGGCCAGACCGGTGGCCACGCAGAGGAAATTACTGAAATGCTGAAGCAGCTTGACCTAGAGCCCATTGTTGTGAAGGATGAATCGCAGGTACCACCACCGGAAATTCAGGATCCCACAGGCAAGGATTCTGGACATGC
UAMH 5634	TAGCTTCCACTTCACGTGCGTTGTAGAACTGGCAGAGCAGTCTATCAGCATGATTAGCGCGTCCCTCCTGCCCTACAAAAACCACCGTGTTCTGTTCATCGACAAAGGCCAGACCGGTGGCCACGCAGAGGAAATTACTGAAATGCTGAAGCAGCTTGACCTAGAGCCCATTGTTGTGAAGGATGAATCGCAGGTACCACCACCGGAAATTCAGGATCCCACAGGCAAGGATTCTGGACATGC
UAMH 7800	TAGCTTCCACTTCACGTGCGTTGTAGAACTGGCAGAGCAGTCTATCAGCATGATTAGCGCGTCCCTCCTGCCCTACAAAAACCACCGTGTTCTGTTCATCGACAAAGGCCAGACCGGTGGCCACGCAGAGGAAATTACTGAAATGCTGAAGCAGCTTGACCTAGAGCCCATTGTTGTGAAGGATGAATCGCAGGTACCACCACCGGAAATTCAGGATCCCACAGGCAAGGATTCTGGACATGC
664	TAGCTTCCACTTCACGTGCGTTGTAGAACTGGCAGACCAGTCTATCAGCATGATTAGCGCGTCCCTCCTGCCCTACAAAAACCACCGTGTTCTGTTCATCGACAAAGGCCAGACCGGTGGCCACGCAGAGGAAATTACTGAAATGCTGAAGCAGCTTGACCTAGAGCCCATTGTTGTGAAGGATGAATCGCAGGTACCACCACCGGAAATTCAGGATCCCACAGGCAAGGATTCTGGACATGC
B3003 (UAMH 12046)	TAGCTTCCACTTCACGTGCGTTGTAGAACTGGCAGACCAGTCTATCAGCATGATTAGCGCGTCCCTCCTGCCCTACAAAAACCACCGTGTTCTGTTCATCGACAAAGGCCAGACCGGTGGCCACGCAGAGGAAATTACTGAAATGCTGAAGCAGCTTGACCTAGAGCCCATTGTTGTGAAGGATGAATCGCAGGTACCACCACCGGAAATTCAGGATCCCACAGGCAAGGATTCTGGACATGC
CH-10	TAGCTTCCACTTCACGTGCGTTGTAGAACTGGCAGACCAGTCTATCAGCATGATTAGCGCGTCCCTCCTGCCCTACAAAAACCACCGTGTTCTGTTCATCGACAAAGGCCAGACCGGTGGCCACGCAGAGGAAATTACTGAAATGCTGAAGCAGCTTGACCTAGAGCCCATTGTTGTGAAGGATGAATCGCAGGTACCACCACCGGAAATTCAGGATCCCACAGGCAAGGATTCTGGACATGC
F270	TAGCTTCCACTTCACGTGCGTTGTAGAACTGGCAGACCAGTCTATCAGCATGATTAGCGCGTCCCTCCTGCCCTACAAAAACCACCGTGTTCTGTTCATCGACAAAGGCCAGACCGGTGGCCACGCAGAGGAAATTACTGAAATGCTGAAGCAGCTTGACCTAGAGCCCATTGTTGTGAAGGATGAATCGCAGGTACCACCACCGGAAATTCAGGATCCCACAGGCAAGGATTCTGGACATGC
K940	TAGCTTCCACTTCACGTGCGTTGTAGAACTGGCAGACCAGTCTATCAGCATGATTAGCGCGTCCCTCCTGCCCTACAAAAACCACCGTGTTCTGTTCATCGACAAAGGCCAGACCGGTGGCCACGCAGAGGAAATTACTGAAATGCTGAAGCAGCTTGACCTAGAGCCCATTGTTGTGAAGGATGAATCGCAGGTACCACCACCGGAAATTCAGGATCCCACAGGCAAGGATTCTGGACATGC
Ro	TAGCTTCCACTTCACGTGCGTTGTAGAACTGGCAGACCAGTCTATCAGCATGATTAGCGCGTCCCTCCTGCCCTACAAAAACCACCGTGTTCTGTTCATCGACAAAGGCCAGACCGGTGGCCACGCAGAGGAAATTACTGAAATGCTGAAGCAGCTTGACCTAGAGCCCATTGTTGTGAAGGATGAATCGCAGGTACCACCACCGGAAATTCAGGATCCCACAGGCAAGGATTCTGGACATGC
396	TAGCTTCCACTTCACGTGCGTTGTAGAACTGGCAGACCAGTCTATCAGCATGATTAGCGCGTCCCTCCTGCCCTACAAAAACCACCGTGTTCTGTTCATCGACAAAGGCCAGACCGGTGGCCACGCAGAGGAAATTACTGAAATGCTGAAGCAGCTTGACCTAGAGCCCATTGTTGTGAAGGATGAATCGCAGGTACCACCACCGGAAATTCAGGATCCCACAGGCAAGGATTCTGGACATGC
397	TAGCTTCCACTTCACGTGCGTTGTAGAACTGGCAGACCAGTCTATCAGCATGATTAGCGCGTCCCTCCTGCCCTACAAAAACCACCGTGTTCTGTTCATCGACAAAGGCCAGACCGGTGGCCACGCAGAGGAAATTACTGAAATGCTGAAGCAGCTTGACCTAGAGCCCATTGTTGTGAAGGATGAATCGCAGGTACCACCACCGGAAATTCAGGATCCCACAGGCAAGGATTCTGGACATGC
ATCC 28306	TAGCTTCCACTTCACGTGCGTTGTAGAACTGGCAGACCAGTCTATCAGCATGATTAGCGCGTCCCTCCTGCCCTACAAAAACCACCGTGTTCTGTTCATCGACAAAGGCCAGACCGGTGGCCACGCAGAGGAAATTACTGAAATGCTGAAGCAGCTTGACCTAGAGCCCATTGTTGTGAAGGATGAATCGCAGGTACCACCACCGGAAATTCAGGATCCCACAGGCAAGGATTCTGGACATGC
En	TAGCTTCCACTTCACGTGCGTTGTAGAACTGGCAGACCAGTCTATCAGCATGATTAGCGCGTCCCTCATGCCCTACAAAAACCACCGTGTTCTGTTCATCGACAAAGGCCAGACCGGTGGCCACGCAGAGGAAATTACTGAAATGCTGAAGCAGCTTGACCTAGAGCCCATTGTTGTGAAGGATGAATCGCAGGTACCACCACCGGAAATTCAGGATCCCACAGGCAAGGATTCTGGACATGC
371	TAGCTTCCACTTCACGTGCGTTGTAGAACTGGCAGACCAGTCTATCAGCATGATTAGCGCGTCCCTCATGCCCTACAAAAACCACCGTGTTCTGTTCATCGACAAAGGCCAGACCGGTGGCCACGCAGAGGAAATTACTGAAATGCTGAAGCAGCTTGACCTAGAGCCCATTGTTGTGAAGGATGAATCGCAGGTACCACCACCGGAAATTCAGGATCCCACAGGCAAGGATTCTGGACATGC
663	TAGCTTCCACTTCACGTGCGTTGTAGAACTGGCAGACCAGTCTATCAGCATGATTAGCGCGTCCCTCATGCCCTACAAAAACCACCGTGTTCTGTTCATCGACAAAGGCCAGACCGGTGGCCACGCAGAGGAAATTACTGAAATGCTGAAGCAGCTTGACCTAGAGCCCATTGTTGTGAAGGATGAATCGCAGGTACCACCACCGGAAATTCAGGATCCCACAGGCAAGGATTCTGGACATGC
ATCC 26197 (GA-1)	TAGCTTCCACTTCACGTGCGTTGTAGAACTGGCAGACCAGTCTATCAGCATGATTAGCGCGTCCCTCATGCCCTACAAAAACCACCGTGTTCTGTTCATCGACAAAGGCCAGACCGGTGGCCACGCAGAGGAAATTACTGAAATGCTGAAGCAGCTTGACCTAGAGCCCATTGTTGTGAAGGATGAATCGCAGGTACCACCACCGGAAATTCAGGATCCCACAGGCAAGGATTCTGGACATGC
ATCC 26199 (V)	TAGCTTCCACTTCACGTGCGTTGTAGAACTGGCAGACCAGTCTATCAGCATGATTAGCGCGTCCCTCATGCCCTACAAAAACCACCGTGTTCTGTTCATCGACAAAGGCCAGACCGGTGGCCACGCAGAGGAAATTACTGAAATGCTGAAGCAGCTTGACCTAGAGCCCATTGTTGTGAAGGATGAATCGCAGGTACCACCACCGGAAATTCAGGATCCCACAGGCAAGGATTCTGGACATGC
ATCC MYA-2586 (ER-3)	TAGCTTCCACTTCACGTGCGTTGTAGAACTGGCAGACCAGTCTATCAGCATGATTAGCGCGTCCCTCATGCCCTACAAAAACCACCGTGTTCTGTTCATCGACAAAGGCCAGACCGGTGGCCACGCAGAGGAAATTACTGAAATGCTGAAGCAGCTTGACCTAGAGCCCATTGTTGTGAAGGATGAATCGCAGGTACCACCACCGGAAATTCAGGATCCCACAGGCAAGGATTCTGGACATGC
FR00346 2009	TAGCTTCCACTTCACGTGCGTTGTAGAACTGGCAGACCAGTCTATCAGCATGATTAGCGCGTCCCTCATGCCCTACAAAAACCACCGTGTTCTGTTCATCGACAAAGGCCAGACCGGTGGCCACGCAGAGGAAATTACTGAAATGCTGAAGCAGCTTGACCTAGAGCCCATTGTTGTGAAGGATGAATCGCAGGTACCACCACCGGAAATTCAGGATCCCACAGGCAAGGATTCTGGACATGC
SF03443 2009	TAGCTTCCACTTCACGTGCGTTGTAGAACTGGCAGACCAGTCTATCAGCATGATTAGCGCGTCCCTCATGCCCTACAAAAACCACCGTGTTCTGTTCATCGACAAAGGCCAGACCGGTGGCCACGCAGAGGAAATTACTGAAATGCTGAAGCAGCTTGACCTAGAGCCCATTGTTGTGAAGGATGAATCGCAGGTACCACCACCGGAAATTCAGGATCCCACAGGCAAGGATTCTGGACATGC
SF05773 2009	TAGCTTCCACTTCACGTGCGTTGTAGAACTGGCAGACCAGTCTATCAGCATGATTAGCGCGTCCCTCATGCCCTACAAAAACCACCGTGTTCTGTTCATCGACAAAGGCCAGACCGGTGGCCACGCAGAGGAAATTACTGAAATGCTGAAGCAGCTTGACCTAGAGCCCATTGTTGTGAAGGATGAATCGCAGGTACCACCACCGGAAATTCAGGATCCCACAGGCAAGGATTCTGGACATGC
SF05792 2009	TAGCTTCCACTTCACGTGCGTTGTAGAACTGGCAGACCAGTCTATCAGCATGATTAGCGCGTCCCTCATGCCCTACAAAAACCACCGTGTTCTGTTCATCGACAAAGGCCAGACCGGTGGCCACGCAGAGGAAATTACTGAAATGCTGAAGCAGCTTGACCTAGAGCCCATTGTTGTGAAGGATGAATCGCAGGTACCACCACCGGAAATTCAGGATCCCACAGGCAAGGATTCTGGACATGC
SF06072 2009	TAGCTTCCACTTCACGTGCGTTGTAGAACTGGCAGACCAGTCTATCAGCATGATTAGCGCGTCCCTCATGCCCTACAAAAACCACCGTGTTCTGTTCATCGACAAAGGCCAGACCGGTGGCCACGCAGAGGAAATTACTGAAATGCTGAAGCAGCTTGACCTAGAGCCCATTGTTGTGAAGGATGAATCGCAGGTACCACCACCGGAAATTCAGGATCCCACAGGCAAGGATTCTGGACATGC
SF06266 2009	TAGCTTCCACTTCACGTGCGTTGTAGAACTGGCAGACCAGTCTATCAGCATGATTAGCGCGTCCCTCATGCCCTACAAAAACCACCGTGTTCTGTTCATCGACAAAGGCCAGACCGGTGGCCACGCAGAGGAAATTACTGAAATGCTGAAGCAGCTTGACCTAGAGCCCATTGTTGTGAAGGATGAATCGCAGGTACCACCACCGGAAATTCAGGATCCCACAGGCAAGGATTCTGGACATGC
SF06354 2009	TAGCTTCCACTTCACGTGCGTTGTAGAACTGGCAGACCAGTCTATCAGCATGATTAGCGCGTCCCTCATGCCCTACAAAAACCACCGTGTTCTGTTCATCGACAAAGGCCAGACCGGTGGCCACGCAGAGGAAATTACTGAAATGCTGAAGCAGCTTGACCTAGAGCCCATTGTTGTGAAGGATGAATCGCAGGTACCACCACCGGAAATTCAGGATCCCACAGGCAAGGATTCTGGACATGC
SF14600 2008	TAGCTTCCACTTCACGTGCGTTGTAGAACTGGCAGACCAGTCTATCAGCATGATTAGCGCGTCCCTCATGCCCTACAAAAACCACCGTGTTCTGTTCATCGACAAAGGCCAGACCGGTGGCCACGCAGAGGAAATTACTGAAATGCTGAAGCAGCTTGACCTAGAGCCCATTGTTGTGAAGGATGAATCGCAGGTACCACCACCGGAAATTCAGGATCCCACAGGCAAGGATTCTGGACATGC
B1566 (UAMH 12045)	TAGCTTCCACTTCACGTGCGTTGTAGAACTGGCAGACCAGTCTATCAGCATGATTAGCGCGTCCCTCCTGCCCTACAAAAACCACCGTGTTCTGTTCATCGACAAAGGCCAGACCGGTGGCCACGCAGAGGAAATTACCGAAATGCTGAAGCAGCTTGACCTAGAGCCCATTGTTGTGAAGGATGAATCGCAGGTACCACCACCGGAAATTCAGGATCCCACAGGCAAGGATTCCGGACATGC
ATCC 62541 (601)	TAGCTTCCACTTCACGTGCGTTGTAGAACTGGCAGACCAGTCTATCAGCATGATTAGCGCGTCCCTCCTGCCCTACAAAAACCACCGTGTTCTGTTCATCGACAAAGGCCAGACCGGTGGCCACGCAGAGGAAATTACCGAAATGCTGAAGCAGCTTGACCTAGAGCCCATTGTTGTGAAGGATGAATCGCAGGTACCACCACCGGAAATTCAGGATCCCACAGGCAAGGATTCCGGACATGC
ATCC 66136 (637)	TAGCTTCCACTTCACGTGCGTTGTAGAACTGGCAGACCAGTCTATCAGCATGATTAGCGCGTCCCTCCTGCCCTACAAAAACCACCGTGTTCTGTTCATCGACAAAGGCCAGACCGGTGGCCACGCAGAGGAAATTACCGAAATGCTGAAGCAGCTTGACCTAGAGCCCATTGTTGTGAAGGATGAATCGCAGGTACCACCACCGGAAATTCAGGATCCCACAGGCAAGGATTCCGGACATGC
FR00742 2009	TAGCTTCCACTTCACGTGCGTTGTAGAACTGGCAGACCAGTCTATCAGCATGATTAGCGCGTCCCTCCTGCCCTACAAAAACCACCGTGTTCTGTTCATCGACAAAGGCCAGACCGGTGGCCACGCAGAGGAAATTACCGAAATGCTGAAGCAGCTTGACCTAGAGCCCATTGTTGTGAAGGATGAATCGCAGGTACCACCACCGGAAATTCAGGATCCCACAGGCAAGGATTCCGGACATGC
TB00016 2005	TAGCTTCCACTTCACGTGCGTTGTAGAACTGGCAGACCAGTCTATCAGCATGATTAGCGCGTCCCTCCTGCCCTACAAAAACCACCGTGTTCTGTTCATCGACAAAGGCCAGACCGGTGGCCACGCAGAGGAAATTACCGAAATGCTGAAGCAGCTTGACCTAGAGCCCATTGTTGTGAAGGATGAATCGCAGGTACCACCACCGGAAATTCAGGATCCCACAGGCAAGGATTCCGGACATGC
TB00040 2005	TAGCTTCCACTTCACGTGCGTTGTAGAACTGGCAGACCAGTCTATCAGCATGATTAGCGCGTCCCTCCTGCCCTACAAAAACCACCGTGTTCTGTTCATCGACAAAGGCCAGACCGGTGGCCACGCAGAGGAAATTACCGAAATGCTGAAGCAGCTTGACCTAGAGCCCATTGTTGTGAAGGATGAATCGCAGGTACCACCACCGGAAATTCAGGATCCCACAGGCAAGGATTCCGGACATGC

drk1(continued)

EXON

TB00011 2006	TTACGACGTTATAATCGTCGACTCCGTCGACACCGCGCGAAATCTGCGCACGTACGATGAGTTTAAATACATACCTATCGTTCTATTATGCCCTGTCGTTTCTGTCAGTCTGAAGTCAGCACTGGATCTGGGTATCACATCCTACATGACGACTCCTTGTCAGCCTATCGATCTAGGCAATGGCATGTTGCCTGCTCTTGAAGGACGTTCCACGCCAATAACTACGGACCACACAAGATCGTTTGA
TB00024 2005	TTACGACGTTATAATCGTCGACTCCGTCGACACCGCGCGAAATCTGCGCACGTACGATGAGTTTAAATACATACCTATCGTTCTATTATGCCCTGTCGTTTCTGTCAGTCTGAAGTCAGCACTGGATCTGGGTATCACATCCTACATGACGACTCCTTGTCAGCCTATCGATCTAGGCAATGGCATGTTGCCTGCTCTTGAAGGACGTTCCACGCCAATAACTACGGACCACACAAGATCGTTTGA
TB00042 2005	TTACGACGTTATAATCGTCGACTCCGTCGACACCGCGCGAAATCTGCGCACGTACGATGAGTTTAAATACATACCTATCGTTCTATTATGCCCTGTCGTTTCTGTCAGTCTGAAGTCAGCACTGGATCTGGGTATCACATCCTACATGACGACTCCTTGTCAGCCTATCGATCTAGGCAATGGCATGTTGCCTGCTCTTGAAGGACGTTCCACGCCAATAACTACGGACCACACAAGATCGTTTGA
UAMH 4042	TTACGACGTTATAATCGTCGACTCCGTCGACACCGCGCGAAATCTGCGCACGTACGATGAGTTTAAATACATACCTATCGTTCTATTATGCCCTGTCGTTTCTGTCAGTCTGAAGTCAGCACTGGATCTGGGTATCACATCCTACATGACGACTCCTTGTCAGCCTATCGATCTAGGCAATGGCATGTTGCCTGCTCTTGAAGGACGTTCCACGCCAATAACTACGGACCACACAAGATCGTTTGA
UAMH 5584	TTACGACGTTATAATCGTCGACTCCGTCGACACCGCGCGAAATCTGCGCACGTACGATGAGTTTAAATACATACCTATCGTTCTATTATGCCCTGTCGTTTCTGTCAGTCTGAAGTCAGCACTGGATCTGGGTATCACATCCTACATGACGACTCCTTGTCAGCCTATCGATCTAGGCAATGGCATGTTGCCTGCTCTTGAAGGACGTTCCACGCCAATAACTACGGACCACACAAGATCGTTTGA
UAMH 5634	TTACGACGTTATAATCGTCGACTCCGTCGACACCGCGCGAAATCTGCGCACGTACGATGAGTTTAAATACATACCTATCGTTCTATTATGCCCTGTCGTTTCTGTCAGTCTGAAGTCAGCACTGGATCTGGGTATCACATCCTACATGACGACTCCTTGTCAGCCTATCGATCTAGGCAATGGCATGTTGCCTGCTCTTGAAGGACGTTCCACGCCAATAACTACGGACCACACAAGATCGTTTGA
UAMH 7800	TTACGACGTTATAATCGTCGACTCCGTCGACACCGCGCGAAATCTGCGCACGTACGATGAGTTTAAATACATACCTATCGTTCTATTATGCCCTGTCGTTTCTGTCAGTCTGAAGTCAGCACTGGATCTGGGTATCACATCCTACATGACGACTCCTTGTCAGCCTATCGATCTAGGCAATGGCATGTTGCCTGCTCTTGAAGGACGTTCCACGCCAATAACTACGGACCACACAAGATCGTTTGA
664	TTACGACGTTATAATCGTCGACTCCGTCGACACCGCGCGAAATCTGCGCACGTACGATGAGTTTAAATACATACCTATCGTTCTATTATGCCCTGTCGTTTCTGTCAGTCTGAAGTCAGCACTGGATCTGGGTATCACATCCTACATGACGACTCCTTGTCAGCCTATCGATCTAGGCAATGGCATGTTGCCTGCTCTTGAAGGACGTTCCACGCCAATAACTACGGACCACACAAGATCGTTTGA
B3003 (UAMH 12046)	TTACGACGTTATAATCGTCGACTCCGTCGACACCGCGCGAAATCTGCGCACGTACGATGAGTTTAAATACATACCTATCGTTCTATTATGCCCTGTCGTTTCTGTCAGTCTGAAGTCAGCACTGGATCTGGGTATCACATCCTACATGACGACTCCTTGTCAGCCTATCGATCTAGGCAATGGCATGTTGCCTGCTCTTGAAGGACGTTCCACGCCAATAACTACGGACCACACAAGATCGTTTGA
CH-10	TTACGACGTTATAATCGTCGACTCCGTCGACACCGCGCGAAATCTGCGCACGTACGATGAGTTTAAATACATACCTATCGTTCTATTATGCCCTGTCGTTTCTGTCAGTCTGAAGTCAGCACTGGATCTGGGTATCACATCCTACATGACGACTCCTTGTCAGCCTATCGATCTAGGCAATGGCATGTTGCCTGCTCTTGAAGGACGTTCCACGCCAATAACTACGGACCACACAAGATCGTTTGA
F270	TTACGACGTTATAATCGTCGACTCCGTCGACACCGCGCGAAATCTGCGCACGTACGATGAGTTTAAATACATACCTATCGTTCTATTATGCCCTGTCGTTTCTGTCAGTCTGAAGTCAGCACTGGATCTGGGTATCACATCCTACATGACGACTCCTTGTCAGCCTATCGATCTAGGCAATGGCATGTTGCCTGCTCTTGAAGGACGTTCCACGCCAATAACTACGGACCACACAAGATCGTTTGA
K940	TTACGACGTTATAATCGTCGACTCCGTCGACACCGCGCGAAATCTGCGCACGTACGATGAGTTTAAATACATACCTATCGTTCTATTATGCCCTGTCGTTTCTGTCAGTCTGAAGTCAGCACTGGATCTGGGTATCACATCCTACATGACGACTCCTTGTCAGCCTATCGATCTAGGCAATGGCATGTTGCCTGCTCTTGAAGGACGTTCCACGCCAATAACTACGGACCACACAAGATCGTTTGA
Ro	TTACGACGTTATAATCGTCGACTCCGTCGACACCGCGCGAAATCTGCGCACGTACGATGAGTTTAAATACATACCTATCGTTCTATTATGCCCTGTCGTTTCTGTCAGTCTGAAGTCAGCACTGGATCTGGGTATCACATCCTACATGACGACTCCTTGTCAGCCTATCGATCTAGGCAATGGCATGTTGCCTGCTCTTGAAGGACGTTCCACGCCAATAACTACGGACCACACAAGATCGTTTGA
396	TTACGACGTTATAATCGTCGACTCCGTGGACACCGCGCGAAATCTGCGCACGTACGATGAGTTTAAATACATACCTATCGTTCTATTATGCCCTGTCGTTTCTGTCAGTCTGAAGTCAGCACTGGATCTGGGTATCACATCCTACATGACGACTCCTTGTCAGCCTATCGATCTAGGCAATGGCATGTTGCCTGCTCTTGAAGGACGTTCCACGCCAATAACTACGGACCACACAAGATCGTTTGA
397	TTACGACGTTATAATCGTCGACTCCGTGGACACCGCGCGAAATCTGCGCACGTACGATGAGTTTAAATACATACCTATCGTTCTATTATGCCCTGTCGTTTCTGTCAGTCTGAAGTCAGCACTGGATCTGGGTATCACATCCTACATGACGACTCCTTGTCAGCCTATCGATCTAGGCAATGGCATGTTGCCTGCTCTTGAAGGACGTTCCACGCCAATAACTACGGACCACACAAGATCGTTTGA
ATCC 28306	TTACGACGTTATAATCGTCGACTCCGTGGACACCGCGCGAAATCTGCGCACGTACGATGAGTTTAAATACATACCTATCGTTCTATTATGCCCTGTCGTTTCTGTCAGTCTGAAGTCAGCACTGGATCTGGGTATCACATCCTACATGACGACTCCTTGTCAGCCTATCGATCTAGGCAATGGCATGTTGCCTGCTCTTGAAGGACGTTCCACGCCAATAACTACGGACCACACAAGATCGTTTGA
En	TTACGACGTTATAATCGTCGACTCCGTCGACACCGCGCGAAATCTGCGCACGTACGATGAGTTTAAATACATACCTATCGTTCTATTATGCCCTGTCGTTTCTGTCAGTCTGAAGTCAGCACTGGATCTGGGTATCACATCCTACATGACGACTCCTTGTCAGCCTATCGATCTAGGCAATGGCATGTTGCCTGCTCTTGAAGGACGTTCCACGCCAATAACTACGGACCACACAAGATCGTTTGA
371	TTACGACGTTATAATCGTCGACTCCGTCGACACCGCGCGAAATCTGCGCACGTACGATGAGTTTAAATACATACCTATCGTTCTATTATGCCCTGTCGTTTCTGTCAGTCTGAAGTCAGCACTGGATCTGGGTATCACATCGTACATGACAACTCCTTGTCAGCCTATCGATCTAGGCAATGGCATGTTGCCTGCTCTTGAAGGACGTTCCACGCCAATAACTACGGACCACACAAGATCGTTTGA
663	TTACGACGTTATAATCGTCGACTCCGTCGACACCGCGCGAAATCTGCGCACGTACGATGAGTTTAAATACATACCTATCGTTCTATTATGCCCTGTCGTTTCTGTCAGTCTGAAGTCAGCACTGGATCTGGGTATCACATCGTACATGACAACTCCTTGTCAGCCTATCGATCTAGGCAATGGCATGTTGCCTGCTCTTGAAGGACGTTCCACGCCAATAACTACGGACCACACAAGATCGTTTGA
ATCC 26197 (GA-1)	TTACGACGTTATAATCGTCGACTCCGTCGACACCGCGCGAAATCTGCGCACGTACGATGAGTTTAAATACATACCTATCGTTCTATTATGCCCTGTCGTTTCTGTCAGTCTGAAGTCAGCACTGGATCTGGGTATCACATCGTACATGACAACTCCTTGTCAGCCTATCGATCTAGGCAATGGCATGTTGCCTGCTCTTGAAGGACGTTCCACGCCAATAACTACGGACCACACAAGATCGTTTGA
ATCC 26199 (V)	TTACGACGTTATAATCGTCGACTCCGTCGACACCGCGCGAAATCTGCGCACGTACGATGAGTTTAAATACATACCTATCGTTCTATTATGCCCTGTCGTTTCTGTCAGTCTGAAGTCAGCACTGGATCTGGGTATCACATCGTACATGACAACTCCTTGTCAGCCTATCGATCTAGGCAATGGCATGTTGCCTGCTCTTGAAGGACGTTCCACGCCAATAACTACGGACCACACAAGATCGTTTGA
ATCC MYA-2586 (ER-3)	TTACGACGTTATAATCGTCGACTCCGTCGACACCGCGCGAAATCTGCGCACGTACGATGAGTTTAAATACATACCTATCGTTCTATTATGCCCTGTCGTTTCTGTCAGTCTGAAGTCAGCACTGGATCTGGGTATCACATCGTACATGACAACTCCTTGTCAGCCTATCGATCTAGGCAATGGCATGTTGCCTGCTCTTGAAGGACGTTCCACGCCAATAACTACGGACCACACAAGATCGTTTGA
FR00346 2009	TTACGACGTTATAATCGTCGACTCCGTCGACACCGCGCGAAATCTGCGCACGTACGATGAGTTTAAATACATACCTATCGTTCTATTATGCCCTGTCGTTTCTGTCAGTCTGAAGTCAGCACTGGATCTGGGTATCACATCGTACATGACAACTCCTTGTCAGCCTATCGATCTAGGCAATGGCATGTTGCCTGCTCTTGAAGGACGTTCCACGCCAATAACTACGGACCACACAAGATCGTTTGA
SF03443 2009	TTACGACGTTATAATCGTCGACTCCGTCGACACCGCGCGAAATCTGCGCACGTACGATGAGTTTAAATACATACCTATCGTTCTATTATGCCCTGTCGTTTCTGTCAGTCTGAAGTCAGCACTGGATCTGGGTATCACATCGTACATGACAACTCCTTGTCAGCCTATCGATCTAGGCAATGGCATGTTGCCTGCTCTTGAAGGACGTTCCACGCCAATAACTACGGACCACACAAGATCGTTTGA
SF05773 2009	TTACGACGTTATAATCGTCGACTCCGTCGACACCGCGCGAAATCTGCGCACGTACGATGAGTTTAAATACATACCTATCGTTCTATTATGCCCTGTCGTTTCTGTCAGTCTGAAGTCAGCACTGGATCTGGGTATCACATCGTACATGACAACTCCTTGTCAGCCTATCGATCTAGGCAATGGCATGTTGCCTGCTCTTGAAGGACGTTCCACGCCAATAACTACGGACCACACAAGATCGTTTGA
SF05792 2009	TTACGACGTTATAATCGTCGACTCCGTCGACACCGCGCGAAATCTGCGCACGTACGATGAGTTTAAATACATACCTATCGTTCTATTATGCCCTGTCGTTTCTGTCAGTCTGAAGTCAGCACTGGATCTGGGTATCACATCGTACATGACAACTCCTTGTCAGCCTATCGATCTAGGCAATGGCATGTTGCCTGCTCTTGAAGGACGTTCCACGCCAATAACTACGGACCACACAAGATCGTTTGA
SF06072 2009	TTACGACGTTATAATCGTCGACTCCGTCGACACCGCGCGAAATCTGCGCACGTACGATGAGTTTAAATACATACCTATCGTTCTATTATGCCCTGTCGTTTCTGTCAGTCTGAAGTCAGCACTGGATCTGGGTATCACATCGTACATGACAACTCCTTGTCAGCCTATCGATCTAGGCAATGGCATGTTGCCTGCTCTTGAAGGACGTTCCACGCCAATAACTACGGACCACACAAGATCGTTTGA
SF06266 2009	TTACGACGTTATAATCGTCGACTCCGTCGACACCGCGCGAAATCTGCGCACGTACGATGAGTTTAAATACATACCTATCGTTCTATTATGCCCTGTCGTTTCTGTCAGTCTGAAGTCAGCACTGGATCTGGGTATCACATCGTACATGACAACTCCTTGTCAGCCTATCGATCTAGGCAATGGCATGTTGCCTGCTCTTGAAGGACGTTCCACGCCAATAACTACGGACCACACAAGATCGTTTGA
SF06354 2009	TTACGACGTTATAATCGTCGACTCCGTCGACACCGCGCGAAATCTGCGCACGTACGATGAGTTTAAATACATACCTATCGTTCTATTATGCCCTGTCGTTTCTGTCAGTCTGAAGTCAGCACTGGATCTGGGTATCACATCGTACATGACAACTCCTTGTCAGCCTATCGATCTAGGCAATGGCATGTTGCCTGCTCTTGAAGGACGTTCCACGCCAATAACTACGGACCACACAAGATCGTTTGA
SF14600 2008	TTACGACGTTATAATCGTCGACTCCGTCGACACCGCGCGAAATCTGCGCACGTACGATGAGTTTAAATACATACCTATCGTTCTATTATGCCCTGTCGTTTCTGTCAGTCTGAAGTCAGCACTGGATCTGGGTATCACATCGTACATGACAACTCCTTGTCAGCCTATCGATCTAGGCAATGGCATGTTGCCTGCTCTTGAAGGACGTTCCACGCCAATAACTACGGACCACACAAGATCGTTTGA
B1566 (UAMH 12045)	TTACGACGTTATAATCGTCGACTCCGTCGACACCGCGCGAAATCTGCGCACGTACGATGAGTTTAAATACATACCTATCGTTCTATTATGCCCTGTCGTTTCTGTCAGTCTGAAGTCAGCACTGGATCTGGGTATCACATCCTACATGACGACTCCTTGTCAGCCTATCGATCTAGGCAATGGCATGTTGCCTGCTCTTGAAGGACGTTCCACGCCAATAACTACAGACCACACAAGATCGTTTGA
ATCC 62541 (601)	TTACGACGTTATAATCGTCGACTCCGTCGACACCGCGCGAAATCTGCGCACGTACGATGAGTTTAAATACATACCTATCGTTCTATTATGCCCTGTCGTTTCTGTCAGTCTGAAGTCAGCACTGGATCTGGGTATCACATCCTACATGACGACTCCTTGTCAGCCTATCGATCTAGGCAATGGCATGTTGCCTGCTCTTGAAGGACGTTCCACGCCAATAACTACAGACCACACAAGATCGTTTGA
ATCC 66136 (637)	TTACGACGTTATAATCGTCGACTCCGTCGACACCGCGCGAAATCTGCGCACGTACGATGAGTTTAAATACATACCTATCGTTCTATTATGCCCTGTCGTTTCTGTCAGTCTGAAGTCAGCACTGGATCTGGGTATCACATCCTACATGACGACTCCTTGTCAGCCTATCGATCTAGGCAATGGCATGTTGCCTGCTCTTGAAGGACGTTCCACGCCAATAACTACAGACCACACAAGATCGTTTGA
FR00742 2009	TTACGACGTTATAATCGTCGACTCCGTCGACACCGCGCGAAATCTGCGCACGTACGATGAGTTTAAATACATACCTATCGTTCTATTATGCCCTGTCGTTTCTGTCAGTCTGAAGTCAGCACTGGATCTGGGTATCACATCCTACATGACGACTCCTTGTCAGCCTATCGATCTAGGCAATGGCATGTTGCCTGCTCTTGAAGGACGTTCCACGCCAATAACTACAGACCACACAAGATCGTTTGA
TB00016 2005	TTACGACGTTATAATCGTCGACTCCGTCGACACCGCGCGAAATCTGCGCACGTACGATGAGTTTAAATACATACCTATCGTTCTATTATGCCCTGTCGTTTCTGTCAGTCTGAAGTCAGCACTGGATCTGGGTATCACATCCTACATGACGACTCCTTGTCAGCCTATCGATCTAGGCAATGGCATGTTGCCTGCTCTTGAAGGACGTTCCACGCCAATAACTACAGACCACACAAGATCGTTTGA
TB00040 2005	TTACGACGTTATAATCGTCGACTCCGTCGACACCGCGCGAAATCTGCGCACGTACGATGAGTTTAAATACATACCTATCGTTCTATTATGCCCTGTCGTTTCTGTCAGTCTGAAGTCAGCACTGGATCTGGGTATCACATCCTACATGACGACTCCTTGTCAGCCTATCGATCTAGGCAATGGCATGTTGCCTGCTCTTGAAGGACGTTCCACGCCAATAACTACAGACCACACAAGATCGTTTGA

Figure S1: Nucleotide Sequence Alignments Alignments (continued)

drk1 (continued)

EXON

TB00011 2006	TATTCTTTTGGCCGAGGACAACGATGTGAACCAGAGGGTTGCCGTCAAGATACTGGAGAAATGCAACCATGGCGTGACCGTTGTCAGCAATGGCCTTCAAGCTGTGGAAGCGATCAAGAAGCGTCGATACGACGTCATTTTGATGGATGTCCAAATG
TB00024 2005	TATTCTTTTGGCCGAGGACAACGATGTGAACCAGAGGGTTGCCGTCAAGATACTGGAGAAATGCAACCATGGCGTGACCGTTGTCAGCAATGGCCTTCAAGCTGTGGAAGCGATCAAGAAGCGTCGATACGACGTCATTTTGATGGATGTCCAAATG
TB00042 2005	TATTCTTTTGGCCGAGGACAACGATGTGAACCAGAGGGTTGCCGTCAAGATACTGGAGAAATGCAACCATGGCGTGACCGTTGTCAGCAATGGCCTTCAAGCTGTGGAAGCGATCAAGAAGCGTCGATACGACGTCATTTTGATGGATGTCCAAATG
UAMH 4042	TATTCTTTTGGCCGAGGACAACGATGTGAACCAGAGGGTTGCCGTCAAGATACTGGAGAAATGCAACCATGGCGTGACCGTTGTCAGCAATGGCCTTCAAGCTGTGGAAGCGATCAAGAAGCGTCGATACGACGTCATTTTGATGGATGTCCAAATG
UAMH 5584	TATTCTTTTGGCCGAGGACAACGATGTGAACCAGAGGGTTGCCGTCAAGATACTGGAGAAATGCAACCATGGCGTGACCGTTGTCAGCAATGGCCTTCAAGCTGTGGAAGCGATCAAGAAGCGTCGATACGACGTCATTTTGATGGATGTCCAAATG
UAMH 5634	TATTCTTTTGGCCGAGGACAACGATGTGAACCAGAGGGTTGCCGTCAAGATACTGGAGAAATGCAACCATGGCGTGACCGTTGTCAGCAATGGCCTTCAAGCTGTGGAAGCGATCAAGAAGCGTCGATACGACGTCATTTTGATGGATGTCCAAATG
UAMH 7800	TATTCTTTTGGCCGAGGACAACGATGTGAACCAGAGGGTTGCCGTCAAGATACTGGAGAAATGCAACCATGGCGTGACCGTTGTCAGCAATGGCCTTCAAGCTGTGGAAGCGATCAAGAAGCGTCGATACGACGTCATTTTGATGGATGTCCAAATG
664	TATTCTTTTGGCCGAGGACAACGATGTGAACCAGAGGGTTGCCGTCAAGATACTGGAGAAATGCAACCATGGCGTGACCGTTGTCAGCAATGGCCTTCAAGCTGTGGAAGCGATCAAGAAGCGTCGATACGACGTCATTTTGATGGATGTCCAAATG
B3003 (UAMH 12046)	TATTCTTTTGGCCGAGGACAACGATGTGAACCAGAGGGTTGCCGTCAAGATACTGGAGAAATGCAACCATGGCGTGACCGTTGTCAGCAATGGCCTTCAAGCTGTGGAAGCGATCAAGAAGCGTCGATACGACGTCATTTTGATGGATGTCCAAATG
CH-10	TATTCTTTTGGCCGAGGACAACGATGTGAACCAGAGGGTTGCCGTCAAGATACTGGAGAAATGCAACCATGGCGTGACCGTTGTCAGCAATGGCCTTCAAGCTGTGGAAGCGATCAAGAAGCGTCGATACGACGTCATTTTGATGGATGTCCAAATG
F270	TATTCTTTTGGCCGAGGACAACGATGTGAACCAGAGGGTTGCCGTCAAGATACTGGAGAAATGCAACCATGGCGTGACCGTTGTCAGCAATGGCCTTCAAGCTGTGGAAGCGATCAAGAAGCGTCGATACGACGTCATTTTGATGGATGTCCAAATG
K940	TATTCTTTTGGCCGAGGACAACGATGTGAACCAGAGGGTTGCCGTCAAGATACTGGAGAAATGCAACCATGGCGTGACCGTTGTCAGCAATGGCCTTCAAGCTGTGGAAGCGATCAAGAAGCGTCGATACGACGTCATTTTGATGGATGTCCAAATG
Ro	TATTCTTTTGGCCGAGGACAACGATGTGAACCAGAGGGTTGCCGTCAAGATACTGGAGAAATGCAACCATGGCGTGACCGTTGTCAGCAATGGCCTTCAAGCTGTGGAAGCGATCAAGAAGCGTCGATACGACGTCATTTTGATGGATGTCCAAATG
396	TATTCTTTTGGCCGAGGACAACGATGTGAACCAGAGGGTTGCCGTCAAGATACTGGAGAAATGCAACCATGGCGTGACCGTTGTCAGCAATGGCCTTCAAGCTGTGGAAGCGATCAAGAAGCGTCGATACGACGTCATTTTGATGGATGTCCAAATG
397	TATTCTTTTGGCCGAGGACAACGATGTGAACCAGAGGGTTGCCGTCAAGATACTGGAGAAATGCAACCATGGCGTGACCGTTGTCAGCAATGGCCTTCAAGCTGTGGAAGCGATCAAGAAGCGTCGATACGACGTCATTTTGATGGATGTCCAAATG
ATCC 28306	TATTCTTTTGGCCGAGGACAACGATGTGAACCAGAGGGTTGCCGTCAAGATACTGGAGAAATGCAACCATGGCGTGACCGTTGTCAGCAATGGCCTTCAAGCTGTGGAAGCGATCAAGAAGCGTCGATACGACGTCATTTTGATGGATGTCCAAATG
En	TATTCTTTTGGCCGAGGACAACGATGTGAACCAGAGGGTTGCCGTCAAGATACTGGAGAAATGCAACCATGGCGTGACCGTTGTCAGCAATGGCCTTCAAGCTGTGGAAGCGATCAAGAAGCGTCGATACGACGTCATTTTGATGGATGTCCAAATG
371	TATTCTTTTGGCCGAGGACAACGATGTGAACCAGAGGGTTGCCGTCAAGATACTGGAGAAATGCAACCATGGCGTGACCGTTGTCAGCAATGGCCTTCAAGCTGTGGAAGCGATCAAGAAGCGTCGATACGACGTCATTTTGATGGATGTCCAAATG
663	TATTCTTTTGGCCGAGGACAACGATGTGAACCAGAGGGTTGCCGTCAAGATACTGGAGAAATGCAACCATGGCGTGACCGTTGTCAGCAATGGCCTTCAAGCTGTGGAAGCGATCAAGAAGCGTCGATACGACGTCATTTTGATGGATGTCCAAATG
ATCC 26197 (GA-1)	TATTCTTTTGGCCGAGGACAACGATGTGAACCAGAGGGTTGCCGTCAAGATACTGGAGAAATGCAACCATGGCGTGACCGTTGTCAGCAATGGCCTTCAAGCTGTGGAAGCGATCAAGAAGCGTCGATACGACGTCATTTTGATGGATGTCCAAATG
ATCC 26199 (V)	TATTCTTTTGGCCGAGGACAACGATGTGAACCAGAGGGTTGCCGTCAAGATACTGGAGAAATGCAACCATGGCGTGACCGTTGTCAGCAATGGCCTTCAAGCTGTGGAAGCGATCAAGAAGCGTCGATACGACGTCATTTTGATGGATGTCCAAATG
ATCC MYA-2586 (ER-3)	TATTCTTTTGGCCGAGGACAACGATGTGAACCAGAGGGTTGCCGTCAAGATACTGGAGAAATGCAACCATGGCGTGACCGTTGTCAGCAATGGCCTTCAAGCTGTGGAAGCGATCAAGAAGCGTCGATACGACGTCATTTTGATGGATGTCCAAATG
FR00346 2009	TATTCTTTTGGCCGAGGACAACGATGTGAACCAGAGGGTTGCCGTCAAGATACTGGAGAAATGCAACCATGGCGTGACCGTTGTCAGCAATGGCCTTCAAGCTGTGGAAGCGATCAAGAAGCGTCGATACGACGTCATTTTGATGGATGTCCAAATG
SF03443 2009	TATTCTTTTGGCCGAGGACAACGATGTGAACCAGAGGGTTGCCGTCAAGATACTGGAGAAATGCAACCATGGCGTGACCGTTGTCAGCAATGGCCTTCAAGCTGTGGAAGCGATCAAGAAGCGTCGATACGACGTCATTTTGATGGATGTCCAAATG
SF05773 2009	TATTCTTTTGGCCGAGGACAACGATGTGAACCAGAGGGTTGCCGTCAAGATACTGGAGAAATGCAACCATGGCGTGACCGTTGTCAGCAATGGCCTTCAAGCTGTGGAAGCGATCAAGAAGCGTCGATACGACGTCATTTTGATGGATGTCCAAATG
SF05792 2009	TATTCTTTTGGCCGAGGACAACGATGTGAACCAGAGGGTTGCCGTCAAGATACTGGAGAAATGCAACCATGGCGTGACCGTTGTCAGCAATGGCCTTCAAGCTGTGGAAGCGATCAAGAAGCGTCGATACGACGTCATTTTGATGGATGTCCAAATG
SF06072 2009	TATTCTTTTGGCCGAGGACAACGATGTGAACCAGAGGGTTGCCGTCAAGATACTGGAGAAATGCAACCATGGCGTGACCGTTGTCAGCAATGGCCTTCAAGCTGTGGAAGCGATCAAGAAGCGTCGATACGACGTCATTTTGATGGATGTCCAAATG
SF06266 2009	TATTCTTTTGGCCGAGGACAACGATGTGAACCAGAGGGTTGCCGTCAAGATACTGGAGAAATGCAACCATGGCGTGACCGTTGTCAGCAATGGCCTTCAAGCTGTGGAAGCGATCAAGAAGCGTCGATACGACGTCATTTTGATGGATGTCCAAATG
SF06354 2009	TATTCTTTTGGCCGAGGACAACGATGTGAACCAGAGGGTTGCCGTCAAGATACTGGAGAAATGCAACCATGGCGTGACCGTTGTCAGCAATGGCCTTCAAGCTGTGGAAGCGATCAAGAAGCGTCGATACGACGTCATTTTGATGGATGTCCAAATG
SF14600 2008	TATTCTTTTGGCCGAGGACAACGATGTGAACCAGAGGGTTGCCGTCAAGATACTGGAGAAATGCAACCATGGCGTGACCGTTGTCAGCAATGGCCTTCAAGCTGTGGAAGCGATCAAGAAGCGTCGATACGACGTCATTTTGATGGATGTCCAAATG
B1566 (UAMH 12045)	TATTCTTTTGGCCGAGGACAACGATGTGAACCAGAGGGTTGCCGTCAAGATACTAGAGAAATGCAGCCATGGCGTGACCGTTGTCAGCAATGGCCTCCAAGCTGTAGAAGCGATCAAGAAGCGTCGATACGACGTTATTTTGATGGATGTCCAAATG
ATCC 62541 (601)	TATTCTTTTGGCCGAGGACAACGATGTGAACCAGAGGGTTGCCGTCAAGATACTAGAGAAATGCAACCATGGCGTGACCGTTGTCAGCAATGGCCTCCAAGCTGTAGAAGCGATCAAGAAGCGTCGATACGACGTTATTTTGATGGATGTCCAAATG
ATCC 66136 (637)	TATTCTTTTGGCCGAGGACAACGATGTGAACCAGAGGGTTGCCGTCAAGATACTAGAGAAATGCAACCATGGCGTGACCGTTGTCAGCAATGGCCTCCAAGCTGTAGAAGCGATCAAGAAGCGTCGATACGACGTTATTTTGATGGATGTCCAAATG
FR00742 2009	TATTCTTTTGGCCGAGGACAACGATGTGAACCAGAGGGTTGCCGTCAAGATACTAGAGAAATGCAACCATGGCGTGACCGTTGTCAGCAATGGCCTCCAAGCTGTAGAAGCGATCAAGAAGCGTCGATACGACGTTATTTTGATGGATGTCCAAATG
TB00016 2005	TATTCTTTTGGCCGAGGACAACGATGTGAACCAGAGGGTTGCCGTCAAGATACTAGAGAAATGCAACCATGGCGTGACCGTTGTCAGCAATGGCCTCCAAGCTGTAGAAGCGATCAAGAAGCGTCGATACGACGTTATTTTGATGGATGTCCAAATG
TB00040 2005	TATTCTTTTGGCCGAGGACAACGATGTGAACCAGAGGGTTGCCGTCAAGATACTAGAGAAATGCAACCATGGCGTGACCGTTGTCAGCAATGGCCTCCAAGCTGTAGAAGCGATCAAGAAGCGTCGATACGACGTTATTTTGATGGATGTCCAAATG

Figure S1: Nucleotide Sequence Alignments (continued)

fads

EXON

TB00042 2005	TTATGCCATTCTTTGCGCTAACTACGAGATTTTTCAACAGTCTCCGCAGCACCTATTATGATAGAGTCATGGGTTTTGATGCCGTGGCCCGGTTCACAGTTAAGTATCAACACTACATGTACTACCCAATTCTTCTCTTTGGCCGCTTCAACCTCTACTTCCTTAGCTGGGAGTATCTCATCATGGGCCTCGGCCCCAAGAAAGGACCTGCCTGGTGGCACCGCTGGTTCGAACTGGTTGGCCAAGTC
UAMH 4042	TTATGCCATTCTTTGCGCTAACTACGAGATTTTTCAACAGTCTCCGCAGCACCTATTATGATAGAGTCATGGGTTTTGATGCCGTGGCCCGGTTCACAGTTAAGTATCAACACTACATGTACTACCCAATTCTTCTCTTTGGCCGCTTCAACCTCTACTTCCTTAGCTGGGAGTATCTCATCATGGGCCTCGGCCCCAAGAAAGGACCTGCCTGGTGGCACCGCTGGTTCGAACTGGTTGGCCAAGTC
UAMH 5634	TTATGCCATTCTTTGCGCTAACTACGAGATTTTTCAACAGTCTCCGCAGCACCTATTATGATAGAGTCATGGGTTTTGATGCCGTGGCCCGGTTCACAGTTAAGTATCAACACTACATGTACTACCCAATTCTTCTCTTTGGCCGCTTCAACCTCTACTTCCTTAGCTGGGAGTATCTCATCATGGGCCTCGGCCCCAAGAAAGGACCTGCCTGGTGGCACCGCTGGTTCGAACTGGTTGGCCAAGTC
664	TTATGCCATTCTTTGCGCTAACTACGAGATTTTTCAACAGTCTCCGCAGCACCTATTATGATAGAGTCATGGGTTTTGATGCCGTGGCCCGGTTCACAGTTAAGTATCAACACTACATGTACTACCCAATTCTTCTCTTTGGCCGCTTCAACCTCTACTTCCTTAGCTGGGAGTATCTCATCATGGGCCTCGGCCCCAAGAAAGGACCTGCCTGGTGGCACCGCTGGTTCGAACTGGTTGGCCAAGTC
ATCC 26197 (GA-1)	TTATGCCATTCTTTGCGCTAACTACGAGATTTTTCAACAGTCTCCGCAGCACCTATTATGATAGAGTCATGGGTTTTGATGCCGTGGCCCGGTTCACAGTTAAGTATCAACACTACATGTACTACCCAATTCTTCTCTTTGGCCGCTTCAACCTCTACTTCCTTAGCTGGGAGTATCTCATCATGGGCCTCGGCCCCAAGAAAGGACCTGCCTGGTGGCACCGCTGGTTCGAACTGGTTGGCCAAGTC
ATCC 26199 (V)	TTATGCCATTCTTTGCGCTAACTACGAGATTTTTCAACAGTCTCCGCAGCACCTATTATGATAGAGTCATGGGTTTTGATGCCGTGGCCCGGTTCACAGTTAAGTATCAACACTACATGTACTACCCAATTCTTCTCTTTGGCCGCTTCAACCTCTACTTCCTTAGCTGGGAGTATCTCATCATGGGCCTCGGCCCCAAGAAAGGACCTGCCTGGTGGCACCGCTGGTTCGAACTGGTTGGCCAAGTC
ATCC 66136 (637)	TTATGCCATTCTTTGCGCTAACTACGAGATTTTTCAACAGTCTCCGCAGCACCTATTATGATAGAGTCATGGGTTTTGATGCCGTGGCCCGGTTCACAGTTAAGTATCAACACTACATGTACTACCCAATTCTTCTCTTTGGCCGCTTCAACCTCTACTTCCTTAGCTGGGAGTATCTCATCATGGGCCTCGGCCCCAAGAAAGGACCTGCCTGGTGGCACCGCTGGTTCGAACTGGTTGGCCAAGTC
ATCC MYA-2586 (ER-3)	TTATGCCATTCTTTGCGCTAACTACGAGATTTTTCAACAGTCTCCGCAGCACCTATTATGATAGAGTCATGGGTTTTGATGCCGTGGCCCGGTTCACAGTTAAGTATCAACACTACATGTACTACCCAATTCTTCTCTTTGGCCGCTTCAACCTCTACTTCCTTAGCTGGGAGTATCTCATCATGGGCCTCGGCCCCAAGAAAGGACCTGCCTGGTGGCACCGCTGGTTCGAACTGGTTGGCCAAGTC
B3003 (UAMH 12046)	TTATGCCATTCTTTGCGCTAACTACGAGATTTTTCAACAGTCTCCGCAGCACCTATTATGATAGAGTCATGGGTTTTGATGCCGTGGCCCGGTTCACAGTTAAGTATCAACACTACATGTACTACCCAATTCTTCTCTTTGGCCGCTTCAACCTCTACTTCCTTAGCTGGGAGTATCTCATCATGGGCCTCGGCCCCAAGAAAGGACCTGCCTGGTGGCACCGCTGGTTCGAACTGGTTGGCCAAGTC
FR00742 2009	TTATGCCATTCTTTGCGCTAACTACGAGATTTTTCAACAGTCTCCGCAGCACCTATTATGATAGAGTCATGGGTTTTGATGCCGTGGCCCGGTTCACAGTTAAGTATCAACACTACATGTACTACCCAATTCTTCTCTTTGGCCGCTTCAACCTCTACTTCCTTAGCTGGGAGTATCTCATCATGGGCCTCGGCCCCAAGAAAGGACCTGCCTGGTGGCACCGCTGGTTCGAACTGGTTGGCCAAGTC
Ro	TTATGCCATTCTTTGCGCTAACTACGAGATTTTTCAACAGTCTCCGCAGCACCTATTATGATAGAGTCATGGGTTTTGATGCCGTGGCCCGGTTCACAGTTAAGTATCAACACTACATGTACTACCCAATTCTTCTCTTTGGCCGCTTCAACCTCTACTTCCTTAGCTGGGAGTATCTCATCATGGGCCTCGGCCCCAAGAAAGGACCTGCCTGGTGGCACCGCTGGTTCGAACTGGTTGGCCAAGTC
TB00016 2005	TTATGCCATTCTTTGCGCTAACTACGAGATTTTTCAACAGTCTCCGCAGCACCTATTATGATAGAGTCATGGGTTTTGATGCCGTGGCCCGGTTCACAGTTAAGTATCAACACTACATGTACTACCCAATTCTTCTCTTTGGCCGCTTCAACCTCTACTTCCTTAGCTGGGAGTATCTCATCATGGGCCTCGGCCCCAAGAAAGGACCTGCCTGGTGGCACCGCTGGTTCGAACTGGTTGGCCAAGTC
UAMH 5584	TTATGCCATTCTTTGCGCTAACTACGAGATTTTTCAACAGTCTCCGCAGCACCTATTATGATAGAGTCATGGGTTTTGATGCCGTGGCCCGGTTCACAGTTAAGTATCAACACTACATGTACTACCCAATTCTTCTCTTTGGCCGCTTCAACCTCTACTTCCTTAGCTGGGAGTATCTCATCATGGGCCTCGGCCCCAAGAAAGGACCTGCCTGGTGGCACCGCTGGTTCGAACTGGTTGGCCAAGTC
371	TTATGCCATTCTTTGCGCTAACTACGAGATTTTTCAACAGTCTCCGCAGCACCTATTATGATAGAGTCATGGGTTTTGATGCCGTGGCCCGGTTCACAGTTAAGTATCAACACTACATGTACTACCCAATTCTTCTCTTTGGCCGCTTCAACCTCTACTTCCTTAGCTGGGAGTATCTCATCATGGGCCTCGGCCCCAAGAAAGGACCTGCCTGGTGGCACCGCTGGTTCGAACTGGTTGGCCAAGTC
396	TTATGCCATTCTTTGCGCTAACTACGAGATTTTTCAACAGTCTCCGCAGCACCTATTATGATAGAGTCATGGGTTTTGATGCCGTGGCCCGGTTCACAGTTAAGTATCAACACTACATGTACTACCCAATTCTTCTCTTTGGCCGCTTCAACCTCTACTTCCTTAGCTGGGAGTATCTCATCATGGGCCTCGGCCCCAAGAAAGGACCTGCCTGGTGGCACCGCTGGTTCGAACTGGTTGGCCAAGTC
397	TTATGCCATTCTTTGCGCTAACTACGAGATTTTTCAACAGTCTCCGCAGCACCTATTATGATAGAGTCATGGGTTTTGATGCCGTGGCCCGGTTCACAGTTAAGTATCAACACTACATGTACTACCCAATTCTTCTCTTTGGCCGCTTCAACCTCTACTTCCTTAGCTGGGAGTATCTCATCATGGGCCTCGGCCCCAAGAAAGGACCTGCCTGGTGGCACCGCTGGTTCGAACTGGTTGGCCAAGTC
663	TTATGCCATTCTTTGCGCTAACTACGAGATTTTTCAACAGTCTCCGCAGCACCTATTATGATAGAGTCATGGGTTTTGATGCCGTGGCCCGGTTCACAGTTAAGTATCAACACTACATGTACTACCCAATTCTTCTCTTTGGCCGCTTCAACCTCTACTTCCTTAGCTGGGAGTATCTCATCATGGGCCTCGGCCCCAAGAAAGGACCTGCCTGGTGGCACCGCTGGTTCGAACTGGTTGGCCAAGTC
ATCC 28306	TTATGCCATTCTTTGCGCTAACTACGAGATTTTTCAACAGTCTCCGCAGCACCTATTATGATAGAGTCATGGGTTTTGATGCCGTGGCCCGGTTCACAGTTAAGTATCAACACTACATGTACTACCCAATTCTTCTCTTTGGCCGCTTCAACCTCTACTTCCTTAGCTGGGAGTATCTCATCATGGGCCTCGGCCCCAAGAAAGGACCTGCCTGGTGGCACCGCTGGTTCGAACTGGTTGGCCAAGTC
CH-10	TTATGCCATTCTTTGCGCTAACTACGAGATTTTTCAACAGTCTCCGCAGCACCTATTATGATAGAGTCATGGGTTTTGATGCCGTGGCCCGGTTCACAGTTAAGTATCAACACTACATGTACTACCCAATTCTTCTCTTTGGCCGCTTCAACCTCTACTTCCTTAGCTGGGAGTATCTCATCATGGGCCTCGGCCCCAAGAAAGGACCTGCCTGGTGGCACCGCTGGTTCGAACTGGTTGGCCAAGTC
En	TTATGCCATTCTTTGCGCTAACTACGAGATTTTTCAACAGTCTCCGCAGCACCTATTATGATAGAGTCATGGGTTTTGATGCCGTGGCCCGGTTCACAGTTAAGTATCAACACTACATGTACTACCCAATTCTTCTCTTTGGCCGCTTCAACCTCTACTTCCTTAGCTGGGAGTATCTCATCATGGGCCTCGGCCCCAAGAAAGGACCTGCCTGGTGGCACCGCTGGTTCGAACTGGTTGGCCAAGTC
F270	TTATGCCATTCTTTGCGCTAACTACGAGATTTTTCAACAGTCTCCGCAGCACCTATTATGATAGAGTCATGGGTTTTGATGCCGTGGCCCGGTTCACAGTTAAGTATCAACACTACATGTACTACCCAATTCTTCTCTTTGGCCGCTTCAACCTCTACTTCCTTAGCTGGGAGTATCTCATCATGGGCCTCGGCCCCAAGAAAGGACCTGCCTGGTGGCACCGCTGGTTCGAACTGGTTGGCCAAGTC
FR00346 2009	TTATGCCATTCTTTGCGCTAACTACGAGATTTTTCAACAGTCTCCGCAGCACCTATTATGATAGAGTCATGGGTTTTGATGCCGTGGCCCGGTTCACAGTTAAGTATCAACACTACATGTACTACCCAATTCTTCTCTTTGGCCGCTTCAACCTCTACTTCCTTAGCTGGGAGTATCTCATCATGGGCCTCGGCCCCAAGAAAGGACCTGCCTGGTGGCACCGCTGGTTCGAACTGGTTGGCCAAGTC
K940	TTATGCCATTCTTTGCGCTAACTACGAGATTTTTCAACAGTCTCCGCAGCACCTATTATGATAGAGTCATGGGTTTTGATGCCGTGGCCCGGTTCACAGTTAAGTATCAACACTACATGTACTACCCAATTCTTCTCTTTGGCCGCTTCAACCTCTACTTCCTTAGCTGGGAGTATCTCATCATGGGCCTCGGCCCCAAGAAAGGACCTGCCTGGTGGCACCGCTGGTTCGAACTGGTTGGCCAAGTC
SF03443 2009	TTATGCCATTCTTTGCGCTAACTACGAGATTTTTCAACAGTCTCCGCAGCACCTATTATGATAGAGTCATGGGTTTTGATGCCGTGGCCCGGTTCACAGTTAAGTATCAACACTACATGTACTACCCAATTCTTCTCTTTGGCCGCTTCAACCTCTACTTCCTTAGCTGGGAGTATCTCATCATGGGCCTCGGCCCCAAGAAAGGACCTGCCTGGTGGCACCGCTGGTTCGAACTGGTTGGCCAAGTC
SF05773 2009	TTATGCCATTCTTTGCGCTAACTACGAGATTTTTCAACAGTCTCCGCAGCACCTATTATGATAGAGTCATGGGTTTTGATGCCGTGGCCCGGTTCACAGTTAAGTATCAACACTACATGTACTACCCAATTCTTCTCTTTGGCCGCTTCAACCTCTACTTCCTTAGCTGGGAGTATCTCATCATGGGCCTCGGCCCCAAGAAAGGACCTGCCTGGTGGCACCGCTGGTTCGAACTGGTTGGCCAAGTC
SF05792 2009	TTATGCCATTCTTTGCGCTAACTACGAGATTTTTCAACAGTCTCCGCAGCACCTATTATGATAGAGTCATGGGTTTTGATGCCGTGGCCCGGTTCACAGTTAAGTATCAACACTACATGTACTACCCAATTCTTCTCTTTGGCCGCTTCAACCTCTACTTCCTTAGCTGGGAGTATCTCATCATGGGCCTCGGCCCCAAGAAAGGACCTGCCTGGTGGCACCGCTGGTTCGAACTGGTTGGCCAAGTC
SF06072 2009	TTATGCCATTCTTTGCGCTAACTACGAGATTTTTCAACAGTCTCCGCAGCACCTATTATGATAGAGTCATGGGTTTTGATGCCGTGGCCCGGTTCACAGTTAAGTATCAACACTACATGTACTACCCAATTCTTCTCTTTGGCCGCTTCAACCTCTACTTCCTTAGCTGGGAGTATCTCATCATGGGCCTCGGCCCCAAGAAAGGACCTGCCTGGTGGCACCGCTGGTTCGAACTGGTTGGCCAAGTC
SF06266 2009	TTATGCCATTCTTTGCGCTAACTACGAGATTTTTCAACAGTCTCCGCAGCACCTATTATGATAGAGTCATGGGTTTTGATGCCGTGGCCCGGTTCACAGTTAAGTATCAACACTACATGTACTACCCAATTCTTCTCTTTGGCCGCTTCAACCTCTACTTCCTTAGCTGGGAGTATCTCATCATGGGCCTCGGCCCCAAGAAAGGACCTGCCTGGTGGCACCGCTGGTTCGAACTGGTTGGCCAAGTC
SF06354 2009	TTATGCCATTCTTTGCGCTAACTACGAGATTTTTCAACAGTCTCCGCAGCACCTATTATGATAGAGTCATGGGTTTTGATGCCGTGGCCCGGTTCACAGTTAAGTATCAACACTACATGTACTACCCAATTCTTCTCTTTGGCCGCTTCAACCTCTACTTCCTTAGCTGGGAGTATCTCATCATGGGCCTCGGCCCCAAGAAAGGACCTGCCTGGTGGCACCGCTGGTTCGAACTGGTTGGCCAAGTC
SF14600 2008	TTATGCCATTCTTTGCGCTAACTACGAGATTTTTCAACAGTCTCCGCAGCACCTATTATGATAGAGTCATGGGTTTTGATGCCGTGGCCCGGTTCACAGTTAAGTATCAACACTACATGTACTACCCAATTCTTCTCTTTGGCCGCTTCAACCTCTACTTCCTTAGCTGGGAGTATCTCATCATGGGCCTCGGCCCCAAGAAAGGACCTGCCTGGTGGCACCGCTGGTTCGAACTGGTTGGCCAAGTC
TB00011 2006	TTATGCCATTCTTTGCGCTAACTACGAGATTTTTCAACAGTCTCCGCAGCACCTATTATGATAGAGTCATGGGTTTTGATGCCGTGGCCCGGTTCACAGTTAAGTATCAACACTACATGTACTACCCAATTCTTCTCTTTGGCCGCTTCAACCTCTACTTCCTTAGCTGGGAGTATCTCATCATGGGCCTCGGCCCCAAGAAAGGACCTGCCTGGTGGCACCGCTGGTTCGAACTGGTTGGCCAAGTC
TB00024 2005	TTATGCCATTCTTTGCGCTAACTACGAGATTTTTCAACAGTCTCCGCAGCACCTATTATGATAGAGTCATGGGTTTTGATGCCGTGGCCCGGTTCACAGTTAAGTATCAACACTACATGTACTACCCAATTCTTCTCTTTGGCCGCTTCAACCTCTACTTCCTTAGCTGGGAGTATCTCATCATGGGCCTCGGCCCCAAGAAAGGACCTGCCTGGTGGCACCGCTGGTTCGAACTGGTTGGCCAAGTC
UAMH 7800	TTATGCCATTCTTTGCGCTAACTACGAGATTTTTCAACAGTCTCCGCAGCACCTATTATGATAGAGTCATGGGTTTTGATGCCGTGGCCCGGTTCACAGTTAAGTATCAACACTACATGTACTACCCAATTCTTCTCTTTGGCCGCTTCAACCTCTACTTCCTTAGCTGGGAGTATCTCATCATGGGCCTCGGCCCCAAGAAAGGACCTGCCTGGTGGCACCGCTGGTTCGAACTGGTTGGCCAAGTC
ATCC 62541(601)	TTATGCCATTCTTTGCGCTAACTACGAGGTTTTTCAACAGTCTCCGCAGCACCTATTATGATAGAGTCATGGGTTTTGATGCCGTGGCCCGGTTCACAGTTAAGTATCAACACTACATGTACTACCCAATTCTTCTCTTTGGCCGCTTCAACCTCTACTTCCTTAGCTGGGAGTATCTCATCATGGGCCTCGGCCCCAAGAAAGGACCTGCCTGGTGGCACCGCTGGTTCGAACTGGTTGGCCAAGTC
B1566 (UAMH 12045)	TTATGCCATTCTTTGCGCTAACTACGAGGTTTTTCAACAGTCTCCGCAGCACCTATTATGATAGAGTCATGGGTTTTGATGCCGTGGCCCGGTTCACAGTTAAGTATCAACACTACATGTACTACCCAATTCTTCTCTTTGGCCGCTTCAACCTCTACTTCCTTAGCTGGGAGTATCTCATCATGGGCCTCGGCCCCAAGAAAGGACCTGCCTGGTGGCACCGCTGGTTCGAACTGGTTGGCCAAGTC
TB00040 2005	TTATGCCATTCTTTGCGCTAACTACGAGGTTTTTCAACAGTCTCCGCAGCACCTATTATGATAGAGTCATGGGTTTTGATGCCGTGGCCCGGTTCACAGTTAAGTATCAACACTACATGTACTACCCAATTCTTCTCTTTGGCCGCTTCAACCTCTACTTCCTTAGCTGGGAGTATCTCATCATGGGCCTCGGCCCCAAGAAAGGACCTGCCTGGTGGCACCGCTGGTTCGAACTGGTTGGCCAAGTC

fads (continued)

EXON

TB00042 2005	TTCTTCTGGACCTGGTTTGGCTACGGAGTTGTCTATGCTTCTATTCCCACATGGACAAGCCGGGTCTTATTTGTGCTCATCTCGCACATGGTCACCGCTCCCCTGCACGTCCAAATTACTCTATCCCACTACGCCATGTCCACCGCTGATCTGGGCATTCATGAATCCTTTGCTCAAAAGATGCTCCGCACCACAATGGACGTGGACTGCCCCAAGTGGCTTGACTTTTTCCATGGCGGCCTCCAGTTC
UAMH 4042	TTCTTCTGGACCTGGTTTGGCTACGGAGTTGTCTATGCTTCTATTCCCACATGGACAAGCCGGGTCTTATTTGTGCTCATCTCGCACATGGTCACCGCTCCCCTGCACGTCCAAATTACTCTATCCCACTACGCCATGTCCACCGCTGATCTGGGCATTCATGAATCCTTTGCTCAAAAGATGCTCCGCACCACAATGGACGTGGACTGCCCCAAGTGGCTTGACTTTTTCCATGGCGGCCTCCAGTTC
UAMH 5634	TTCTTCTGGACCTGGTTTGGCTACGGAGTTGTCTATGCTTCTATTCCCACATGGACAAGCCGGGTCTTATTTGTGCTCATCTCGCACATGGTCACCGCTCCCCTGCACGTCCAAATTACTCTATCCCACTACGCCATGTCCACCGCTGATCTGGGCATTCATGAATCCTTTGCTCAAAAGATGCTCCGCACCACAATGGACGTGGACTGCCCCAAGTGGCTTGACTTTTTCCATGGCGGCCTCCAGTTC
664	TTCTTCTGGACCTGGTTTGGCTACGGAGTTGTCTATGCTTCTATTCCCACATGGACAAGCCGGGTCTTATTTGTGCTCATCTCGCACATGGTCACCGCTCCCCTGCACGTCCAAATTACTCTATCCCACTACGCCATGTCCACCGCTGATCTGGGCATTCATGAATCCTTTGCTCAAAAGATGCTCCGCACCACAATGGACGTGGACTGCCCCAAGTGGCTTGACTTTTTCCATGGCGGCCTCCAGTTC
ATCC 26197 (GA-1)	TTCTTCTGGACCTGGTTTGGCTACGGAGTTGTCTATGCTTCTATTCCCACATGGACAAGCCGGGTCTTATTTGTGCTCATCTCGCACATGGTCACCGCTCCCCTGCACGTCCAAATTACTCTATCCCACTACGCCATGTCCACCGCTGATCTGGGCATTCATGAATCCTTTGCTCAAAAGATGCTCCGCACCACAATGGACGTGGACTGCCCCAAGTGGCTTGACTTTTTCCATGGCGGCCTCCAGTTC
ATCC 26199 (V)	TTCTTCTGGACCTGGTTTGGCTACGGAGTTGTCTATGCTTCTATTCCCACATGGACAAGCCGGGTCTTATTTGTGCTCATCTCGCACATGGTCACCGCTCCCCTGCACGTCCAAATTACTCTATCCCACTACGCCATGTCCACCGCTGATCTGGGCATTCATGAATCCTTTGCTCAAAAGATGCTCCGCACCACAATGGACGTGGACTGCCCCAAGTGGCTTGACTTTTTCCATGGCGGCCTCCAGTTC
ATCC 66136 (637)	TTCTTCTGGACCTGGTTTGGCTACGGAGTTGTCTATGCTTCTATTCCCACATGGACAAGCCGGGTCTTATTTGTGCTCATCTCGCACATGGTCACCGCTCCCCTGCACGTCCAAATTACTCTATCCCACTACGCCATGTCCACCGCTGATCTGGGCATTCATGAATCCTTTGCTCAAAAGATGCTCCGCACCACAATGGACGTGGACTGCCCCAAGTGGCTTGACTTTTTCCATGGCGGCCTCCAGTTC
ATCC MYA-2586 (ER-3)	TTCTTCTGGACCTGGTTTGGCTACGGAGTTGTCTATGCTTCTATTCCCACATGGACAAGCCGGGTCTTATTTGTGCTCATCTCGCACATGGTCACCGCTCCCCTGCACGTCCAAATTACTCTATCCCACTACGCCATGTCCACCGCTGATCTGGGCATTCATGAATCCTTTGCTCAAAAGATGCTCCGCACCACAATGGACGTGGACTGCCCCAAGTGGCTTGACTTTTTCCATGGCGGCCTCCAGTTC
B3003 (UAMH 12046)	TTCTTCTGGACCTGGTTTGGCTACGGAGTTGTCTATGCTTCTATTCCCACATGGACAAGCCGGGTCTTATTTGTGCTCATCTCGCACATGGTCACCGCTCCCCTGCACGTCCAAATTACTCTATCCCACTACGCCATGTCCACCGCTGATCTGGGCATTCATGAATCCTTTGCTCAAAAGATGCTCCGCACCACAATGGACGTGGACTGCCCCAAGTGGCTTGACTTTTTCCATGGCGGCCTCCAGTTC
FR00742 2009	TTCTTCTGGACCTGGTTTGGCTACGGAGTTGTCTATGCTTCTATTCCCACATGGACAAGCCGGGTCTTATTTGTGCTCATCTCGCACATGGTCACCGCTCCCCTGCACGTCCAAATTACTCTATCCCACTACGCCATGTCCACCGCTGATCTGGGCATTCATGAATCCTTTGCTCAAAAGATGCTCCGCACCACAATGGACGTGGACTGCCCCAAGTGGCTTGACTTTTTCCATGGCGGCCTCCAGTTC
Ro	TTCTTCTGGACCTGGTTTGGCTACGGAGTTGTCTATGCTTCTATTCCCACATGGACAAGCCGGGTCTTATTTGTGCTCATCTCGCACATGGTCACCGCTCCCCTGCACGTCCAAATTACTCTATCCCACTACGCCATGTCCACCGCTGATCTGGGCATTCATGAATCCTTTGCTCAAAAGATGCTCCGCACCACAATGGACGTGGACTGCCCCAAGTGGCTTGACTTTTTCCATGGCGGCCTCCAGTTC
TB00016 2005	TTCTTCTGGACCTGGTTTGGCTACGGAGTTGTCTATGCTTCTATTCCCACATGGACAAGCCGGGTCTTATTTGTGCTCATCTCGCACATGGTCACCGCTCCCCTGCACGTCCAAATTACTCTATCCCACTACGCCATGTCCACCGCTGATCTGGGCATTCATGAATCCTTTGCTCAAAAGATGCTCCGCACCACAATGGACGTGGACTGCCCCAAGTGGCTTGACTTTTTCCATGGCGGCCTCCAGTTC
UAMH 5584	TTCTTCTGGACCTGGTTTGGCTACGGAGTTGTCTATGCTTCTATTCCCACATGGACAAGCCGGGTCTTATTTGTGCTCATCTCGCACATGGTCACCGCTCCCCTGCACGTCCAAATTACTCTATCCCACTACGCCATGTCCACCGCTGATCTGGGCATTCATGAATCCTTTGCTCAAAAGATGCTCCGCACCACAATGGACGTGGACTGCCCCAAGTGGCTTGACTTTTTCCATGGCGGCCTCCAGTTC
371	TTCTTCTGGACCTGGTTTGGCTACGGAGTTGTCTATGCTTCTATTCCCACATGGACAAGCCGGGTCTTATTTGTGCTCATCTCGCACATGGTCACCGCTCCCCTGCACGTCCAAATTACTCTATCCCACTACGCCATGTCCACCGCTGATCTGGGCATTCATGAATCCTTTGCTCAAAAGATGCTCCGCACCACAATGGACGTGGACTGCCCCAAGTGGCTTGACTTTTTCCATGGCGGCCTCCAGTTC
396	TTCTTCTGGACCTGGTTTGGCTACGGAGTTGTCTATGCTTCTATTCCCACATGGACAAGCCGGGTCTTATTTGTGCTCATCTCGCACATGGTCACCGCTCCCCTGCACGTCCAAATTACTCTATCCCACTACGCCATGTCCACCGCTGATCTGGGCATTCATGAATCCTTTGCTCAAAAGATGCTCCGCACCACAATGGACGTGGACTGCCCCAAGTGGCTTGACTTTTTCCATGGCGGCCTCCAGTTC
397	TTCTTCTGGACCTGGTTTGGCTACGGAGTTGTCTATGCTTCTATTCCCACATGGACAAGCCGGGTCTTATTTGTGCTCATCTCGCACATGGTCACCGCTCCCCTGCACGTCCAAATTACTCTATCCCACTACGCCATGTCCACCGCTGATCTGGGCATTCATGAATCCTTTGCTCAAAAGATGCTCCGCACCACAATGGACGTGGACTGCCCCAAGTGGCTTGACTTTTTCCATGGCGGCCTCCAGTTC
663	TTCTTCTGGACCTGGTTTGGCTACGGAGTTGTCTATGCTTCTATTCCCACATGGACAAGCCGGGTCTTATTTGTGCTCATCTCGCACATGGTCACCGCTCCCCTGCACGTCCAAATTACTCTATCCCACTACGCCATGTCCACCGCTGATCTGGGCATTCATGAATCCTTTGCTCAAAAGATGCTCCGCACCACAATGGACGTGGACTGCCCCAAGTGGCTTGACTTTTTCCATGGCGGCCTCCAGTTC
ATCC 28306	TTCTTCTGGACCTGGTTTGGCTACGGAGTTGTCTATGCTTCTATTCCCACATGGACAAGCCGGGTCTTATTTGTGCTCATCTCGCACATGGTCACCGCTCCCCTGCACGTCCAAATTACTCTATCCCACTACGCCATGTCCACCGCTGATCTGGGCATTCATGAATCCTTTGCTCAAAAGATGCTCCGCACCACAATGGACGTGGACTGCCCCAAGTGGCTTGACTTTTTCCATGGCGGCCTCCAGTTC
CH-10	TTCTTCTGGACCTGGTTTGGCTACGGAGTTGTCTATGCTTCTATTCCCACATGGACAAGCCGGGTCTTATTTGTGCTCATCTCGCACATGGTCACCGCTCCCCTGCACGTCCAAATTACTCTATCCCACTACGCCATGTCCACCGCTGATCTGGGCATTCATGAATCCTTTGCTCAAAAGATGCTCCGCACCACAATGGACGTGGACTGCCCCAAGTGGCTTGACTTTTTCCATGGCGGCCTCCAGTTC
En	TTCTTCTGGACCTGGTTTGGCTACGGAGTTGTCTATGCTTCTATTCCCACATGGACAAGCCGGGTCTTATTTGTGCTCATCTCGCACATGGTCACCGCTCCCCTGCACGTCCAAATTACTCTATCCCACTACGCCATGTCCACCGCTGATCTGGGCATTCATGAATCCTTTGCTCAAAAGATGCTCCGCACCACAATGGACGTGGACTGCCCCAAGTGGCTTGACTTTTTCCATGGCGGCCTCCAGTTC
F270	TTCTTCTGGACCTGGTTTGGCTACGGAGTTGTCTATGCTTCTATTCCCACATGGACAAGCCGGGTCTTATTTGTGCTCATCTCGCACATGGTCACCGCTCCCCTGCACGTCCAAATTACTCTATCCCACTACGCCATGTCCACCGCTGATCTGGGCATTCATGAATCCTTTGCTCAAAAGATGCTCCGCACCACAATGGACGTGGACTGCCCCAAGTGGCTTGACTTTTTCCATGGCGGCCTCCAGTTC
FR00346 2009	TTCTTCTGGACCTGGTTTGGCTACGGAGTTGTCTATGCTTCTATTCCCACATGGACAAGCCGGGTCTTATTTGTGCTCATCTCGCACATGGTCACCGCTCCCCTGCACGTCCAAATTACTCTATCCCACTACGCCATGTCCACCGCTGATCTGGGCATTCATGAATCCTTTGCTCAAAAGATGCTCCGCACCACAATGGACGTGGACTGCCCCAAGTGGCTTGACTTTTTCCATGGCGGCCTCCAGTTC
K940	TTCTTCTGGACCTGGTTTGGCTACGGAGTTGTCTATGCTTCTATTCCCACATGGACAAGCCGGGTCTTATTTGTGCTCATCTCGCACATGGTCACCGCTCCCCTGCACGTCCAAATTACTCTATCCCACTACGCCATGTCCACCGCTGATCTGGGCATTCATGAATCCTTTGCTCAAAAGATGCTCCGCACCACAATGGACGTGGACTGCCCCAAGTGGCTTGACTTTTTCCATGGCGGCCTCCAGTTC
SF03443 2009	TTCTTCTGGACCTGGTTTGGCTACGGAGTTGTCTATGCTTCTATTCCCACATGGACAAGCCGGGTCTTATTTGTGCTCATCTCGCACATGGTCACCGCTCCCCTGCACGTCCAAATTACTCTATCCCACTACGCCATGTCCACCGCTGATCTGGGCATTCATGAATCCTTTGCTCAAAAGATGCTCCGCACCACAATGGACGTGGACTGCCCCAAGTGGCTTGACTTTTTCCATGGCGGCCTCCAGTTC
SF05773 2009	TTCTTCTGGACCTGGTTTGGCTACGGAGTTGTCTATGCTTCTATTCCCACATGGACAAGCCGGGTCTTATTTGTGCTCATCTCGCACATGGTCACCGCTCCCCTGCACGTCCAAATTACTCTATCCCACTACGCCATGTCCACCGCTGATCTGGGCATTCATGAATCCTTTGCTCAAAAGATGCTCCGCACCACAATGGACGTGGACTGCCCCAAGTGGCTTGACTTTTTCCATGGCGGCCTCCAGTTC
SF05792 2009	TTCTTCTGGACCTGGTTTGGCTACGGAGTTGTCTATGCTTCTATTCCCACATGGACAAGCCGGGTCTTATTTGTGCTCATCTCGCACATGGTCACCGCTCCCCTGCACGTCCAAATTACTCTATCCCACTACGCCATGTCCACCGCTGATCTGGGCATTCATGAATCCTTTGCTCAAAAGATGCTCCGCACCACAATGGACGTGGACTGCCCCAAGTGGCTTGACTTTTTCCATGGCGGCCTCCAGTTC
SF06072 2009	TTCTTCTGGACCTGGTTTGGCTACGGAGTTGTCTATGCTTCTATTCCCACATGGACAAGCCGGGTCTTATTTGTGCTCATCTCGCACATGGTCACCGCTCCCCTGCACGTCCAAATTACTCTATCCCACTACGCCATGTCCACCGCTGATCTGGGCATTCATGAATCCTTTGCTCAAAAGATGCTCCGCACCACAATGGACGTGGACTGCCCCAAGTGGCTTGACTTTTTCCATGGCGGCCTCCAGTTC
SF06266 2009	TTCTTCTGGACCTGGTTTGGCTACGGAGTTGTCTATGCTTCTATTCCCACATGGACAAGCCGGGTCTTATTTGTGCTCATCTCGCACATGGTCACCGCTCCCCTGCACGTCCAAATTACTCTATCCCACTACGCCATGTCCACCGCTGATCTGGGCATTCATGAATCCTTTGCTCAAAAGATGCTCCGCACCACAATGGACGTGGACTGCCCCAAGTGGCTTGACTTTTTCCATGGCGGCCTCCAGTTC
SF06354 2009	TTCTTCTGGACCTGGTTTGGCTACGGAGTTGTCTATGCTTCTATTCCCACATGGACAAGCCGGGTCTTATTTGTGCTCATCTCGCACATGGTCACCGCTCCCCTGCACGTCCAAATTACTCTATCCCACTACGCCATGTCCACCGCTGATCTGGGCATTCATGAATCCTTTGCTCAAAAGATGCTCCGCACCACAATGGACGTGGACTGCCCCAAGTGGCTTGACTTTTTCCATGGCGGCCTCCAGTTC
SF14600 2008	TTCTTCTGGACCTGGTTTGGCTACGGAGTTGTCTATGCTTCTATTCCCACATGGACAAGCCGGGTCTTATTTGTGCTCATCTCGCACATGGTCACCGCTCCCCTGCACGTCCAAATTACTCTATCCCACTACGCCATGTCCACCGCTGATCTGGGCATTCATGAATCCTTTGCTCAAAAGATGCTCCGCACCACAATGGACGTGGACTGCCCCAAGTGGCTTGACTTTTTCCATGGCGGCCTCCAGTTC
TB00011 2006	TTCTTCTGGACCTGGTTTGGCTACGGAGTTGTCTATGCTTCTATTCCCACATGGACAAGCCGGGTCTTATTTGTGCTCATCTCGCACATGGTCACCGCTCCCCTGCACGTCCAAATTACTCTATCCCACTACGCCATGTCCACCGCTGATCTGGGCATTCATGAATCCTTTGCTCAAAAGATGCTCCGCACCACAATGGACGTGGACTGCCCCAAGTGGCTTGACTTTTTCCATGGCGGCCTCCAGTTC
TB00024 2005	TTCTTCTGGACCTGGTTTGGCTACGGAGTTGTCTATGCTTCTATTCCCACATGGACAAGCCGGGTCTTATTTGTGCTCATCTCGCACATGGTCACCGCTCCCCTGCACGTCCAAATTACTCTATCCCACTACGCCATGTCCACCGCTGATCTGGGCATTCATGAATCCTTTGCTCAAAAGATGCTCCGCACCACAATGGACGTGGACTGCCCCAAGTGGCTTGACTTTTTCCATGGCGGCCTCCAGTTC
UAMH 7800	TTCTTCTGGACCTGGTTTGGCTACGGAGTTGTCTATGCTTCTATTCCCACATGGACAAGCCGGGTCTTATTTGTGCTCATCTCGCACATGGTCACCGCTCCCCTGCACGTCCAAATTACTCTATCCCACTACGCCATGTCCACCGCTGATCTGGGCATTCATGAATCCTTTGCTCAAAAGATGCTCCGCACCACAATGGACGTGGACTGCCCCAAGTGGCTTGACTTTTTCCATGGCGGCCTCCAGTTC
ATCC 62541(601)	TTCTTCTGGACCTGGTTTGGCTACGGAGTTGTCTATGCTTCTATTCCCACATGGACAAGCCGGGTCTTATTTGTGCTCATCTCGCACATGGTCACCGCTCCCCTGCACGTCCAAATTACTCTATCCCACTACGCCATGTCCACCGCTGATCTGGGCATTCATGAATCCTTTGCTCAAAAGATGCTCCGCACCACAATGGACGTGGACTGCCCCAAGTGGCTTGACTTTTTCCATGGCGGCCTCCAGTTC
B1566 (UAMH 12045)	TTCTTCTGGACCTGGTTTGGCTACGGAGTTGTCTATGCTTCTATTCCCACATGGACAAGCCGGGTCTTATTTGTGCTCATCTCGCACATGGTCACCGCTCCCCTGCACGTCCAAATTACTCTATCCCACTACGCCATGTCCACCGCTGATCTGGGCATTCATGAATCCTTTGCTCAAAAGATGCTCCGCACCACAATGGACGTGGACTGCCCCAAGTGGCTTGACTTTTTCCATGGCGGCCTCCAGTTC
TB00040 2005	TTCTTCTGGACCTGGTTTGGCTACGGAGTTGTCTATGCTTCTATTCCCACATGGACAAGCCGGGTCTTATTTGTGCTCATCTCGCACATGGTCACCGCTCCCCTGCACGTCCAAATTACTCTATCCCACTACGCCATGTCCACCGCTGATCTGGGCATTCATGAATCCTTTGCTCAAAAGATGCTCCGCACCACAATGGACGTGGACTGCCCCAAGTGGCTTGACTTTTTCCATGGCGGCCTCCAGTTC

Figure S1: Nucleotide Sequence Alignments (continued)

fads (continued)

EXON

TB00042 2005	CAGGCCATCCACCACCTGTACCCTACAATCCCGCGGCATAACCTGCGCAAGACGCAGCGTTTTGTTCAGGAATTTTGCAAGGAGGTTGAAATTCCGTATGCTGTGTTTACGTTCCTCGGTGGGAACAAGAAGGTTATTCA
UAMH 4042	CAGGCCATCCACCACCTGTACCCTACAATCCCGCGGCATAACCTGCGCAAGACGCAGCGTTTTGTTCAGGAATTTTGCAAGGAGGTTGAAATTCCGTATGCTGTGTTTACGTTCCTCGGTGGGAACAAGAAGGTTATTCA
UAMH 5634	CAGGCCATCCACCACCTGTACCCTACAATCCCGCGGCATAACCTGCGCAAGACGCAGCGTTTTGTTCAGGAATTTTGCAAGGAGGTTGAAATTCCGTATGCTGTGTTTACGTTCCTCGGTGGGAACAAGAAGGTTATTCA
664	CAGGCCATCCACCACCTGTACCCTACAATCCCGCGGCATAACCTGCGCAAGACGCAGCGTTTTGTTCAGGAATTTTGCAAGGAGGTTGAAATTCCGTATGCTGTGTTTACGTTCCTCGGTGGGAATAAGAAGGTTATTCA
ATCC 26197 (GA-1)	CAGGCCATCCACCACCTGTACCCTACAATCCCGCGGCATAACCTGCGCAAGACGCAGCGTTTTGTTCAGGAATTTTGCAAGGAGGTTGAAATTCCGTATGCTGTGTTTACGTTCCTCGGTGGGAATAAGAAGGTTATTCA
ATCC 26199 (V)	CAGGCCATCCACCACCTGTACCCTACAATCCCGCGGCATAACCTGCGCAAGACGCAGCGTTTTGTTCAGGAATTTTGCAAGGAGGTTGAAATTCCGTATGCTGTGTTTACGTTCCTCGGTGGGAATAAGAAGGTTATTCA
ATCC 66136 (637)	CAGGCCATCCACCACCTGTACCCTACAATCCCGCGGCATAACCTGCGCAAGACGCAGCGTTTTGTTCAGGAATTTTGCAAGGAGGTTGAAATTCCGTATGCTGTGTTTACGTTCCTCGGTGGGAATAAGAAGGTTATTCA
ATCC MYA-2586 (ER-3)	CAGGCCATCCACCACCTGTACCCTACAATCCCGCGGCATAACCTGCGCAAGACGCAGCGTTTTGTTCAGGAATTTTGCAAGGAGGTTGAAATTCCGTATGCTGTGTTTACGTTCCTCGGTGGGAATAAGAAGGTTATTCA
B3003 (UAMH 12046)	CAGGCCATCCACCACCTGTACCCTACAATCCCGCGGCATAACCTGCGCAAGACGCAGCGTTTTGTTCAGGAATTTTGCAAGGAGGTTGAAATTCCGTATGCTGTGTTTACGTTCCTCGGTGGGAATAAGAAGGTTATTCA
FR00742 2009	CAGGCCATCCACCACCTGTACCCTACAATCCCGCGGCATAACCTGCGCAAGACGCAGCGTTTTGTTCAGGAATTTTGCAAGGAGGTTGAAATTCCGTATGCTGTGTTTACGTTCCTCGGTGGGAATAAGAAGGTTATTCA
Ro	CAGGCCATCCACCACCTGTACCCTACAATCCCGCGGCATAACCTGCGCAAGACGCAGCGTTTTGTTCAGGAATTTTGCAAGGAGGTTGAAATTCCGTATGCTGTGTTTACGTTCCTCGGTGGGAATAAGAAGGTTATTCA
TB00016 2005	CAGGCCATCCACCACCTGTACCCTACAATCCCGCGGCATAACCTGCGCAAGACGCAGCGTTTTGTTCAGGAATTTTGCAAGGAGGTTGAAATTCCGTATGCTGTGTTTACGTTCCTCGGTGGGAATAAGAAGGTTATTCA
UAMH 5584	CAGGCCATCCACCACCTGTACCCTACAATCCCGCGGCATAACCTGCGCAAGACGCAGCGTTTTGTTCAGGAATTTTGCAAGGAGGTTGAAATTCCGTATGCTGTGTTTACGTTCCTCGGTGGGAATAAGAAGGTTATTCA
371	CAGGCCATCCACCACCTGTACCCTACAATCCCGCGGCATAACCTGCGCAAGACGCAGCGTTTTGTACAGGAATTTTGCAAGGAGGTTGAAATTCCGTATGCTGTGTTTACGTTCCTCGGTGGGAATAAGAAGGTTATTCA
396	CAGGCCATCCACCACCTGTACCCTACAATCCCGCGGCATAACCTGCGCAAGACGCAGCGTTTTGTACAGGAATTTTGCAAGGAGGTTGAAATTCCGTATGCTGTGTTTACGTTCCTCGGTGGGAATAAGAAGGTTATTCA
397	CAGGCCATCCACCACCTGTACCCTACAATCCCGCGGCATAACCTGCGCAAGACGCAGCGTTTTGTACAGGAATTTTGCAAGGAGGTTGAAATTCCGTATGCTGTGTTTACGTTCCTCGGTGGGAATAAGAAGGTTATTCA
663	CAGGCCATCCACCACCTGTACCCTACAATCCCGCGGCATAACCTGCGCAAGACGCAGCGTTTTGTACAGGAATTTTGCAAGGAGGTTGAAATTCCGTATGCTGTGTTTACGTTCCTCGGTGGGAATAAGAAGGTTATTCA
ATCC 28306	CAGGCCATCCACCACCTGTACCCTACAATCCCGCGGCATAACCTGCGCAAGACGCAGCGTTTTGTACAGGAATTTTGCAAGGAGGTTGAAATTCCGTATGCTGTGTTTACGTTCCTCGGTGGGAATAAGAAGGTTATTCA
CH-10	CAGGCCATCCACCACCTGTACCCTACAATCCCGCGGCATAACCTGCGCAAGACGCAGCGTTTTGTACAGGAATTTTGCAAGGAGGTTGAAATTCCGTATGCTGTGTTTACGTTCCTCGGTGGGAATAAGAAGGTTATTCA
En	CAGGCCATCCACCACCTGTACCCTACAATCCCGCGGCATAACCTGCGCAAGACGCAGCGTTTTGTACAGGAATTTTGCAAGGAGGTTGAAATTCCGTATGCTGTGTTTACGTTCCTCGGTGGGAATAAGAAGGTTATTCA
F270	CAGGCCATCCACCACCTGTACCCTACAATCCCGCGGCATAACCTGCGCAAGACGCAGCGTTTTGTACAGGAATTTTGCAAGGAGGTTGAAATTCCGTATGCTGTGTTTACGTTCCTCGGTGGGAATAAGAAGGTTATTCA
FR00346 2009	CAGGCCATCCACCACCTGTACCCTACAATCCCGCGGCATAACCTGCGCAAGACGCAGCGTTTTGTACAGGAATTTTGCAAGGAGGTTGAAATTCCGTATGCTGTGTTTACGTTCCTCGGTGGGAATAAGAAGGTTATTCA
K940	CAGGCCATCCACCACCTGTACCCTACAATCCCGCGGCATAACCTGCGCAAGACGCAGCGTTTTGTACAGGAATTTTGCAAGGAGGTTGAAATTCCGTATGCTGTGTTTACGTTCCTCGGTGGGAATAAGAAGGTTATTCA
SF03443 2009	CAGGCCATCCACCACCTGTACCCTACAATCCCGCGGCATAACCTGCGCAAGACGCAGCGTTTTGTACAGGAATTTTGCAAGGAGGTTGAAATTCCGTATGCTGTGTTTACGTTCCTCGGTGGGAATAAGAAGGTTATTCA
SF05773 2009	CAGGCCATCCACCACCTGTACCCTACAATCCCGCGGCATAACCTGCGCAAGACGCAGCGTTTTGTACAGGAATTTTGCAAGGAGGTTGAAATTCCGTATGCTGTGTTTACGTTCCTCGGTGGGAATAAGAAGGTTATTCA
SF05792 2009	CAGGCCATCCACCACCTGTACCCTACAATCCCGCGGCATAACCTGCGCAAGACGCAGCGTTTTGTACAGGAATTTTGCAAGGAGGTTGAAATTCCGTATGCTGTGTTTACGTTCCTCGGTGGGAATAAGAAGGTTATTCA
SF06072 2009	CAGGCCATCCACCACCTGTACCCTACAATCCCGCGGCATAACCTGCGCAAGACGCAGCGTTTTGTACAGGAATTTTGCAAGGAGGTTGAAATTCCGTATGCTGTGTTTACGTTCCTCGGTGGGAATAAGAAGGTTATTCA
SF06266 2009	CAGGCCATCCACCACCTGTACCCTACAATCCCGCGGCATAACCTGCGCAAGACGCAGCGTTTTGTACAGGAATTTTGCAAGGAGGTTGAAATTCCGTATGCTGTGTTTACGTTCCTCGGTGGGAATAAGAAGGTTATTCA
SF06354 2009	CAGGCCATCCACCACCTGTACCCTACAATCCCGCGGCATAACCTGCGCAAGACGCAGCGTTTTGTACAGGAATTTTGCAAGGAGGTTGAAATTCCGTATGCTGTGTTTACGTTCCTCGGTGGGAATAAGAAGGTTATTCA
SF14600 2008	CAGGCCATCCACCACCTGTACCCTACAATCCCGCGGCATAACCTGCGCAAGACGCAGCGTTTTGTACAGGAATTTTGCAAGGAGGTTGAAATTCCGTATGCTGTGTTTACGTTCCTCGGTGGGAATAAGAAGGTTATTCA
TB00011 2006	CAGGCCATCCACCACCTGTACCCTACAATCCCGCGGCATAACCTGCGCAAGACGCAGCGTTTTGTACAGGAATTTTGCAAGGAGGTTGAAATTCCGTATGCTGTGTTTACGTTCCTCGGTGGGAATAAGAAGGTTATTCA
TB00024 2005	CAGGCCATCCACCACCTGTACCCTACAATCCCGCGGCATAACCTGCGCAAGACGCAGCGTTTTGTACAGGAATTTTGCAAGGAGGTTGAAATTCCGTATGCTGTGTTTACGTTCCTCGGTGGGAATAAGAAGGTTATTCA
UAMH 7800	CAGGCCATCCACCACCTGTACCCTACAATCCCGCGGCATAACCTGCGCAAGACGCAGCGTTTTGTACAGGAATTTTGCAAGGAGGTTGAAATTCCGTATGCTGTGTTTACGTTCCTCGGTGGGAATAAGAAGGTTATTCA
ATCC 62541(601)	CAGGCCATCCACCACCTGTACCCTACAATCCCGCGGCATAACCTGCGCAAGACGCAGCGTTTTGTTCAGGAATTTTGCAAGGAGGTTGAAATTCCGTATGCTGTGTTTACGTTCCTCGGTGGGAATAAGAAGGTTATTCA
B1566 (UAMH 12045)	CAGGCCATCCACCACCTGTACCCTACAATCCCGCGGCATAACCTGCGCAAGACGCAGCGTTTTGTTCAGGAATTTTGCAAGGAGGTTGAAATTCCGTATGCTGTGTTTACGTTCCTCGGTGGGAATAAGAAGGTTATTCA
TB00040 2005	CAGGCCATCCACCACCTGTACCCTACAATCCCGCGGCATAACCTGCGCAAGACGCAGCGTTTTGTTCAGGAATTTTGCAAGGAGGTTGAAATTCCGTATGCTGTGTTTACGTTCCTCGGTGGGAATAAGAAGGTTATTCA

Figure S1: Nucleotide Sequence Alignments (continued)

its2

UTR

ATCC 62541 (601)	GAAATGCGATAAGTAATGCGAATTGCAGAATTCCGTGAATCATCGAATCTTTGAACGCACATTGCGCCCTCTGGTATTCCGGGGGGCATGCCTGTCCGAGCGTCATTGCAACCCTCAAGCGCGGCTTGTGTGTTGGGCCTTCGTCCCCCCGTGGACGTGCCCGAAATGCAGCGGCGGCGTCGTGTTCCGGTGCCCGAGCGTATGGGGCTTTGTCACCCGCTCTAGAGGCCCGGCCGGCTCCGGCCCC
ATCC 66136 (637)	GAAATGCGATAAGTAATGCGAATTGCAGAATTCCGTGAATCATCGAATCTTTGAACGCACATTGCGCCCTCTGGTATTCCGGGGGGCATGCCTGTCCGAGCGTCATTGCAACCCTCAAGCGCGGCTTGTGTGTTGGGCCTTCGTCCCCCCGTGGACGTGCCCGAAATGCAGCGGCGGCGTCGTGTTCCGGTGCCCGAGCGTATGGGGCTTTGTCACCCGCTCTAGAGGCCCGGCCGGCTCCGGCCCC
B1566 (UAMH 12045)	GAAATGCGATAAGTAATGCGAATTGCAGAATTCCGTGAATCATCGAATCTTTGAACGCACATTGCGCCCTCTGGTATTCCGGGGGGCATGCCTGTCCGAGCGTCATTGCAACCCTCAAGCGCGGCTTGTGTGTTGGGCCTTCGTCCCCCCGTGGACGTGCCCGAAATGCAGCGGCGGCGTCGTGTTCCGGTGCCCGAGCGTATGGGGCTTTGTCACCCGCTCTAGAGGCCCGGCCGGCTCCGGCCCC
FR00742 2009	GAAATGCGATAAGTAATGCGAATTGCAGAATTCCGTGAATCATCGAATCTTTGAACGCACATTGCGCCCTCTGGTATTCCGGGGGGCATGCCTGTCCGAGCGTCATTGCAACCCTCAAGCGCGGCTTGTGTGTTGGGCCTTCGTCCCCCCGTGGACGTGCCCGAAATGCAGCGGCGGCGTCGTGTTCCGGTGCCCGAGCGTATGGGGCTTTGTCACCCGCTCTAGAGGCCCGGCCGGCTCCGGCCCC
TB00016 2005	GAAATGCGATAAGTAATGCGAATTGCAGAATTCCGTGAATCATCGAATCTTTGAACGCACATTGCGCCCTCTGGTATTCCGGGGGGCATGCCTGTCCGAGCGTCATTGCAACCCTCAAGCGCGGCTTGTGTGTTGGGCCTTCGTCCCCCCGTGGACGTGCCCGAAATGCAGCGGCGGCGTCGTGTTCCGGTGCCCGAGCGTATGGGGCTTTGTCACCCGCTCTAGAGGCCCGGCCGGCTCCGGCCCC
TB00040 2005	GAAATGCGATAAGTAATGCGAATTGCAGAATTCCGTGAATCATCGAATCTTTGAACGCACATTGCGCCCTCTGGTATTCCGGGGGGCATGCCTGTCCGAGCGTCATTGCAACCCTCAAGCGCGGCTTGTGTGTTGGGCCTTCGTCCCCCCGTGGACGTGCCCGAAATGCAGCGGCGGCGTCGTGTTCCGGTGCCCGAGCGTATGGGGCTTTGTCACCCGCTCTAGAGGCCCGGCCGGCTCCGGCCCC
TB00011 2006	GAAATGCGATAAGTAATGTGAATTGCAGAATTCCGTGAATCATCGAATCTTTGAACGCACATTGCGCCCTCTGGTATTCCGGGGGGCATGCCTGTCCGAGCGTCATTGCAACCCTCAAGCGCGGCTTGTGTGTTGGGCCTTCGTCCCCCCGTGGACGTGCCCGAAATGCAGCGGCGGCGTCGTGTTCCGGTGCCCGAGCGTATGGGGCTTTGTCACCCGCTCTAGAGGCCCGGCCGGCTCCGGCCCC
TB00024 2005	GAAATGCGATAAGTAATGTGAATTGCAGAATTCCGTGAATCATCGAATCTTTGAACGCACATTGCGCCCTCTGGTATTCCGGGGGGCATGCCTGTCCGAGCGTCATTGCAACCCTCAAGCGCGGCTTGTGTGTTGGGCCTTCGTCCCCCCGTGGACGTGCCCGAAATGCAGCGGCGGCGTCGTGTTCCGGTGCCCGAGCGTATGGGGCTTTGTCACCCGCTCTAGAGGCCCGGCCGGCTCCGGCCCC
TB00042 2005	GAAATGCGATAAGTAATGTGAATTGCAGAATTCCGTGAATCATCGAATCTTTGAACGCACATTGCGCCCTCTGGTATTCCGGGGGGCATGCCTGTCCGAGCGTCATTGCAACCCTCAAGCGCGGCTTGTGTGTTGGGCCTTCGTCCCCCCGTGGACGTGCCCGAAATGCAGCGGCGGCGTCGTGTTCCGGTGCCCGAGCGTATGGGGCTTTGTCACCCGCTCTAGAGGCCCGGCCGGCTCCGGCCCC
UAMH 5584	GAAATGCGATAAGTAATGTGAATTGCAGAATTCCGTGAATCATCGAATCTTTGAACGCACATTGCGCCCTCTGGTATTCCGGGGGGCATGCCTGTCCGAGCGTCATTGCAACCCTCAAGCGCGGCTTGTGTGTTGGGCCTTCGTCCCCCCGTGGACGTGCCCGAAATGCAGCGGCGGCGTCGTGTTCCGGTGCCCGAGCGTATGGGGCTTTGTCACCCGCTCTAGAGGCCCGGCCGGCTCCGGCCCC
UAMH 7800	GAAATGCGATAAGTAATGTGAATTGCAGAATTCCGTGAATCATCGAATCTTTGAACGCACATTGCGCCCTCTGGTATTCCGGGGGGCATGCCTGTCCGAGCGTCATTGCAACCCTCAAGCGCGGCTTGTGTGTTGGGCCTTCGTCCCCCCGTGGACGTGCCCGAAATGCAGCGGCGGCGTCGTGTTCCGGTGCCCGAGCGTATGGGGCTTTGTCACCCGCTCTAGAGGCCCGGCCGGCTCCGGCCCC
371	GAAATGCGATAAGTAATGTGAATTGCAGAATTCCGTGAATCATCGAATCTTTGAACGCACATTGCGCCCTCTGGTATTCCGGGGGGCATGCCTGTCCGAGCGTCATTGCAACCCTCAAGCGCGGCTTGTGTGTTGGGCCTTCGTCCCCCCGTGGACGTGCCCGAAATGCAGCGGCGGCGTCGTGTTCCGGTGCCCGAGCGTATGGGGCTTTGTCACCCGCTCTAGAGGCCCGGCCGGCTCCGGCCCC
396	GAAATGCGATAAGTAATGTGAATTGCAGAATTCCGTGAATCATCGAATCTTTGAACGCACATTGCGCCCTCTGGTATTCCGGGGGGCATGCCTGTCCGAGCGTCATTGCAACCCTCAAGCGCGGCTTGTGTGTTGGGCCTTCGTCCCCCCGTGGACGTGCCCGAAATGCAGCGGCGGCGTCGTGTTCCGGTGCCCGAGCGTATGGGGCTTTGTCACCCGCTCTAGAGGCCCGGCCGGCTCCGGCCCC
397	GAAATGCGATAAGTAATGTGAATTGCAGAATTCCGTGAATCATCGAATCTTTGAACGCACATTGCGCCCTCTGGTATTCCGGGGGGCATGCCTGTCCGAGCGTCATTGCAACCCTCAAGCGCGGCTTGTGTGTTGGGCCTTCGTCCCCCCGTGGACGTGCCCGAAATGCAGCGGCGGCGTCGTGTTCCGGTGCCCGAGCGTATGGGGCTTTGTCACCCGCTCTAGAGGCCCGGCCGGCTCCGGCCCC
663	GAAATGCGATAAGTAATGTGAATTGCAGAATTCCGTGAATCATCGAATCTTTGAACGCACATTGCGCCCTCTGGTATTCCGGGGGGCATGCCTGTCCGAGCGTCATTGCAACCCTCAAGCGCGGCTTGTGTGTTGGGCCTTCGTCCCCCCGTGGACGTGCCCGAAATGCAGCGGCGGCGTCGTGTTCCGGTGCCCGAGCGTATGGGGCTTTGTCACCCGCTCTAGAGGCCCGGCCGGCTCCGGCCCC
664	GAAATGCGATAAGTAATGTGAATTGCAGAATTCCGTGAATCATCGAATCTTTGAACGCACATTGCGCCCTCTGGTATTCCGGGGGGCATGCCTGTCCGAGCGTCATTGCAACCCTCAAGCGCGGCTTGTGTGTTGGGCCTTCGTCCCCCCGTGGACGTGCCCGAAATGCAGCGGCGGCGTCGTGTTCCGGTGCCCGAGCGTATGGGGCTTTGTCACCCGCTCTAGAGGCCCGGCCGGCTCCGGCCCC
ATCC 26197 (GA-1)	GAAATGCGATAAGTAATGTGAATTGCAGAATTCCGTGAATCATCGAATCTTTGAACGCACATTGCGCCCTCTGGTATTCCGGGGGGCATGCCTGTCCGAGCGTCATTGCAACCCTCAAGCGCGGCTTGTGTGTTGGGCCTTCGTCCCCCCGTGGACGTGCCCGAAATGCAGCGGCGGCGTCGTGTTCCGGTGCCCGAGCGTATGGGGCTTTGTCACCCGCTCTAGAGGCCCGGCCGGCTCCGGCCCC
ATCC 26199 (V)	GAAATGCGATAAGTAATGTGAATTGCAGAATTCCGTGAATCATCGAATCTTTGAACGCACATTGCGCCCTCTGGTATTCCGGGGGGCATGCCTGTCCGAGCGTCATTGCAACCCTCAAGCGCGGCTTGTGTGTTGGGCCTTCGTCCCCCCGTGGACGTGCCCGAAATGCAGCGGCGGCGTCGTGTTCCGGTGCCCGAGCGTATGGGGCTTTGTCACCCGCTCTAGAGGCCCGGCCGGCTCCGGCCCC
ATCC 28306	GAAATGCGATAAGTAATGTGAATTGCAGAATTCCGTGAATCATCGAATCTTTGAACGCACATTGCGCCCTCTGGTATTCCGGGGGGCATGCCTGTCCGAGCGTCATTGCAACCCTCAAGCGCGGCTTGTGTGTTGGGCCTTCGTCCCCCCGTGGACGTGCCCGAAATGCAGCGGCGGCGTCGTGTTCCGGTGCCCGAGCGTATGGGGCTTTGTCACCCGCTCTAGAGGCCCGGCCGGCTCCGGCCCC
ATCC MYA-2586 (ER-3)	GAAATGCGATAAGTAATGTGAATTGCAGAATTCCGTGAATCATCGAATCTTTGAACGCACATTGCGCCCTCTGGTATTCCGGGGGGCATGCCTGTCCGAGCGTCATTGCAACCCTCAAGCGCGGCTTGTGTGTTGGGCCTTCGTCCCCCCGTGGACGTGCCCGAAATGCAGCGGCGGCGTCGTGTTCCGGTGCCCGAGCGTATGGGGCTTTGTCACCCGCTCTAGAGGCCCGGCCGGCTCCGGCCCC
B3003 (UAMH 12046)	GAAATGCGATAAGTAATGTGAATTGCAGAATTCCGTGAATCATCGAATCTTTGAACGCACATTGCGCCCTCTGGTATTCCGGGGGGCATGCCTGTCCGAGCGTCATTGCAACCCTCAAGCGCGGCTTGTGTGTTGGGCCTTCGTCCCCCCGTGGACGTGCCCGAAATGCAGCGGCGGCGTCGTGTTCCGGTGCCCGAGCGTATGGGGCTTTGTCACCCGCTCTAGAGGCCCGGCCGGCTCCGGCCCC
CH-10	GAAATGCGATAAGTAATGTGAATTGCAGAATTCCGTGAATCATCGAATCTTTGAACGCACATTGCGCCCTCTGGTATTCCGGGGGGCATGCCTGTCCGAGCGTCATTGCAACCCTCAAGCGCGGCTTGTGTGTTGGGCCTTCGTCCCCCCGTGGACGTGCCCGAAATGCAGCGGCGGCGTCGTGTTCCGGTGCCCGAGCGTATGGGGCTTTGTCACCCGCTCTAGAGGCCCGGCCGGCTCCGGCCCC
En	GAAATGCGATAAGTAATGTGAATTGCAGAATTCCGTGAATCATCGAATCTTTGAACGCACATTGCGCCCTCTGGTATTCCGGGGGGCATGCCTGTCCGAGCGTCATTGCAACCCTCAAGCGCGGCTTGTGTGTTGGGCCTTCGTCCCCCCGTGGACGTGCCCGAAATGCAGCGGCGGCGTCGTGTTCCGGTGCCCGAGCGTATGGGGCTTTGTCACCCGCTCTAGAGGCCCGGCCGGCTCCGGCCCC
F270	GAAATGCGATAAGTAATGTGAATTGCAGAATTCCGTGAATCATCGAATCTTTGAACGCACATTGCGCCCTCTGGTATTCCGGGGGGCATGCCTGTCCGAGCGTCATTGCAACCCTCAAGCGCGGCTTGTGTGTTGGGCCTTCGTCCCCCCGTGGACGTGCCCGAAATGCAGCGGCGGCGTCGTGTTCCGGTGCCCGAGCGTATGGGGCTTTGTCACCCGCTCTAGAGGCCCGGCCGGCTCCGGCCCC
FR00346 2009	GAAATGCGATAAGTAATGTGAATTGCAGAATTCCGTGAATCATCGAATCTTTGAACGCACATTGCGCCCTCTGGTATTCCGGGGGGCATGCCTGTCCGAGCGTCATTGCAACCCTCAAGCGCGGCTTGTGTGTTGGGCCTTCGTCCCCCCGTGGACGTGCCCGAAATGCAGCGGCGGCGTCGTGTTCCGGTGCCCGAGCGTATGGGGCTTTGTCACCCGCTCTAGAGGCCCGGCCGGCTCCGGCCCC
K940	GAAATGCGATAAGTAATGTGAATTGCAGAATTCCGTGAATCATCGAATCTTTGAACGCACATTGCGCCCTCTGGTATTCCGGGGGGCATGCCTGTCCGAGCGTCATTGCAACCCTCAAGCGCGGCTTGTGTGTTGGGCCTTCGTCCCCCCGTGGACGTGCCCGAAATGCAGCGGCGGCGTCGTGTTCCGGTGCCCGAGCGTATGGGGCTTTGTCACCCGCTCTAGAGGCCCGGCCGGCTCCGGCCCC
Ro	GAAATGCGATAAGTAATGTGAATTGCAGAATTCCGTGAATCATCGAATCTTTGAACGCACATTGCGCCCTCTGGTATTCCGGGGGGCATGCCTGTCCGAGCGTCATTGCAACCCTCAAGCGCGGCTTGTGTGTTGGGCCTTCGTCCCCCCGTGGACGTGCCCGAAATGCAGCGGCGGCGTCGTGTTCCGGTGCCCGAGCGTATGGGGCTTTGTCACCCGCTCTAGAGGCCCGGCCGGCTCCGGCCCC
SF03443 2009	GAAATGCGATAAGTAATGTGAATTGCAGAATTCCGTGAATCATCGAATCTTTGAACGCACATTGCGCCCTCTGGTATTCCGGGGGGCATGCCTGTCCGAGCGTCATTGCAACCCTCAAGCGCGGCTTGTGTGTTGGGCCTTCGTCCCCCCGTGGACGTGCCCGAAATGCAGCGGCGGCGTCGTGTTCCGGTGCCCGAGCGTATGGGGCTTTGTCACCCGCTCTAGAGGCCCGGCCGGCTCCGGCCCC
SF05773 2009	GAAATGCGATAAGTAATGTGAATTGCAGAATTCCGTGAATCATCGAATCTTTGAACGCACATTGCGCCCTCTGGTATTCCGGGGGGCATGCCTGTCCGAGCGTCATTGCAACCCTCAAGCGCGGCTTGTGTGTTGGGCCTTCGTCCCCCCGTGGACGTGCCCGAAATGCAGCGGCGGCGTCGTGTTCCGGTGCCCGAGCGTATGGGGCTTTGTCACCCGCTCTAGAGGCCCGGCCGGCTCCGGCCCC
SF05792 2009	GAAATGCGATAAGTAATGTGAATTGCAGAATTCCGTGAATCATCGAATCTTTGAACGCACATTGCGCCCTCTGGTATTCCGGGGGGCATGCCTGTCCGAGCGTCATTGCAACCCTCAAGCGCGGCTTGTGTGTTGGGCCTTCGTCCCCCCGTGGACGTGCCCGAAATGCAGCGGCGGCGTCGTGTTCCGGTGCCCGAGCGTATGGGGCTTTGTCACCCGCTCTAGAGGCCCGGCCGGCTCCGGCCCC
SF06072 2009	GAAATGCGATAAGTAATGTGAATTGCAGAATTCCGTGAATCATCGAATCTTTGAACGCACATTGCGCCCTCTGGTATTCCGGGGGGCATGCCTGTCCGAGCGTCATTGCAACCCTCAAGCGCGGCTTGTGTGTTGGGCCTTCGTCCCCCCGTGGACGTGCCCGAAATGCAGCGGCGGCGTCGTGTTCCGGTGCCCGAGCGTATGGGGCTTTGTCACCCGCTCTAGAGGCCCGGCCGGCTCCGGCCCC
SF06266 2009	GAAATGCGATAAGTAATGTGAATTGCAGAATTCCGTGAATCATCGAATCTTTGAACGCACATTGCGCCCTCTGGTATTCCGGGGGGCATGCCTGTCCGAGCGTCATTGCAACCCTCAAGCGCGGCTTGTGTGTTGGGCCTTCGTCCCCCCGTGGACGTGCCCGAAATGCAGCGGCGGCGTCGTGTTCCGGTGCCCGAGCGTATGGGGCTTTGTCACCCGCTCTAGAGGCCCGGCCGGCTCCGGCCCC
SF06354 2009	GAAATGCGATAAGTAATGTGAATTGCAGAATTCCGTGAATCATCGAATCTTTGAACGCACATTGCGCCCTCTGGTATTCCGGGGGGCATGCCTGTCCGAGCGTCATTGCAACCCTCAAGCGCGGCTTGTGTGTTGGGCCTTCGTCCCCCCGTGGACGTGCCCGAAATGCAGCGGCGGCGTCGTGTTCCGGTGCCCGAGCGTATGGGGCTTTGTCACCCGCTCTAGAGGCCCGGCCGGCTCCGGCCCC
SF14600 2008	GAAATGCGATAAGTAATGTGAATTGCAGAATTCCGTGAATCATCGAATCTTTGAACGCACATTGCGCCCTCTGGTATTCCGGGGGGCATGCCTGTCCGAGCGTCATTGCAACCCTCAAGCGCGGCTTGTGTGTTGGGCCTTCGTCCCCCCGTGGACGTGCCCGAAATGCAGCGGCGGCGTCGTGTTCCGGTGCCCGAGCGTATGGGGCTTTGTCACCCGCTCTAGAGGCCCGGCCGGCTCCGGCCCC
UAMH 4042	GAAATGCGATAAGTAATGTGAATTGCAGAATTCCGTGAATCATCGAATCTTTGAACGCACATTGCGCCCTCTGGTATTCCGGGGGGCATGCCTGTCCGAGCGTCATTGCAACCCTCAAGCGCGGCTTGTGTGTTGGGCCTTCGTCCCCCCGTGGACGTGCCCGAAATGCAGCGGCGGCGTCGTGTTCCGGTGCCCGAGCGTATGGGGCTTTGTCACCCGCTCTAGAGGCCCGGCCGGCTCCGGCCCC
UAMH 5634	GAAATGCGATAAGTAATGTGAATTGCAGAATTCCGTGAATCATCGAATCTTTGAACGCACATTGCGCCCTCTGGTATTCCGGGGGGCATGCCTGTCCGAGCGTCATTGCAACCCTCAAGCGCGGCTTGTGTGTTGGGCCTTCGTCCCCCCGTGGACGTGCCCGAAATGCAGCGGCGGCGTCGTGTTCCGGTGCCCGAGCGTATGGGGCTTTGTCACCCGCTCTAGAGGCCCGGCCGGCTCCGGCCCC

its2 (continued)

UTR

ATCC 62541 (601)	ATCTCAAACCCTTCGAGGGAGGGCGGTCTTCGGGCCGGTCTCCCCACCAGGTTGACCTCGGATCAGGTAGGAATACCCGCTGAACTTAA
ATCC 66136 (637)	ATCTCAAACCCTTCGAGGGAGGGCGGTCTTCGGGCCGGTCTCCCCACCAGGTTGACCTCGGATCAGGTAGGAATACCCGCTGAACTTAA
B1566 (UAMH 12045)	ATCTCAAACCCTTCGAGGGAGGGCGGTCTTCGGGCCGGTCTCCCCACCAGGTTGACCTCGGATCAGGTAGGAATACCCGCTGAACTTAA
FR00742 2009	ATCTCAAACCCTTCGAGGGAGGGCGGTCTTCGGGCCGGTCTCCCCACCAGGTTGACCTCGGATCAGGTAGGAATACCCGCTGAACTTAA
TB00016 2005	ATCTCAAACCCTTCGAGGGAGGGCGGTCTTCGGGCCGGTCTCCCCACCAGGTTGACCTCGGATCAGGTAGGAATACCCGCTGAACTTAA
TB00040 2005	ATCCCAAACCCTTCGAGGGAGGGCGGTCTTCGGGCCGGTCTCCCCACCAGGTTGACCTCGGATCAGGTAGGAATACCCGCTGAACTTAA
TB00011 2006	ATCTCAAACCCTTCGAGGGAGGGCGGTCTTCAGGCCGGTCTCCCCACCAGGTTGACCTCGGATCAGGTAGGAATACCCGCTGAACTTAA
TB00024 2005	ATCTCAAACCCTTCGAGGGAGGGCGGTCTTCAGGCCGGTCTCCCCACCAGGTTGACCTCGGATCAGGTAGGAATACCCGCTGAACTTAA
TB00042 2005	ATCTCAAACCCTTCGAGGGAGGGCGGTCTTCAGGCCGGTCTCCCCACCAGGTTGACCTCGGATCAGGTAGGAATACCCGCTGAACTTAA
UAMH 5584	ATCTCAAACCCTTCGAGGGAGGGCGGTCTTCAGGCCGGTCTCCCCACCAGGTTGACCTCGGATCAGGTAGGAATACCCGCTGAACTTAA
UAMH 7800	ATCTCAAACCCTTCGAGGGAGGGCGGTCTTCAGGCCGGTCTCCCCACCAGGTTGACCTCGGATCAGGTAGGAATACCCGCTGAACTTAA
371	ATCTCAAACCCTTCGAGGGAGGGCGGTCTTCGGGCCGGTCTCCCCACCAGGTTGACCTCGGATCAGGTAGGAATACCCGCTGAACTTAA
396	ATCTCAAACCCTTCGAGGGAGGGCGGTCTTCGGGCCGGTCTCCCCACCAGGTTGACCTCGGATCAGGTAGGAATACCCGCTGAACTTAA
397	ATCTCAAACCCTTCGAGGGAGGGCGGTCTTCGGGCCGGTCTCCCCACCAGGTTGACCTCGGATCAGGTAGGAATACCCGCTGAACTTAA
663	ATCTCAAACCCTTCGAGGGAGGGCGGTCTTCGGGCCGGTCTCCCCACCAGGTTGACCTCGGATCAGGTAGGAATACCCGCTGAACTTAA
664	ATCTCAAACCCTTCGAGGGAGGGCGGTCTTCGGGCCGGTCTCCCCACCAGGTTGACCTCGGATCAGGTAGGAATACCCGCTGAACTTAA
ATCC 26197 (GA-1)	ATCTCAAACCCTTCGAGGGAGGGCGGTCTTCGGGCCGGTCTCCCCACCAGGTTGACCTCGGATCAGGTAGGAATACCCGCTGAACTTAA
ATCC 26199 (V)	ATCTCAAACCCTTCGAGGGAGGGCGGTCTTCGGGCCGGTCTCCCCACCAGGTTGACCTCGGATCAGGTAGGAATACCCGCTGAACTTAA
ATCC 28306	ATCTCAAACCCTTCGAGGGAGGGCGGTCTTCGGGCCGGTCTCCCCACCAGGTTGACCTCGGATCAGGTAGGAATACCCGCTGAACTTAA
ATCC MYA-2586 (ER-3)	ATCTCAAACCCTTCGAGGGAGGGCGGTCTTCGGGCCGGTCTCCCCACCAGGTTGACCTCGGATCAGGTAGGAATACCCGCTGAACTTAA
B3003 (UAMH 12046)	ATCTCAAACCCTTCGAGGGAGGGCGGTCTTCGGGCCGGTCTCCCCACCAGGTTGACCTCGGATCAGGTAGGAATACCCGCTGAACTTAA
CH-10	ATCTCAAACCCTTCGAGGGAGGGCGGTCTTCGGGCCGGTCTCCCCACCAGGTTGACCTCGGATCAGGTAGGAATACCCGCTGAACTTAA
En	ATCTCAAACCCTTCGAGGGAGGGCGGTCTTCGGGCCGGTCTCCCCACCAGGTTGACCTCGGATCAGGTAGGAATACCCGCTGAACTTAA
F270	ATCTCAAACCCTTCGAGGGAGGGCGGTCTTCGGGCCGGTCTCCCCACCAGGTTGACCTCGGATCAGGTAGGAATACCCGCTGAACTTAA
FR00346 2009	ATCTCAAACCCTTCGAGGGAGGGCGGTCTTCGGGCCGGTCTCCCCACCAGGTTGACCTCGGATCAGGTAGGAATACCCGCTGAACTTAA
K940	ATCTCAAACCCTTCGAGGGAGGGCGGTCTTCGGGCCGGTCTCCCCACCAGGTTGACCTCGGATCAGGTAGGAATACCCGCTGAACTTAA
Ro	ATCTCAAACCCTTCGAGGGAGGGCGGTCTTCGGGCCGGTCTCCCCACCAGGTTGACCTCGGATCAGGTAGGAATACCCGCTGAACTTAA
SF03443 2009	ATCTCAAACCCTTCGAGGGAGGGCGGTCTTCGGGCCGGTCTCCCCACCAGGTTGACCTCGGATCAGGTAGGAATACCCGCTGAACTTAA
SF05773 2009	ATCTCAAACCCTTCGAGGGAGGGCGGTCTTCGGGCCGGTCTCCCCACCAGGTTGACCTCGGATCAGGTAGGAATACCCGCTGAACTTAA
SF05792 2009	ATCTCAAACCCTTCGAGGGAGGGCGGTCTTCGGGCCGGTCTCCCCACCAGGTTGACCTCGGATCAGGTAGGAATACCCGCTGAACTTAA
SF05792 2009	ATCTCAAACCCTTCGAGGGAGGGCGGTCTTCGGGCCGGTCTCCCCACCAGGTTGACCTCGGATCAGGTAGGAATACCCGCTGAACTTAA
SF06266 2009	ATCTCAAACCCTTCGAGGGAGGGCGGTCTTCGGGCCGGTCTCCCCACCAGGTTGACCTCGGATCAGGTAGGAATACCCGCTGAACTTAA
SF06354 2009	ATCTCAAACCCTTCGAGGGAGGGCGGTCTTCGGGCCGGTCTCCCCACCAGGTTGACCTCGGATCAGGTAGGAATACCCGCTGAACTTAA
SF14600 2008	ATCTCAAACCCTTCGAGGGAGGGCGGTCTTCGGGCCGGTCTCCCCACCAGGTTGACCTCGGATCAGGTAGGAATACCCGCTGAACTTAA
UAMH 4042	ATCTCAAACCCTTCGAGGGAGGGCGGTCTTCGGGCCGGTCTCCCCACCAGGTTGACCTCGGATCAGGTAGGAATACCCGCTGAACTTAA
UAMH 5634	ATCTCAAACCCTTCGAGGGAGGGCGGTCTTCGGGCCGGTCTCCCCACCAGGTTGACCTCGGATCAGGTAGGAATACCCGCTGAACTTAA

Figure S1: Nucleotide Sequence Alignments (continued)

pyrF

INTRON / EXON

TB00011 2006	TAAACGGTTATAATGAAAGACTAAGAAATAATAAAATGATATTAATAAGAAGAAGAATAACAATAATAAGACTAACTCCTAAAAACAGGCCTGGGCCCCTACATCACCGTCCTAAAAACCCACATCGACATCCTCACCGACCTCACTCCATCAACCCTCACCAGCCTCCAAGCCCTCGCCAAAAAACACCGCTTCCTCCTCTTTGAAGACCGCAAATTCGTCGACATCGGCAGCACCGTCCAGAA
TB00042 2005	TAAACGGTTATAATGAAAGACTAAGAAATAATAAAATGATATTAATAAGAAGAAGAATAACAATAATAAGACTAACTCCTAAAAACAGGCCTGGGCCCCTACATCACCGTCCTAAAAACCCACATCGACATCCTCACCGACCTCACTCCATCAACCCTCACCAGCCTCCAAGCCCTCGCCAAAAAACACCGCTTCCTCCTCTTTGAAGACCGCAAATTCGTCGACATCGGCAGCACCGTCCAGAA
UAMH 4042	TAAACGGTTATAATGAAAGACTAAGAAATAATAAAATGATATTAATAAGAAGAAGAATAACAATAATAAGACTAACTCCTAAAAACAGGCCTGGGCCCCTACATCACCGTCCTAAAAACCCACATCGACATCCTCACCGACCTCACTCCATCAACCCTCACCAGCCTCCAAGCCCTCGCCAAAAAACACCGCTTCCTCCTCTTTGAAGACCGCAAATTCGTCGACATCGGCAGCACCGTCCAGAA
UAMH 5584	TAAACGGTTATAATGAAAGACTAAGAAATAATAAAATGATATTAATAAGAAGAAGAATAACAATAATAAGACTAACTCCTAAAAACAGGCCTGGGCCCCTACATCACCGTCCTAAAAACCCACATCGACATCCTCACCGACCTCACTCCATCAACCCTCACCAGCCTCCAAGCCCTCGCCAAAAAACACCGCTTCCTCCTCTTTGAAGACCGCAAATTCGTCGACATCGGCAGCACCGTCCAGAA
UAMH 5634	TAAACGGTTATAATGAAAGACTAAGAAATAATAAAATGATATTAATAAGAAGAAGAATAACAATAATAAGACTAACTCCTAAAAACAGGCCTGGGCCCCTACATCACCGTCCTAAAAACCCACATCGACATCCTCACCGACCTCACTCCATCAACCCTCACCAGCCTCCAAGCCCTCGCCAAAAAACACCGCTTCCTCCTCTTTGAAGACCGCAAATTCGTCGACATCGGCAGCACCGTCCAGAA
371	TAAACGGTTATAATGAAAGAATAAGAAATAATAAAATGATATTAATAAGAAGAAGAATAACAATAATAAGACTAACTCCTAAAAACAGGCCTGGGCCCCTACATCACCGTCCTAAAAACCCACATCGACATCCTCACCGACCTCACTCCATCAACCCTCACCAGCCTCCAAGCCCTCGCCAAAAAACACCGCTTCCTCCTCTTTGAAGACCGCAAATTCGTCGACATCGGCAGCACCGTCCAGAA
396	TAAACGGTTATAATGAAAGAATAAGAAATAATAAAATGATATTAATAAGAAGAAGAATAACAATAATAAGACTAACTCCTAAAAACAGGCCTGGGCCCCTACATCACCGTCCTAAAAACCCACATCGACATCCTCACCGACCTCACTCCATCAACCCTCACCAGCCTCCAAGCCCTCGCCAAAAAACACCGCTTCCTCCTCTTTGAAGACCGCAAATTCGTCGACATCGGCAGCACCGTCCAGAA
397	TAAACGGTTATAATGAAAGAATAAGAAATAATAAAATGATATTAATAAGAAGAAGAATAACAATAATAAGACTAACTCCTAAAAACAGGCCTGGGCCCCTACATCACCGTCCTAAAAACCCACATCGACATCCTCACCGACCTCACTCCATCAACCCTCACCAGCCTCCAAGCCCTCGCCAAAAAACACCGCTTCCTCCTCTTTGAAGACCGCAAATTCGTCGACATCGGCAGCACCGTCCAGAA
663	TAAACGGTTATAATGAAAGAATAAGAAATAATAAAATGATATTAATAAGAAGAAGAATAACAATAATAAGACTAACTCCTAAAAACAGGCCTGGGCCCCTACATCACCGTCCTAAAAACCCACATCGACATCCTCACCGACCTCACTCCATCAACCCTCACCAGCCTCCAAGCCCTCGCCAAAAAACACCGCTTCCTCCTCTTTGAAGACCGCAAATTCGTCGACATCGGCAGCACCGTCCAGAA
664	TAAACGGTTATAATGAAAGAATAAGAAATAATAAAATGATATTAATAAGAAGAAGAATAACAATAATAAGACTAACTCCTAAAAACAGGCCTGGGCCCCTACATCACCGTCCTAAAAACCCACATCGACATCCTCACCGACCTCACTCCATCAACCCTCACCAGCCTCCAAGCCCTCGCCAAAAAACACCGCTTCCTCCTCTTTGAAGACCGCAAATTCGTCGACATCGGCAGCACCGTCCAGAA
ATCC 26199 (V)	TAAACGGTTATAATGAAAGAATAAGAAATAATAAAATGATATTAATAAGAAGAAGAATAACAATAATAAGACTAACTCCTAAAAACAGGCCTGGGCCCCTACATCACCGTCCTAAAAACCCACATCGACATCCTCACCGACCTCACTCCATCAACCCTCACCAGCCTCCAAGCCCTCGCCAAAAAACACCGCTTCCTCCTCTTTGAAGACCGCAAATTCGTCGACATCGGCAGCACCGTCCAGAA
ATCC 28306	TAAACGGTTATAATGAAAGAATAAGAAATAATAAAATGATATTAATAAGAAGAAGAATAACAATAATAAGACTAACTCCTAAAAACAGGCCTGGGCCCCTACATCACCGTCCTAAAAACCCACATCGACATCCTCACCGACCTCACTCCATCAACCCTCACCAGCCTCCAAGCCCTCGCCAAAAAACACCGCTTCCTCCTCTTTGAAGACCGCAAATTCGTCGACATCGGCAGCACCGTCCAGAA
ATCC MYA-2586 (ER-3)	TAAACGGTTATAATGAAAGAATAAGAAATAATAAAATGATATTAATAAGAAGAAGAATAACAATAATAAGACTAACTCCTAAAAACAGGCCTGGGCCCCTACATCACCGTCCTAAAAACCCACATCGACATCCTCACCGACCTCACTCCATCAACCCTCACCAGCCTCCAAGCCCTCGCCAAAAAACACCGCTTCCTCCTCTTTGAAGACCGCAAATTCGTCGACATCGGCAGCACCGTCCAGAA
CH-10	TAAACGGTTATAATGAAAGAATAAGAAATAATAAAATGATATTAATAAGAAGAAGAATAACAATAATAAGACTAACTCCTAAAAACAGGCCTGGGCCCCTACATCACCGTCCTAAAAACCCACATCGACATCCTCACCGACCTCACTCCATCAACCCTCACCAGCCTCCAAGCCCTCGCCAAAAAACACCGCTTCCTCCTCTTTGAAGACCGCAAATTCGTCGACATCGGCAGCACCGTCCAGAA
En	TAAACGGTTATAATGAAAGAATAAGAAATAATAAAATGATATTAATAAGAAGAAGAATAACAATAATAAGACTAACTCCTAAAAACAGGCCTGGGCCCCTACATCACCGTCCTAAAAACCCACATCGACATCCTCACCGACCTCACTCCATCAACCCTCACCAGCCTCCAAGCCCTCGCCAAAAAACACCGCTTCCTCCTCTTTGAAGACCGCAAATTCGTCGACATCGGCAGCACCGTCCAGAA
F270	TAAACGGTTATAATGAAAGAATAAGAAATAATAAAATGATATTAATAAGAAGAAGAATAACAATAATAAGACTAACTCCTAAAAACAGGCCTGGGCCCCTACATCACCGTCCTAAAAACCCACATCGACATCCTCACCGACCTCACTCCATCAACCCTCACCAGCCTCCAAGCCCTCGCCAAAAAACACCGCTTCCTCCTCTTTGAAGACCGCAAATTCGTCGACATCGGCAGCACCGTCCAGAA
K940	TAAACGGTTATAATGAAAGAATAAGAAATAATAAAATGATATTAATAAGAAGAAGAATAACAATAATAAGACTAACTCCTAAAAACAGGCCTGGGCCCCTACATCACCGTCCTAAAAACCCACATCGACATCCTCACCGACCTCACTCCATCAACCCTCACCAGCCTCCAAGCCCTCGCCAAAAAACACCGCTTCCTCCTCTTTGAAGACCGCAAATTCGTCGACATCGGCAGCACCGTCCAGAA
SF03443 2009	TAAACGGTTATAATGAAAGAATAAGAAATAATAAAATGATATTAATAAGAAGAAGAATAACAATAATAAGACTAACTCCTAAAAACAGGCCTGGGCCCCTACATCACCGTCCTAAAAACCCATATCGACATCCTCACCGACCTCACTCCATCAACCCTCACCAGCCTCCAAGCCCTCGCCAAAAAACACCGCTTCCTCCTCTTTGAAGACCGCAAATTCGTCGACATCGGCAGCACCGTCCAGAA
SF06266 2009	TAAACGGTTATAATGAAAGAATAAGAAATAATAAAATGATATTAATAAGAAGAAGAATAACAATAATAAGACTAACTCCTAAAAACAGGCCTGGGCCCCTACATCACCGTCCTAAAAACCCATATCGACATCCTCACCGACCTCACTCCATCAACCCTCACCAGCCTCCAAGCCCTCGCCAAAAAACACCGCTTCCTCCTCTTTGAAGACCGCAAATTCGTCGACATCGGCAGCACCGTCCAGAA
B3003 (UAMH 12046)	TAAACGGTTATAATGAAAGAATAAGAAAGAATAAAATGATATTAATAAGAAGAAGAATAACAATAATAAGACTAACTCCTAAAAACAGGCCTGGGCCCCTACATCACCGTCCTAAAAACCCACATCGACATCCTCACCGACCTCACTCCATCAACCCTCACCAGCCTCCAAGCCCTCGCCAAAAAACACCGCTTCCTCCTCTTTGAAGACCGCAAATTCGTCGACATCGGCAGCACCGTCCAGAA
Ro	TAAACGGTTATAATGAAAGAATAAGAAAGAATAAAATGATATTAATAAGAAGAAGAATAACAATAATAAGACTAACTCCTAAAAACAGGCCTGGGCCCCTACATCACCGTCCTAAAAACCCACATCGACATCCTCACCGACCTCACTCCATCAACCCTCACCAGCCTCCAAGCCCTCGCCAAAAAACACCGCTTCCTCCTCTTTGAAGACCGCAAATTCGTCGACATCGGCAGCACCGTCCAGAA
SF05792 2009	TAAACGGTTATAATGAAAGAATAAGAAAGAATAAAATGATATTAATAAGAAGAAGAATAACAATAATAAGACTAACTCCTAAAAACAGGCCTGGGCCCCTACATCACCGTCCTAAAAACCCACATCGACATCCTCACCGACCTCACTCCATCAACCCTCACCAGCCTCCAAGCCCTCGCCAAAAAACACCGCTTCCTCCTCTTTGAAGACCGCAAATTCGTCGACATCGGCAGCACCGTCCAGAA
SF06072 2009	TAAACGGTTATAATGAAAGAATAAGAAAGAATAAAATGATATTAATAAGAAGAAGAATAACAATAATAAGACTAACTCCTAAAAACAGGCCTGGGCCCCTACATCACCGTCCTAAAAACCCACATCGACATCCTCACCGACCTCACTCCATCAACCCTCACCAGCCTCCAAGCCCTCGCCAAAAAACACCGCTTCCTCCTCTTTGAAGACCGCAAATTCGTCGACATCGGCAGCACCGTCCAGAA
TB00024 2005	TAAACGGTTATAATGAAAGAATAAGAAAGAATAAAATGATATTAATAAGAAGAAGAATAACAATAATAAGACTAACTCCTAAAAACAGGCCTGGGCCCCTACATCACCGTCCTAAAAACCCACATCGACATCCTCACCGACCTCACTCCATCAACCCTCACCAGCCTCCAAGCCCTCGCCAAAAAACACCGCTTCCTCCTCTTTGAAGACCGCAAATTCGTCGACATCGGCAGCACCGTCCAGAA
UAMH 7800	TAAACGGTTATAATGAAAGAATAAGAAAGAATAAAATGATATTAATAAGAAGAAGAATAACAATAATAAGACTAACTCCTAAAAACAGGCCTGGGCCCCTACATCACCGTCCTAAAAACCCACATCGACATCCTCACCGACCTCACTCCATCAACCCTCACCAGCCTCCAAGCCCTCGCCAAAAAACACCGCTTCCTCCTCTTTGAAGACCGCAAATTCGTCGACATCGGCAGCACCGTCCAGAA
B1566 (UAMH 12045)	TAAACGGTTATAATGAAAGAATAAGAAAGAATAAAATGATATTAATAAGAAGAAGAATAACAATAATAAGACTAACTCCTAAAAACAGGCCTGGGCCCCTACATCACCGTCCTAAAAACCCACATCGACATCCTCACCGACCTCACTCCATCAACCCTCACCAGCCTCCAAGCCCTCGCCAAAAAACACCGCTTCCTCCTCTTTGAAGACCGCAAATTCGTCGACATCGGCAGCACCGTCCAGAA
FR00742 2009	TAAACGGTTATAATGAAAGAATAAGAAAGAATAAAATGATATTAATAAGAAGAAGAATAACAATAATAAGACTAACTCCTAAAAACAGGCCTGGGCCCCTACATCACCGTCCTAAAAACCCACATCGACATCCTCACCGACCTCACTCCATCAACCCTCACCAGCCTCCAAGCCCTCGCCAAAAAACACCGCTTCCTCCTCTTTGAAGACCGCAAATTCGTCGACATCGGCAGCACCGTCCAGAA
TB00016 2005	TAAACGGTTATAATGAAAGAATAAGAAAGAATAAAATGATATTAATAAGAAGAAGAATAACAATAATAAGACTAACTCCTAAAAACAGGCCTGGGCCCCTACATCACCGTCCTAAAAACCCACATCGACATCCTCACCGACCTCACTCCATCAACCCTCACCAGCCTCCAAGCCCTCGCCAAAAAACACCGCTTCCTCCTCTTTGAAGACCGCAAATTCGTCGACATCGGCAGCACCGTCCAGAA
TB00040 2005	TAAACGGTTATAATGAAAGAATAAGAAAGAATAAAATGATATTAATAAGAAGAAGAATAACAATAATAAGACTAACTCCTAAAAACAGGCCTGGGCCCCTACATCACCGTCCTAAAAACCCACATCGACATCCTCACCGACCTCACTCCATCAACCCTCACCAGCCTCCAAGCCCTCGCCAAAAAACACCGCTTCCTCCTCTTTGAAGACCGCAAATTCGTCGACATCGGCAGCACCGTCCAGAA
ATCC 62541 (601)	TAAACGGTTATAATGAAAGAATAAGAAATAATAAAATGATATTAATAAGAAGAAGAATAACAATAATAAGACTAACTCCTAAAAACAGGCCTGGGCCCCTACATCACCGTCCTAAAAACCCACATCGACATCCTCACCGACCTCACTCCATCAACCCTCACCAGCCTCCAAGCCCTCGCCAAAAAACACCGCTTCCTCCTCTTTGAAGACCGCAAATTCGTCGACATCGGCAGCACCGTCCAGAA
ATCC 66136 (637)	TAAACGGTTATAATGAAAGAATAAGAAATAATAAAATGATATTAATAAGAAGAAGAATAACAATAATAAGACTAACTCCTAAAAACAGGCCTGGGCCCCTACATCACCGTCCTAAAAACCCACATCGACATCCTCACCGACCTCACTCCATCAACCCTCACCAGCCTCCAAGCCCTCGCCAAAAAACACCGCTTCCTCCTCTTTGAAGACCGCAAATTCGTCGACATCGGCAGCACCGTCCAGAA
SF06354 2009	TAAACGGTTATAATGAAAGAATAAGAAATAATAAAATGATATTAATAAGAAGAAGAATAACAATAATAAGACTAACTCCTAAAAACAGGCCTGGGCCCGTACATCACCGTCCTAAAAACCCACATCGACATCCTCACCGACCTCACTCCATCAACCCTCACCAGCCTCCAAGCCCTCGCCAAAAAACACCGCTTCCTCCTCTTTGAAGACCGCAAATTCGTCGACATCGGCAGCACCGTCCAGAA
SF14600 2008	TAAACGGTTATAATGAAAGAATAAGAAATAATAAAATGATATTAATAAGAAGAAGAATAACAATAATAAGACTAACTCCTAAAAACAGGCCTGGGCCCGTACATCACCGTCCTAAAAACCCACATCGACATCCTCACCGACCTCACTCCATCAACCCTCACCAGCCTCCAAGCCCTCGCCAAAAAACACCGCTTCCTCCTCTTTGAAGACCGCAAATTCGTCGACATCGGCAGCACCGTCCAGAA
SF05773 2009	TAAACGGTTATAATGAAAGAATAAGAAAGAATAAAATGATATTAATAAGAAGAAGAATAACAATAATAAGACTAACTCCTAAAAACAGGCCTGGGCCCGTACATCACCGTCCTAAAAACCCACATCGACATCCTCACCGACCTCACTCCATCAACCCTCACCAGCCTCCAAGCCCTCGCCAAAAAACACCGCTTCCTCCTCTTTGAAGACCGCAAATTCGTCGACATCGGCAGCACCGTCCAGAA
ATCC 26197 (GA-1)	TAAACGGTTATAATGAAAGAATAAGAAAGAATAAAATGATATTAATAAGAAGAAGAATAACAATAATAAGACTAACTCCTCAAAACAGGCCTGGGCCCCTACATCACCGTCCTAAAAACCCACATCGACATCCTCACCGACCTCACTCCATCAACCCTCACCAGCCTCCAAGCCCTCGCCAAAAAACACCGCTTCCTCCTCTTTGAAGACCGCAAATTCGTCGACATCGGCAGCACCGTCCAGAA
FR00346 2009	TAAACGGTTATAATGAAAGAATAAGAAAGAATAAAATGATATTAATAAGAAGAAGAATAACAATAATAAGACTAACTCCTCAAAACAGGCCTGGGCCCCTACATCACCGTCCTAAAAACCCACATCGACATCCTCACCGACCTCACTCCATCAACCCTCACCAGCCTCCAAGCCCTCGCCAAAAAACACCGCTTCCTCCTCTTTGAAGACCGCAAATTCGTCGACATCGGCAGCACCGTCCAGAA

pyrF (continued)

EXON

TB00011 2006	ACAATACCACGGCGGCTCCCTGCGCATCTCGGAATGGGCCCACATCGTCAACTGCGCCATGCTCGCCGGCCCGGGCATCGTCGACGCCCTGGCCCAAACCGCCTCCTCCCCCGTTTTCCAAAGAGCGTATGCCGCCGAGGGGCGCGCGCTGCTTATCCTGGCGGAGATGACGTCCAAGGGCTCGATGGCCACGGGACCCTATACGGAGTTGTCGGTGCAGGCGGCTAGGAAGCATCG
TB00042 2005	ACAATACCACGGCGGCTCCCTGCGCATCTCGGAATGGGCCCACATCGTCAACTGCGCCATGCTCGCCGGCCCGGGCATCGTCGACGCCCTGGCCCAAACCGCCTCCTCCCCCGTTTTCCAAAGAGCGTATGCCGCCGAGGGGCGCGCGCTGCTTATCCTGGCGGAGATGACGTCCAAGGGCTCGATGGCCACGGGACCCTATACGGAGTTGTCGGTGCAGGCGGCTAGGAAGCATCG
UAMH 4042	ACAATACCACGGCGGCTCCCTGCGCATCTCGGAATGGGCCCACATCGTCAACTGCGCCATGCTCGCCGGCCCGGGCATCGTCGACGCCCTGGCCCAAACCGCCTCCTCCCCCGTTTTCCAAAGAGCGTATGCCGCCGAGGGGCGCGCGCTGCTTATCCTGGCGGAGATGACGTCCAAGGGCTCGATGGCCACGGGACCCTATACGGAGTTGTCGGTGCAGGCGGCTAGGAAGCATCG
UAMH 5584	ACAATACCACGGCGGCTCCCTGCGCATCTCGGAATGGGCCCACATCGTCAACTGCGCCATGCTCGCCGGCCCGGGCATCGTCGACGCCCTGGCCCAAACCGCCTCCTCCCCCGTTTTCCAAAGAGCGTATGCCGCCGAGGGGCGCGCGCTGCTTATCCTGGCGGAGATGACGTCCAAGGGCTCGATGGCCACGGGACCCTATACGGAGTTGTCGGTGCAGGCGGCTAGGAAGCATCG
UAMH 5634	ACAATACCACGGCGGCTCCCTGCGCATCTCGGAATGGGCCCACATCGTCAACTGCGCCATGCTCGCCGGCCCGGGCATCGTCGACGCCCTGGCCCAAACCGCCTCCTCCCCCGTTTTCCAAAGAGCGTATGCCGCCGAGGGGCGCGCGCTGCTTATCCTGGCGGAGATGACGTCCAAGGGCTCGATGGCCACGGGACCCTATACGGAGTTGTCGGTGCAGGCGGCTAGGAAGCATCG
371	ACAATACCACGGCGGCTCCCTGCGCATCTCGGAATGGGCCCACATCGTCAACTGCGCCATGCTCGCCGGCCCGGGCATCGTCGACGCCCTGGCCCAAACCGCCTCCTCCCCCGTTTTCCAAAGAGCGTATGCCGCCGAGGGGCGCGCGCTGCTTATCCTGGCGGAGATGACGTCCAAGGGCTCGATGGCCACGGGACCCTATACGGAGTTGTCGGTGCAGGCGGCTAGGAAGCATCG
396	ACAATACCACGGCGGCTCCCTGCGCATCTCGGAATGGGCCCACATCGTCAACTGCGCCATGCTCGCCGGCCCGGGCATCGTCGACGCCCTGGCCCAAACCGCCTCCTCCCCCGTTTTCCAAAGAGCGTATGCCGCCGAGGGGCGCGCGCTGCTTATCCTGGCGGAGATGACGTCCAAGGGCTCGATGGCCACGGGACCCTATACGGAGTTGTCGGTGCAGGCGGCTAGGAAGCATCG
397	ACAATACCACGGCGGCTCCCTGCGCATCTCGGAATGGGCCCACATCGTCAACTGCGCCATGCTCGCCGGCCCGGGCATCGTCGACGCCCTGGCCCAAACCGCCTCCTCCCCCGTTTTCCAAAGAGCGTATGCCGCCGAGGGGCGCGCGCTGCTTATCCTGGCGGAGATGACGTCCAAGGGCTCGATGGCCACGGGACCCTATACGGAGTTGTCGGTGCAGGCGGCTAGGAAGCATCG
663	ACAATACCACGGCGGCTCCCTGCGCATCTCGGAATGGGCCCACATCGTCAACTGCGCCATGCTCGCCGGCCCGGGCATCGTCGACGCCCTGGCCCAAACCGCCTCCTCCCCCGTTTTCCAAAGAGCGTATGCCGCCGAGGGGCGCGCGCTGCTTATCCTGGCGGAGATGACGTCCAAGGGCTCGATGGCCACGGGACCCTATACGGAGTTGTCGGTGCAGGCGGCTAGGAAGCATCG
664	ACAATACCACGGCGGCTCCCTGCGCATCTCGGAATGGGCCCACATCGTCAACTGCGCCATGCTCGCCGGCCCGGGCATCGTCGACGCCCTGGCCCAAACCGCCTCCTCCCCCGTTTTCCAAAGAGCGTATGCCGCCGAGGGGCGCGCGCTGCTTATCCTGGCGGAGATGACGTCCAAGGGCTCGATGGCCACGGGACCCTATACGGAGTTGTCGGTGCAGGCGGCTAGGAAGCATCG
ATCC 26199 (V)	ACAATACCACGGCGGCTCCCTGCGCATCTCGGAATGGGCCCACATCGTCAACTGCGCCATGCTCGCCGGCCCGGGCATCGTCGACGCCCTGGCCCAAACCGCCTCCTCCCCCGTTTTCCAAAGAGCGTATGCCGCCGAGGGGCGCGCGCTGCTTATCCTGGCGGAGATGACGTCCAAGGGCTCGATGGCCACGGGACCCTATACGGAGTTGTCGGTGCAGGCGGCTAGGAAGCATCG
ATCC 28306	ACAATACCACGGCGGCTCCCTGCGCATCTCGGAATGGGCCCACATCGTCAACTGCGCCATGCTCGCCGGCCCGGGCATCGTCGACGCCCTGGCCCAAACCGCCTCCTCCCCCGTTTTCCAAAGAGCGTATGCCGCCGAGGGGCGCGCGCTGCTTATCCTGGCGGAGATGACGTCCAAGGGCTCGATGGCCACGGGACCCTATACGGAGTTGTCGGTGCAGGCGGCTAGGAAGCATCG
ATCC MYA-2586 (ER-3)	ACAATACCACGGCGGCTCCCTGCGCATCTCGGAATGGGCCCACATCGTCAACTGCGCCATGCTCGCCGGCCCGGGCATCGTCGACGCCCTGGCCCAAACCGCCTCCTCCCCCGTTTTCCAAAGAGCGTATGCCGCCGAGGGGCGCGCGCTGCTTATCCTGGCGGAGATGACGTCCAAGGGCTCGATGGCCACGGGACCCTATACGGAGTTGTCGGTGCAGGCGGCTAGGAAGCATCG
CH-10	ACAATACCACGGCGGCTCCCTGCGCATCTCGGAATGGGCCCACATCGTCAACTGCGCCATGCTCGCCGGCCCGGGCATCGTCGACGCCCTGGCCCAAACCGCCTCCTCCCCCGTTTTCCAAAGAGCGTATGCCGCCGAGGGGCGCGCGCTGCTTATCCTGGCGGAGATGACGTCCAAGGGCTCGATGGCCACGGGACCCTATACGGAGTTGTCGGTGCAGGCGGCTAGGAAGCATCG
En	ACAATACCACGGCGGCTCCCTGCGCATCTCGGAATGGGCCCACATCGTCAACTGCGCCATGCTCGCCGGCCCGGGCATCGTCGACGCCCTGGCCCAAACCGCCTCCTCCCCCGTTTTCCAAAGAGCGTATGCCGCCGAGGGGCGCGCGCTGCTTATCCTGGCGGAGATGACGTCCAAGGGCTCGATGGCCACGGGACCCTATACGGAGTTGTCGGTGCAGGCGGCTAGGAAGCATCG
F270	ACAATACCACGGCGGCTCCCTGCGCATCTCGGAATGGGCCCACATCGTCAACTGCGCCATGCTCGCCGGCCCGGGCATCGTCGACGCCCTGGCCCAAACCGCCTCCTCCCCCGTTTTCCAAAGAGCGTATGCCGCCGAGGGGCGCGCGCTGCTTATCCTGGCGGAGATGACGTCCAAGGGCTCGATGGCCACGGGACCCTATACGGAGTTGTCGGTGCAGGCGGCTAGGAAGCATCG
K940	ACAATACCACGGCGGCTCCCTGCGCATCTCGGAATGGGCCCACATCGTCAACTGCGCCATGCTCGCCGGCCCGGGCATCGTCGACGCCCTGGCCCAAACCGCCTCCTCCCCCGTTTTCCAAAGAGCGTATGCCGCCGAGGGGCGCGCGCTGCTTATCCTGGCGGAGATGACGTCCAAGGGCTCGATGGCCACGGGACCCTATACGGAGTTGTCGGTGCAGGCGGCTAGGAAGCATCG
SF03443 2009	ACAATACCACGGCGGCTCCCTGCGCATCTCGGAATGGGCCCACATCGTCAACTGCGCCATGCTCGCCGGCCCGGGCATCGTCGACGCCCTGGCCCAAACCGCCTCCTCCCCCGTTTTCCAAAGAGCGTATGCCGCCGAGGGGCGCGCGCTGCTTATCCTGGCGGAGATGACGTCCAAGGGCTCGATGGCCACGGGACCCTATACGGAGTTGTCGGTGCAGGCGGCTAGGAAGCATCG
SF06266 2009	ACAATACCACGGCGGCTCCCTGCGCATCTCGGAATGGGCCCACATCGTCAACTGCGCCATGCTCGCCGGCCCGGGCATCGTCGACGCCCTGGCCCAAACCGCCTCCTCCCCCGTTTTCCAAAGAGCGTATGCCGCCGAGGGGCGCGCGCTGCTTATCCTGGCGGAGATGACGTCCAAGGGCTCGATGGCCACGGGACCCTATACGGAGTTGTCGGTGCAGGCGGCTAGGAAGCATCG
B3003 (UAMH 12046)	ACAATACCACGGCGGCTCCCTGCGCATCTCGGAATGGGCCCACATCGTCAACTGCGCCATGCTCGCCGGCCCGGGCATCGTCGACGCCCTGGCCCAAACCGCCTCCTCCCCCGTTTTCCAAAGAGCGTATGCCGCCGAGGGGCGCGCGCTGCTTATCCTGGCGGAGATGACGTCCAAGGGCTCGATGGCCACGGGACCCTATACGGAGTTGTCGGTGCAGGCGGCTAGGAAGCATCG
Ro	ACAATACCACGGCGGCTCCCTGCGCATCTCGGAATGGGCCCACATCGTCAACTGCGCCATGCTCGCCGGCCCGGGCATCGTCGACGCCCTGGCCCAAACCGCCTCCTCCCCCGTTTTCCAAAGAGCGTATGCCGCCGAGGGGCGCGCGCTGCTTATCCTGGCGGAGATGACGTCCAAGGGCTCGATGGCCACGGGACCCTATACGGAGTTGTCGGTGCAGGCGGCTAGGAAGCATCG
SF05792 2009	ACAATACCACGGCGGCTCCCTGCGCATCTCGGAATGGGCCCACATCGTCAACTGCGCCATGCTCGCCGGCCCGGGCATCGTCGACGCCCTGGCCCAAACCGCCTCCTCCCCCGTTTTCCAAAGAGCGTATGCCGCCGAGGGGCGCGCGCTGCTTATCCTGGCGGAGATGACGTCCAAGGGCTCGATGGCCACGGGACCCTATACGGAGTTGTCGGTGCAGGCGGCTAGGAAGCATCG
SF06072 2009	ACAATACCACGGCGGCTCCCTGCGCATCTCGGAATGGGCCCACATCGTCAACTGCGCCATGCTCGCCGGCCCGGGCATCGTCGACGCCCTGGCCCAAACCGCCTCCTCCCCCGTTTTCCAAAGAGCGTATGCCGCCGAGGGGCGCGCGCTGCTTATCCTGGCGGAGATGACGTCCAAGGGCTCGATGGCCACGGGACCCTATACGGAGTTGTCGGTGCAGGCGGCTAGGAAGCATCG
TB00024 2005	ACAATACCACGGCGGCTCCCTGCGCATCTCGGAATGGGCCCACATCGTCAACTGCGCCATGCTCGCCGGCCCGGGCATCGTCGACGCCCTGGCCCAAACCGCCTCCTCCCCCGTTTTCCAAAGAGCGTATGCCGCCGAGGGGCGCGCGCTGCTTATCCTGGCGGAGATGACGTCCAAGGGCTCGATGGCCACGGGACCCTATACGGAGTTGTCGGTGCAGGCGGCTAGGAAGCATCG
UAMH 7800	ACAATACCACGGCGGCTCCCTGCGCATCTCGGAATGGGCCCACATCGTCAACTGCGCCATGCTCGCCGGCCCGGGCATCGTCGACGCCCTGGCCCAAACCGCCTCCTCCCCCGTTTTCCAAAGAGCGTATGCCGCCGAGGGGCGCGCGCTGCTTATCCTGGCGGAGATGACGTCCAAGGGCTCGATGGCCACGGGACCCTATACGGAGTTGTCGGTGCAGGCGGCTAGGAAGCATCG
B1566 (UAMH 12045)	ACAATACCACGGCGGCTCCCTGCGCATCTCGGAATGGGCCCACATCGTCAACTGCGCCATGCTCGCCGGCCCGGGCATCGTCGACGCCCTGGCCCAAACCGCCTCCTCCCCCGTTTTCCAAAGAGCGTATGCCGCCGAGGGGCGCGCGCTGCTTATCCTGGCGGAGATGACGTCCAAGGGCTCGATGGCCACGGGACCCTACACGGAGTTGTCGGTGCAGGCGGCTAGGAAGCATCG
FR00742 2009	ACAATACCACGGCGGCTCCCTGCGCATCTCGGAATGGGCCCACATCGTCAACTGCGCCATGCTCGCCGGCCCGGGCATCGTCGACGCCCTGGCCCAAACCGCCTCCTCCCCCGTTTTCCAAAGAGCGTATGCCGCCGAGGGGCGCGCGCTGCTTATCCTGGCGGAGATGACGTCCAAGGGCTCGATGGCCACGGGACCCTACACGGAGTTGTCGGTGCAGGCGGCTAGGAAGCATCG
TB00016 2005	ACAATACCACGGCGGCTCCCTGCGCATCTCGGAATGGGCCCACATCGTCAACTGCGCCATGCTCGCCGGCCCGGGCATCGTCGACGCCCTGGCCCAAACCGCCTCCTCCCCCGTTTTCCAAAGAGCGTATGCCGCCGAGGGGCGCGCGCTGCTTATCCTGGCGGAGATGACGTCCAAGGGCTCGATGGCCACGGGACCCTACACGGAGTTGTCGGTGCAGGCGGCTAGGAAGCATCG
TB00040 2005	ACAATACCACGGCGGCTCCCTGCGCATCTCGGAATGGGCCCACATCGTCAACTGCGCCATGCTCGCCGGCCCGGGCATCGTCGACGCCCTGGCCCAAACCGCCTCCTCCCCCGTTTTCCAAAGAGCGTATGCCGCCGAGGGGCGCGCGCTGCTTATCCTGGCGGAGATGACGTCCAAGGGCTCGATGGCCACGGGACCCTACACGGAGTTGTCGGTGCAGGCGGCTAGGAAGCATCG
ATCC 62541 (601)	ACAATACCACGGCGGCTCCCTGCGCATCTCGGAATGGGCCCACATCGTCAACTGCGCCATGCTCGCCGGCCCGGGCATCGTCGACGCCCTGGCCCAAACCGCCTCCTCCCCCGTTTTCCAAAGAGCGTATGCCGCCGAGGGGCGCGCGCTGCTTATCCTGGCGGAGATGACGTCCAAGGGCTCGATGGCCACGGGACCCTACACGGAGTTGTCGGTGCAGGCGGCTAGGAAGCATCG
ATCC 66136 (637)	ACAATACCACGGCGGCTCCCTGCGCATCTCGGAATGGGCCCACATCGTCAACTGCGCCATGCTCGCCGGCCCGGGCATCGTCGACGCCCTGGCCCAAACCGCCTCCTCCCCCGTTTTCCAAAGAGCGTATGCCGCCGAGGGGCGCGCGCTGCTTATCCTGGCGGAGATGACGTCCAAGGGCTCGATGGCCACGGGACCCTACACGGAGTTGTCGGTGCAGGCGGCTAGGAAGCATCG
SF06354 2009	ACAATACCACGGCGGCTCCCTGCGCATCTCGGAATGGGCCCACATCGTCAACTGCGCCATGCTCGCCGGCCCGGGCATCGTCGACGCCCTGGCCCAAACCGCCTCCTCCCCCGTTTTCCAAAGAGCGTATGCCGCCGAGGGGCGCGCGCTGCTTATCCTGGCGGAGATGACGTCCAAGGGCTCGATGGCCACGGGACCCTATACGGAGTTGTCGGTGCAGGCGGCTAGGAAGCATCG
SF14600 2008	ACAATACCACGGCGGCTCCCTGCGCATCTCGGAATGGGCCCACATCGTCAACTGCGCCATGCTCGCCGGCCCGGGCATCGTCGACGCCCTGGCCCAAACCGCCTCCTCCCCCGTTTTCCAAAGAGCGTATGCCGCCGAGGGGCGCGCGCTGCTTATCCTGGCGGAGATGACGTCCAAGGGCTCGATGGCCACGGGACCCTATACGGAGTTGTCGGTGCAGGCGGCTAGGAAGCATCG
SF05773 2009	ACAATACCACGGCGGCTCCCTGCGCATCTCGGAATGGGCCCACATCGTCAACTGCGCCATGCTCGCCGGCCCGGGCATCGTCGACGCCCTGGCCCAAACCGCCTCCTCCCCCGTTTTCCAAAGAGCGTATGCCGCCGAGGGGCGCGCGCTGCTTATCCTGGCGGAGATGACGTCCAAGGGCTCGATGGCCACGGGACCCTATACGGAGTTGTCGGTGCAGGCGGCTAGGAAGCATCG
ATCC 26197 (GA-1)	ACAATACCACGGCGGCTCCCTGCGCATCTCGGAATGGGCCCACATCGTCAACTGCGCCATGCTCGCCGGCCCGGGCATCGTCGACGCCCTGGCCCAAACCGCCTCCTCCCCCGTTTTCCAAAGAGCGTATGCCGCCGAGGGGCGCGCGCTGCTTATCCTGGCGGAGATGACGTCCAAGGGCTCGATGGCCACGGGACCCTATACGGAGTTGTCGGTGCAGGCGGCTAGGAAGCATCG
FR00346 2009	ACAATACCACGGCGGCTCCCTGCGCATCTCGGAATGGGCCCACATCGTCAACTGCGCCATGCTCGCCGGCCCGGGCATCGTCGACGCCCTGGCCCAAACCGCCTCCTCCCCCGTTTTCCAAAGAGCGTATGCCGCCGAGGGGCGCGCGCTGCTTATCCTGGCGGAGATGACGTCCAAGGGCTCGATGGCCACGGGACCCTATACGGAGTTGTCGGTGCAGGCGGCTAGGAAGCATCG

Figure S1: Nucleotide Sequence Alignments (continued)

tub1

UTR — EXON — INTRON — EXON — INTRON

SF03443 2009	CATTTAGCACAACATACCGAATCCTCCACCACAGCCATCCACCATGAGAGAGGTAGTTAGTTTGAACGGTACGTCGGTCCTTGTTGCTACAGTTTATCTGTATATTTATTCATTTAGCTATAGTCCTTGCTGCTGATATCAACGTGCTGTAAACAGTCGGCCAGGCGGGTTGCCAAATCGCAAACTCCTGTTGGGAAGTACGACTTCACCACTCCATATCACTCGTACTTTTTATTATATCTTTATGCG
SF05773 2009	CATTTAGCACAACATACCGAATCCTCCACCACAGCCATCCACCATGAGAGAGGTAGTTAGTTTGAACGGTACGTCGGTCCTTGTTGCTACAGTTTATCTGTATATTTATTCATTTAGCTATAGTCCTTGCTGCTGATATCAACGTGCTGTAAACAGTCGGCCAGGCGGGTTGCCAAATCGCAAACTCCTGTTGGGAAGTACGACTTCACCACTCCATATCACTCGTACTTTTTATTATATCTTTATGCG
B1566 (UAMH 12045)	CATTTAGCACAACATACCGAATCCTCCACCACAGCCATCCACCATGAGAGAGGTAGTTAGTTTGAACGGTACGTCGGTCCTTGTTGCTACAGTTTATCTGTATATTTATTCATTTAGCTATAGTCCTTGCTGCTGATATCAACGTGCTGTAAACAGTCGGCCAGGCGGGTTGCCAAATCGCAAACTCCTGTTGGGAAGTACGACTTCACCACTCCATATCACTCGTACTTTTTATTATATCTTTATGCG
TB00016 2005	CATTTAGCACAACATACCGAATCCTCCACCACAGCCATCCACCATGAGAGAGGTAGTTAGTTTGAACGGTACGTCGGTCCTTGTTGCTACAGTTTATCTGTATATTTATTCATTTAGCTATAGTCCTTGCTGCTGATATCAACGTGCTGTAAACAGTCGGCCAGGCGGGTTGCCAAATCGCAAACTCCTGTTGGGAAGTACGACTTCACCACTCCATATCACTCGTACTTTTTATTATATCTTTATGCG
SF06072 2009	CATTTAGCACAACATACCGAATCCTCCACCACAGCCATCCACCATGAGAGAGGTAGTTAGTTTGAACGGTACGTCGGTCCTTGTTGCTACAGTTTATCTGTATATTTATTCATTTAGCTATAGTCCTTGCTGCTGATATCAACGTGCTGTAAACAGTCGGCCAGGCGGGTTGCCAAATCGCAAACTCCTGTTGGGAAGTACGACTTCACCACTCCATATCACTCGTACTTTTTATTATATCTTTATGCG
SF06354 2009	CATTTAGCACAACATACCGAATCCTCCACCACAGCCATCCACCATGAGAGAGGTAGTTAGTTTGAACGGTACGTCGGTCCTTGTTGCTACAGTTTATCTGTATATTTATTCATTTAGCTATAGTCCTTGCTGCTGATATCAACGTGCTGTAAACAGTCGGCCAGGCGGGTTGCCAAATCGCAAACTCCTGTTGGGAAGTACGACTTCACCACTCCATATCACTCGTACTTTTTATTATATCTTTATGCG
371	CATTTAGCACAACATACCGAATCCTCCACCACAGCCATCCACCATGAGAGAGGTAGTTAGTTTGAACGGTACGTCGGTCCTTGTTGCTACAGTTTATCTGTATATTTATTCATTTAGCTATAGTCCTTGCTGCTGATATCAACGTGCTGTAAACAGTCGGCCAGGCGGGTTGCCAAATCGCAAACTCCTGTTGGGAAGTACGACTTCACCACTCCATATCACTCGTACTTTTTATTATATCTTTATGCG
663	CATTTAGCACAACATACCGAATCCTCCACCACAGCCATCCACCATGAGAGAGGTAGTTAGTTTGAACGGTACGTCGGTCCTTGTTGCTACAGTTTATCTGTATATTTATTCATTTAGCTATAGTCCTTGCTGCTGATATCAACGTGCTGTAAACAGTCGGCCAGGCGGGTTGCCAAATCGCAAACTCCTGTTGGGAAGTACGACTTCACCACTCCATATCACTCGTACTTTTTATTATATCTTTATGCG
664	CATTTAGCACAACATACCGAATCCTCCACCACAGCCATCCACCATGAGAGAGGTAGTTAGTTTGAACGGTACGTCGGTCCTTGTTGCTACAGTTTATCTGTATATTTATTCATTTAGCTATAGTCCTTGCTGCTGATATCAACGTGCTGTAAACAGTCGGCCAGGCGGGTTGCCAAATCGCAAACTCCTGTTGGGAAGTACGACTTCACCACTCCATATCACTCGTACTTTTTATTATATCTTTATGCG
ATCC 26197 (GA-1)	CATTTAGCACAACATACCGAATCCTCCACCACAGCCATCCACCATGAGAGAGGTAGTTAGTTTGAACGGTACGTCGGTCCTTGTTGCTACAGTTTATCTGTATATTTATTCATTTAGCTATAGTCCTTGCTGCTGATATCAACGTGCTGTAAACAGTCGGCCAGGCGGGTTGCCAAATCGCAAACTCCTGTTGGGAAGTACGACTTCACCACTCCATATCACTCGTACTTTTTATTATATCTTTATGCG
ATCC 26199 (V)	CATTTAGCACAACATACCGAATCCTCCACCACAGCCATCCACCATGAGAGAGGTAGTTAGTTTGAACGGTACGTCGGTCCTTGTTGCTACAGTTTATCTGTATATTTATTCATTTAGCTATAGTCCTTGCTGCTGATATCAACGTGCTGTAAACAGTCGGCCAGGCGGGTTGCCAAATCGCAAACTCCTGTTGGGAAGTACGACTTCACCACTCCATATCACTCGTACTTTTTATTATATCTTTATGCG
ATCC 28306	CATTTAGCACAACATACCGAATCCTCCACCACAGCCATCCACCATGAGAGAGGTAGTTAGTTTGAACGGTACGTCGGTCCTTGTTGCTACAGTTTATCTGTATATTTATTCATTTAGCTATAGTCCTTGCTGCTGATATCAACGTGCTGTAAACAGTCGGCCAGGCGGGTTGCCAAATCGCAAACTCCTGTTGGGAAGTACGACTTCACCACTCCATATCACTCGTACTTTTTATTATATCTTTATGCG
ATCC 62541 (601)	CATTTAGCACAACATACCGAATCCTCCACCACAGCCATCCACCATGAGAGAGGTAGTTAGTTTGAACGGTACGTCGGTCCTTGTTGCTACAGTTTATCTGTATATTTATTCATTTAGCTATAGTCCTTGCTGCTGATATCAACGTGCTGTAAACAGTCGGCCAGGCGGGTTGCCAAATCGCAAACTCCTGTTGGGAAGTACGACTTCACCACTCCATATCACTCGTACTTTTTATTATATCTTTATGCG
ATCC 66136 (637)	CATTTAGCACAACATACCGAATCCTCCACCACAGCCATCCACCATGAGAGAGGTAGTTAGTTTGAACGGTACGTCGGTCCTTGTTGCTACAGTTTATCTGTATATTTATTCATTTAGCTATAGTCCTTGCTGCTGATATCAACGTGCTGTAAACAGTCGGCCAGGCGGGTTGCCAAATCGCAAACTCCTGTTGGGAAGTACGACTTCACCACTCCATATCACTCGTACTTTTTATTATATCTTTATGCG
En	CATTTAGCACAACATACCGAATCCTCCACCACAGCCATCCACCATGAGAGAGGTAGTTAGTTTGAACGGTACGTCGGTCCTTGTTGCTACAGTTTATCTGTATATTTATTCATTTAGCTATAGTCCTTGCTGCTGATATCAACGTGCTGTAAACAGTCGGCCAGGCGGGTTGCCAAATCGCAAACTCCTGTTGGGAAGTACGACTTCACCACTCCATATCACTCGTACTTTTTATTATATCTTTATGCG
F270	CATTTAGCACAACATACCGAATCCTCCACCACAGCCATCCACCATGAGAGAGGTAGTTAGTTTGAACGGTACGTCGGTCCTTGTTGCTACAGTTTATCTGTATATTTATTCATTTAGCTATAGTCCTTGCTGCTGATATCAACGTGCTGTAAACAGTCGGCCAGGCGGGTTGCCAAATCGCAAACTCCTGTTGGGAAGTACGACTTCACCACTCCATATCACTCGTACTTTTTATTATATCTTTATGCG
FR00742 2009	CATTTAGCACAACATACCGAATCCTCCACCACAGCCATCCACCATGAGAGAGGTAGTTAGTTTGAACGGTACGTCGGTCCTTGTTGCTACAGTTTATCTGTATATTTATTCATTTAGCTATAGTCCTTGCTGCTGATATCAACGTGCTGTAAACAGTCGGCCAGGCGGGTTGCCAAATCGCAAACTCCTGTTGGGAAGTACGACTTCACCACTCCATATCACTCGTACTTTTTATTATATCTTTATGCG
K940	CATTTAGCACAACATACCGAATCCTCCACCACAGCCATCCACCATGAGAGAGGTAGTTAGTTTGAACGGTACGTCGGTCCTTGTTGCTACAGTTTATCTGTATATTTATTCATTTAGCTATAGTCCTTGCTGCTGATATCAACGTGCTGTAAACAGTCGGCCAGGCGGGTTGCCAAATCGCAAACTCCTGTTGGGAAGTACGACTTCACCACTCCATATCACTCGTACTTTTTATTATATCTTTATGCG
SF06266 2009	CATTTAGCACAACATACCGAATCCTCCACCACAGCCATCCACCATGAGAGAGGTAGTTAGTTTGAACGGTACGTCGGTCCTTGTTGCTACAGTTTATCTGTATATTTATTCATTTAGCTATAGTCCTTGCTGCTGATATCAACGTGCTGTAAACAGTCGGCCAGGCGGGTTGCCAAATCGCAAACTCCTGTTGGGAAGTACGACTTCACCACTCCATATCACTCGTACTTTTTATTATATCTTTATGCG
SF14600 2008	CATTTAGCACAACATACCGAATCCTCCACCACAGCCATCCACCATGAGAGAGGTAGTTAGTTTGAACGGTACGTCGGTCCTTGTTGCTACAGTTTATCTGTATATTTATTCATTTAGCTATAGTCCTTGCTGCTGATATCAACGTGCTGTAAACAGTCGGCCAGGCGGGTTGCCAAATCGCAAACTCCTGTTGGGAAGTACGACTTCACCACTCCATATCACTCGTACTTTTTATTATATCTTTATGCG
UAMH 4042	CATTTAGCACAACATACCGAATCCTCCACCACAGCCATCCACCATGAGAGAGGTAGTTAGTTTGAACGGTACGTCGGTCCTTGTTGCTACAGTTTATCTGTATATTTATTCATTTAGCTATAGTCCTTGCTGCTGATATCAACGTGCTGTAAACAGTCGGCCAGGCGGGTTGCCAAATCGCAAACTCCTGTTGGGAAGTACGACTTCACCACTCCATATCACTCGTACTTTTTATTATATCTTTATGCG
UAMH 5634	CATTTAGCACAACATACCGAATCCTCCACCACAGCCATCCACCATGAGAGAGGTAGTTAGTTTGAACGGTACGTCGGTCCTTGTTGCTACAGTTTATCTGTATATTTATTCATTTAGCTATAGTCCTTGCTGCTGATATCAACGTGCTGTAAACAGTCGGCCAGGCGGGTTGCCAAATCGCAAACTCCTGTTGGGAAGTACGACTTCACCACTCCATATCACTCGTACTTTTTATTATATCTTTATGCG
CH-10	CATTTAGCACAACATACCGAATCCTCCACCATAGCCATCCACCATGAGAGAGGTAGTTAGTTTGAACGGTACGTCGGTCCTTGTTGCTACAGTTTATCTGTATATTTATTCATTTAGCTATAGTCCTTGCTGCTGATATCAACGTGCTGTAAACAGTCGGCCAGGCGGGTTGCCAAATCGCAAACTCCTGTTGGGAAGTACGACTTCACCACTCCATATCACTCGTACTTTTTATTATATCTTTATGCG
Ro	CATTTAGCACAACATACCGAATCCTCCACCATAGCCATCCACCATGAGAGAGGTAGTTAGTTTGAACGGTACGTCGGTCCTTGTTGCTACAGTTTATCTGTATATTTATTCATTTAGCTATAGTCCTTGCTGCTGATATCAACGTGCTGTAAACAGTCGGCCAGGCGGGTTGCCAAATCGCAAACTCCTGTTGGGAAGTACGACTTCACCACTCCATATCACTCGTACTTTTTATTATATCTTTATGCG
TB00011 2006	CATTTAGCACAACATACCGAATCCTCCACCATAGCCATCCACCATGAGAGAGGTAGTTAGTTTGAACGGTACGTCGGTCCTTGTTGCTACAGTTTATCTGTATATTTATTCATTTAGCTATAGTCCTTGCTGCTGATATCAACGTGCTGTAAACAGTCGGCCAGGCGGGTTGCCAAATCGCAAACTCCTGTTGGGAAGTACGACTTCACCACTCCATATCACTCGTACTTTTTATTATATCTTTATGCG
UAMH 5584	CATTTAGCACAACATACCGAATCCTCCACCATAGCCATCCACCATGAGAGAGGTAGTTAGTTTGAACGGTACGTCGGTCCTTGTTGCTACAGTTTATCTGTATATTTATTCATTTAGCTATAGTCCTTGCTGCTGATATCAACGTGCTGTAAACAGTCGGCCAGGCGGGTTGCCAAATCGCAAACTCCTGTTGGGAAGTACGACTTCACCACTCCATATCACTCGTACTTTTTATTATATCTTTATGCG
UAMH 7800	CATTTAGCACAACATACCGAATCCTCCACCATAGCCATCCACCATGAGAGAGGTAGTTAGTTTGAACGGTACGTCGGTCCTTGTTGCTACAGTTTATCTGTATATTTATTCATTTAGCTATAGTCCTTGCTGCTGATATCAACGTGCTGTAAACAGTCGGCCAGGCGGGTTGCCAAATCGCAAACTCCTGTTGGGAAGTACGACTTCACCACTCCATATCACTCGTACTTTTTATTATATCTTTATGCG
396	CATTTAGCACAACATACCGAATCCTCCACCACAGCCATCCACCATGAGAGAGGTAGTTAGTTTGAACGGTACGTCGGTCCTTGTTGCTACAGTTTATCTGTATATTTATTCATTTAGCTATAGTCCTTGCTGCTGATATCAACGTGCTGTAAACAGTCGGCCAGGCGGGTTGCCAAATCGCAAACTCCTGTTGGGAAGTACGACTTCACCACTCCATATCACTCGTACTTTTTATTATATCTTTATGCG
397	CATTTAGCACAACATACCGAATCCTCCACCACAGCCATCCACCATGAGAGAGGTAGTTAGTTTGAACGGTACGTCGGTCCTTGTTGCTACAGTTTATCTGTATATTTATTCATTTAGCTATAGTCCTTGCTGCTGATATCAACGTGCTGTAAACAGTCGGCCAGGCGGGTTGCCAAATCGCAAACTCCTGTTGGGAAGTACGACTTCACCACTCCATATCACTCGTACTTTTTATTATATCTTTATGCG
ATCC MYA-2586(ER-3)	CATTTAGCACAACATACCGAATCCTCCACCACAGCCATCCACCATGAGAGAGGTAGTTAGTTTGAACGGTACGTCGGTCCTTGTTGCTACAGTTTATCTGTATATTTATTCATTTAGCTATAGTCCTTGCTGCTGATATCAACGTGCTGTAAACAGTCGGCCAGGCGGGTTGCCAAATCGCAAACTCCTGTTGGGAAGTACGACTTCACCACTCCATATCACTCGTACTTTTTATTATATCTTTATGCG
SF05792 2009	CATTTAGCACAACATACCGAATCCTCCACCACAGCCATCCACCATGAGAGAGGTAGTTAGTTTGAACGGTACGTCGGTCCTTGTTGCTACAGTTTATCTGTATATTTATTCATTTAGCTATAGTCCTTGCTGCTGATATCAACGTGCTGTAAACAGTCGGCCAGGCGGGTTGCCAAATCGCAAACTCCTGTTGGGAAGTACGACTTCACCACTCCATATCACTCGTACTTTTTATTATATCTTTATGCG
FR00346 2009	CATTTAGCACAACATACCGAATCCTCCACCACAGCCATCCACCATGAGAGAGGTAGTTAGTTTGAACGGTACGTCGGTCCTTGTTGCTACAGTTTATCTGTATATTTATTCATTTAGCTATAGTCCTTGCTGCTGATATCAACGTGCTGTAAACAGTCGGCCAGGCGGGTTGCCAAATCGCAAACTCCTGTTGGGAAGTACGACTTCACCACTCCATATCACTCGTACTTTTTATTATATCTTTATGCG
B3003 (UAMH 12046)	CATTTAGCACAACATACCGAATCCTCCACCACAGCCATCCACCATGAGAGAGGTAGTTAGTTTGAACGGTACGTCGGTCCTTGTTGCTACAGTTTATCTGTATATTTATTCATTTAGCTATAGTCCTTGCTGCTGATATCAACGTGCTGTAAACAGTCGGCCAGGCGGGTTGCCAAATCGCAAACTCCTGTTGGGAAGTACGACTTCACCACTCCATATCACTCGTACTTTTTATTATATCTTTATGCG
TB00024 2005	CATTTAGCACAACATACTGAATCCTCCACCATAGCCATCCACCATGAGAGAGGTAGTTAGTTTGAACGGTACGTCGGTCCTTGTTGCTACAGTTTATCTGTATATTTATTCATTTAGCTATAGTCCTTGCTGCTGATATCAACGTGCTGTAAACAGTCGGCCAGGCGGGTTGCCAAATCGCAAACTCCTGTTGGGAAGTACGACTTCACCACTCCATATCACTCGTACTTTTTATTATATCTTTATGCG
TB00042 2005	CATTTAGCACAACATACTGAATCCTCCACCATAGCCATCCACCATGAGAGAGGTAGTTAGTTTGAACGGTACGTCGGTCCTTGTTGCTACAGTTTATCTGTATATTTATTCATTTAGCTATAGTCCTTGCTGCTGATATCAACGTGCTGTAAACAGTCGGCCAGGCGGGTTGCCAAATCGCAAACTCCTGTTGGGAAGTACGACTTCACCACTCCATATCACTCGTACTTTTTATTATATCTTTATGCG

tub1 (continued)

INTRON — EXON — INTRON — EXON

SF03443 2009	GTCAAATTCACACGACCAATATAAATCGCCAACGATGCTAATGTCTTCAACTGTGTTGCTTTTATAGCTGTATTGCCTGGAACATGGCATTCAGGTAAAACTTATGCCCCTCTTCCTGGCTCGTTGCAGTTTGATGGACTTTTGTTCCCTGACATC* * * * *TACTCGGGATGTGAGACTAATTTGACTCGTCCTCATTTGATAGCCCGATGGATACCTGACTGAAGAAAGAAAAGCTGCCGACCCTGAT
SF05773 2009	GTCAAATTCACACGACCAATATAAATCGCCAACGATGCTAATGTCTTCAACTGTGTTGCTTTTATAGCTGTATTGCCTGGAACATGGCATTCAGGTAAAACTTATGCCCCTCTTCCTGGCTCGTTGCAGTTTGATGGACTTTTGTTCCCTGACATC* * * * *TACTCGGGATGTGAGACTAATTTGACTCGTCCTCATTTGATAGCCCGATGGATACCTGACTGAAGAAAGAAAAGCTGCCGACCCTGAT
B1566 (UAMH 12045)	GTCAAATTCACACGACCAATATAAATCGCCAACGATGCTAATGTCTTCAACTGTGTTGCTTTTATAGCTGTATTGCCTGGAACATGGCATTCAGGTAAAACTTATGCCCCTCTTCCTGGCTCGTTGCAGTTTGATGGACTTTTGTTCCCTGACATC* * * * *TACTCGGGATGTGAGACTAATTTGACTCGTCCTCATTTGATAGCCCGATGGATACCTGACTGAAGAAAGAAAAGCTGCCGACCCTGAT
TB00016 2005	GTCAAATTCACACGACCAATATAAATCGCCAACGATGCTAATGTCTTCAACTGTGTTGCTTTTATAGCTGTATTGCCTGGAACATGGCATTCAGGTAAAACTTATGCCCCTCTTCCTGGCTCGTTGCAGTTTGATGGACTTTTGTTCCCTGACATC* * * * *TACTCGGGATGTGAGACTAATTTGACTCGTCCTCATTTGATAGCCCGATGGATACCTGACTGAAGAAAGAAAAGCTGCCGACCCTGAT
SF06072 2009	GTCAAATTCACACGACCAATATAAATCGCCAACGATGCTAATGTCTTCAACTGTGTTGCTTTTATAGCTGTATTGCCTGGAACATGGCATTCAGGTAAAACTTATGCCCCTCTTCCTGGCTCGTTGCAGTTTGATGGACTTTTGTTCCCTGACATC* * * * *TACTCGGGATGTGAGACTAATTTGACTCGTCCTCATTTGATAGCCCGATGGATACCTGACTGAAGAAAGAAAAGCTGCCGACCCTGAT
SF06354 2009	GTCAAATTCACACGACCAATATAAATCGCCAACGATGCTAATGTCTTCAACTGTGTTGCTTTTATAGCTGTATTGCCTGGAACATGGCATTCAGGTAAAACTTATGCCCCTCTTCCTGGCTCGTTGCAGTTTGATGGACTTTTGTTCCCTGACATC* * * * *TACTCGGGATGTGAGACTAATTTGACTCGTCCTCATTTGATAGCCCGATGGATACCTGACTGAAGAAAGAAAAGCTGCCGACCCTGAT
371	GTCAAATTCACACGACCAATATAAATCGCCAACGATGCTAATGTCTTCAACTGTGTTGCTTTTATAGCTGTATTGCCTGGAACATGGCATTCAGGTAAAACTTATGCCCCTCTTCCTGGCTCGTTGCAGTTTGATGGACTTTTGTTCCCTGACATC* * * * *TACTCGGGATGTGAGACTAATTTGACTCGTCCTCATTTGATAGCCCGATGGATACCTGACTGAAGAAAGAAAAGCTGCCGACCCTGAT
663	GTCAAATTCACACGACCAATATAAATCGCCAACGATGCTAATGTCTTCAACTGTGTTGCTTTTATAGCTGTATTGCCTGGAACATGGCATTCAGGTAAAACTTATGCCCCTCTTCCTGGCTCGTTGCAGTTTGATGGACTTTTGTTCCCTGACATC* * * * *TACTCGGGATGTGAGACTAATTTGACTCGTCCTCATTTGATAGCCCGATGGATACCTGACTGAAGAAAGAAAAGCTGCCGACCCTGAT
664	GTCAAATTCACACGACCAATATAAATCGCCAACGATGCTAATGTCTTCAACTGTGTTGCTTTTATAGCTGTATTGCCTGGAACATGGCATTCAGGTAAAACTTATGCCCCTCTTCCTGGCTCGTTGCAGTTTGATGGACTTTTGTTCCCTGACATC* * * * *TACTCGGGATGTGAGACTAATTTGACTCGTCCTCATTTGATAGCCCGATGGATACCTGACTGAAGAAAGAAAAGCTGCCGACCCTGAT
ATCC 26197 (GA-1)	GTCAAATTCACACGACCAATATAAATCGCCAACGATGCTAATGTCTTCAACTGTGTTGCTTTTATAGCTGTATTGCCTGGAACATGGCATTCAGGTAAAACTTATGCCCCTCTTCCTGGCTCGTTGCAGTTTGATGGACTTTTGTTCCCTGACATC* * * * *TACTCGGGATGTGAGACTAATTTGACTCGTCCTCATTTGATAGCCCGATGGATACCTGACTGAAGAAAGAAAAGCTGCCGACCCTGAT
ATCC 26199 (V)	GTCAAATTCACACGACCAATATAAATCGCCAACGATGCTAATGTCTTCAACTGTGTTGCTTTTATAGCTGTATTGCCTGGAACATGGCATTCAGGTAAAACTTATGCCCCTCTTCCTGGCTCGTTGCAGTTTGATGGACTTTTGTTCCCTGACATC* * * * *TACTCGGGATGTGAGACTAATTTGACTCGTCCTCATTTGATAGCCCGATGGATACCTGACTGAAGAAAGAAAAGCTGCCGACCCTGAT
ATCC 28306	GTCAAATTCACACGACCAATATAAATCGCCAACGATGCTAATGTCTTCAACTGTGTTGCTTTTATAGCTGTATTGCCTGGAACATGGCATTCAGGTAAAACTTATGCCCCTCTTCCTGGCTCGTTGCAGTTTGATGGACTTTTGTTCCCTGACATC* * * * *TACTCGGGATGTGAGACTAATTTGACTCGTCCTCATTTGATAGCCCGATGGATACCTGACTGAAGAAAGAAAAGCTGCCGACCCTGAT
ATCC 62541 (601)	GTCAAATTCACACGACCAATATAAATCGCCAACGATGCTAATGTCTTCAACTGTGTTGCTTTTATAGCTGTATTGCCTGGAACATGGCATTCAGGTAAAACTTATGCCCCTCTTCCTGGCTCGTTGCAGTTTGATGGACTTTTGTTCCCTGACATC* * * * *TACTCGGGATGTGAGACTAATTTGACTCGTCCTCATTTGATAGCCCGATGGATACCTGACTGAAGAAAGAAAAGCTGCCGACCCTGAT
ATCC 66136 (637)	GTCAAATTCACACGACCAATATAAATCGCCAACGATGCTAATGTCTTCAACTGTGTTGCTTTTATAGCTGTATTGCCTGGAACATGGCATTCAGGTAAAACTTATGCCCCTCTTCCTGGCTCGTTGCAGTTTGATGGACTTTTGTTCCCTGACATC* * * * *TACTCGGGATGTGAGACTAATTTGACTCGTCCTCATTTGATAGCCCGATGGATACCTGACTGAAGAAAGAAAAGCTGCCGACCCTGAT
En	GTCAAATTCACACGACCAATATAAATCGCCAACGATGCTAATGTCTTCAACTGTGTTGCTTTTATAGCTGTATTGCCTGGAACATGGCATTCAGGTAAAACTTATGCCCCTCTTCCTGGCTCGTTGCAGTTTGATGGACTTTTGTTCCCTGACATC* * * * *TACTCGGGATGTGAGACTAATTTGACTCGTCCTCATTTGATAGCCCGATGGATACCTGACTGAAGAAAGAAAAGCTGCCGACCCTGAT
F270	GTCAAATTCACACGACCAATATAAATCGCCAACGATGCTAATGTCTTCAACTGTGTTGCTTTTATAGCTGTATTGCCTGGAACATGGCATTCAGGTAAAACTTATGCCCCTCTTCCTGGCTCGTTGCAGTTTGATGGACTTTTGTTCCCTGACATC* * * * *TACTCGGGATGTGAGACTAATTTGACTCGTCCTCATTTGATAGCCCGATGGATACCTGACTGAAGAAAGAAAAGCTGCCGACCCTGAT
FR00742 2009	GTCAAATTCACACGACCAATATAAATCGCCAACGATGCTAATGTCTTCAACTGTGTTGCTTTTATAGCTGTATTGCCTGGAACATGGCATTCAGGTAAAACTTATGCCCCTCTTCCTGGCTCGTTGCAGTTTGATGGACTTTTGTTCCCTGACATC* * * * *TACTCGGGATGTGAGACTAATTTGACTCGTCCTCATTTGATAGCCCGATGGATACCTGACTGAAGAAAGAAAAGCTGCCGACCCTGAT
K940	GTCAAATTCACACGACCAATATAAATCGCCAACGATGCTAATGTCTTCAACTGTGTTGCTTTTATAGCTGTATTGCCTGGAACATGGCATTCAGGTAAAACTTATGCCCCTCTTCCTGGCTCGTTGCAGTTTGATGGACTTTTGTTCCCTGACATC* * * * *TACTCGGGATGTGAGACTAATTTGACTCGTCCTCATTTGATAGCCCGATGGATACCTGACTGAAGAAAGAAAAGCTGCCGACCCTGAT
SF06266 2009	GTCAAATTCACACGACCAATATAAATCGCCAACGATGCTAATGTCTTCAACTGTGTTGCTTTTATAGCTGTATTGCCTGGAACATGGCATTCAGGTAAAACTTATGCCCCTCTTCCTGGCTCGTTGCAGTTTGATGGACTTTTGTTCCCTGACATC* * * * *TACTCGGGATGTGAGACTAATTTGACTCGTCCTCATTTGATAGCCCGATGGATACCTGACTGAAGAAAGAAAAGCTGCCGACCCTGAT
SF14600 2008	GTCAAATTCACACGACCAATATAAATCGCCAACGATGCTAATGTCTTCAACTGTGTTGCTTTTATAGCTGTATTGCCTGGAACATGGCATTCAGGTAAAACTTATGCCCCTCTTCCTGGCTCGTTGCAGTTTGATGGACTTTTGTTCCCTGACATC* * * * *TACTCGGGATGTGAGACTAATTTGACTCGTCCTCATTTGATAGCCCGATGGATACCTGACTGAAGAAAGAAAAGCTGCCGACCCTGAT
UAMH 4042	GTCAAATTCACACGACCAATATAAATCGCCAACGATGCTAATGTCTTCAACTGTGTTGCTTTTATAGCTGTATTGCCTGGAACATGGCATTCAGGTAAAACTTATGCCCCTCTTCCTGGCTCGTTGCAGTTTGATGGACTTTTGTTCCCTGACATC* * * * *TACTCGGGATGTGAGACTAATTTGACTCGTCCTCATTTGATAGCCCGATGGATACCTGACTGAAGAAAGAAAAGCTGCCGACCCTGAT
UAMH 5634	GTCAAATTCACACGACCAATATAAATCGCCAACGATGCTAATGTCTTCAACTGTGTTGCTTTTATAGCTGTATTGCCTGGAACATGGCATTCAGGTAAAACTTATGCCCCTCTTCCTGGCTCGTTGCAGTTTGATGGACTTTTGTTCCCTGACATC* * * * *TACTCGGGATGTGAGACTAATTTGACTCGTCCTCATTTGATAGCCCGATGGATACCTGACTGAAGAAAGAAAAGCTGCCGACCCTGAT
CH-10	GTCAAATTCACACGACCAATATAAATCGCCAACGATGCTAATGTCTTCAACTGTGTTGCTTTTATAGCTGTATTGCCTGGAACATGGCATTCAGGTAAAACTTATGCCCCTCTTCCTGGCTCGTTGCAGTTTGATGGACTTTTGTTCCCTGACATC* * * * *TACTCGGGATGTGAGACTAATTTGACTCGTCCTCATTTGATAGCCCGATGGATACCTGACTGAAGAAAGAAAAGCTGCCGACCCTGAT
Ro	GTCAAATTCACACGACCAATATAAATCGCCAACGATGCTAATGTCTTCAACTGTGTTGCTTTTATAGCTGTATTGCCTGGAACATGGCATTCAGGTAAAACTTATGCCCCTCTTCCTGGCTCGTTGCAGTTTGATGGACTTTTGTTCCCTGACATC* * * * *TACTCGGGATGTGAGACTAATTTGACTCGTCCTCATTTGATAGCCCGATGGATACCTGACTGAAGAAAGAAAAGCTGCCGACCCTGAT
TB00011 2006	GTCAAATTCACACGACCAATATAAATCGCCAACGATGCTAATGTCTTCAACTGTGTTGCTTTTATAGCTGTATTGCCTGGAACATGGCATTCAGGTAAAACTTATGCCCCTCTTCCTGGCTCGTTGCAGTTTGATGGACTTTTGTTCCCTGACATC* * * * *TACTCGGGATGTGAGACTAATTTGACTCGTCCTCATTTGATAGCCCGATGGATACCTGACTGAAGAAAGAAAAGCTGCCGACCCTGAT
UAMH 5584	GTCAAATTCACACGACCAATATAAATCGCCAACGATGCTAATGTCTTCAACTGTGTTGCTTTTATAGCTGTATTGCCTGGAACATGGCATTCAGGTAAAACTTATGCCCCTCTTCCTGGCTCGTTGCAGTTTGATGGACTTTTGTTCCCTGACATC* * * * *TACTCGGGATGTGAGACTAATTTGACTCGTCCTCATTTGATAGCCCGATGGATACCTGACTGAAGAAAGAAAAGCTGCCGACCCTGAT
UAMH 7800	GTCAAATTCACACGACCAATATAAATCGCCAACGATGCTAATGTCTTCAACTGTGTTGCTTTTATAGCTGTATTGCCTGGAACATGGCATTCAGGTAAAACTTATGCCCCTCTTCCTGGCTCGTTGCAGTTTGATGGACTTTTGTTCCCTGACATC* * * * *TACTCGGGATGTGAGACTAATTTGACTCGTCCTCATTTGATAGCCCGATGGATACCTGACTGAAGAAAGAAAAGCTGCCGACCCTGAT
396	GTCAAATTCACACGACCAATATAAATCGCCAACGATGCTAATGTCTTCAACTGTGTTGCTTTTATAGCTGTATTGCCTGGAACATGGCATTCAGGTAAAACTTATTCCCCTCTTCCTGGCTCGTTGCAGTTTGATGGACTTTTGTTCCCTGACATCTTAGA TACTCGGGATGTGAGACTAATTTGACTCGTCCTCATTTGATAGCCCGATGGATACCTGACTGAAGAAAGAAAAGCTGCCGACCCTGAT
397	GTCAAATTCACACGACCAATATAAATCGCCAACGATGCTAATGTCTTCAACTGTGTTGCTTTTATAGCTGTATTGCCTGGAACATGGCATTCAGGTAAAACTTATTCCCCTCTTCCTGGCTCGTTGCAGTTTGATGGACTTTTGTTCCCTGACATCTTAGA TACTCGGGATGTGAGACTAATTTGACTCGTCCTCATTTGATAGCCCGATGGATACCTGACTGAAGAAAGAAAAGCTGCCGACCCTGAT
ATCC MYA-2586(ER-3)	GTCAAATTCACACGACCAATATAAATCGCCAACGATGCTAATGTCTTCAACTGTGTTGCTTTTATAGCTGTATTGCCTGGAACATGGCATTCAGGTAAAACTTATGCCCCTCTTCCTGGCTCGTTGCAGTTTGATGGACTTTTGTTCCCTGACATC* * * * *TACTCGGGATGTGAGACTAATTTGACTCGTCCTCATTTGATAGCCCGATGGATACCTGACTGAAGAAAGAAAAGCTGCCGACCCTGAT
SF05792 2009	GTCAAATTCACACGACCAATATAAATCGCCAACGATGCTAATGTCTTCAACTGTGTTGCTTTTATAGCTGTATTGCCTGGAACATGGCATTCAGGTAAAACTTATGCCCCTCTTCCTGGCTCGTTGCAGTTTGATGGACTTTTGTTCCCTGACATC* * * * *TACTCGGGATGTGAGACTAATTTGACTCGTCCTCATTTGATAGCCCGATGGATACCTTACTGAAGAAAGAAAAGCTGCCGACCCTGAT
FR00346 2009	GTCAAATTCACACGACCAATATAAATGGCCAACGATGCTAATGTCTTCAACTGTGTTGCTTTTATAGCTGTATTGCCTGGAACATGGCATTCAGGTAAAACTTATGCCCCTCTTCCTGGCTCGTTGCAGTTTGATGGACTTTTGTTCCCTGACATC* * * * *TACTCGGGATGTGAGACTAATTTGACTCGTCCTCATTTGATAGCCCGATGGATACCTGACTGAAGAAAGAAAAGCTGCCGACCCTGAT
B3003 (UAMH 12046)	GTCAAATTCACACGACCAATATAAATCGCCAACGATGCTAATGTCTTCAACTGTGTTGCTTTTATAGCTGTATTGCCTGGAACATGGCATTCAGGTAAAACTTATGCCCCTCTTCCTGGCTCGTTGCAGTTTGATGGACTTTTGTTCCCTGACATC* * * * *TACTCGGAATGTGAGACTAATTTGACTCGTCCTCATTTGATAGCCCGATGGATACCTGACTGAAGAAAGAAAAGCTGCCGACCCTGAT
TB00024 2005	GTCAAATTCACACGACCAATATAAATCGCCAACGATGCTAATGTCTTCAACTGTGTTGCTTTTATAGCTGTATTGCCTGGAACATGGCATTCAGGTAAAACTTATGCCCCTCTTCCTGGCTCGTTGCAGTTTGATGGACTTTTGTTCCCTGACATC* * * * *TACTCGGGATGTGAGACTAATTTGACTCGTCCTCATTTGATAGCCCGATGGATACCTGACTGAAGAAAGAAAAGCTGCCGACCCTGAT
TB00042 2005	GTCAAATTCACACGACCAATATAAATCGCCAACGATGCTAATGTCTTCAACTGTGTTGCTTTTATAGCTGTATTGCCTGGAACATGGCATTCAGGTAAAACTTATGCCCCTCTTCCTGGCTCGTTGCAGTTTGATGGACTTTTGTTCCCTGACATC* * * * *TACTCGGGATGTGAGACTAATTTGACTCGTCCTCATTTGATAGCCCGATGGATACCTGACTGAAGAAAGAAAAGCTGCCGACCCTGAT

Figure S1: Nucleotide Sequence Alignments (continued)

tub1 (continued)

EXON — INTRON — EXON

SF03443 2009	CAAGGATTTAATACTTTCTTTTCTGAAACAGGTTCGACAGCCCTCTTCCAACAAAGTGCCGATGGATACATATGGATTTTTGATTTGATTTCGGGAAACTTACATGTACATATCGCTCCCCCTATCTAGGCCAGGGAAAATATGTTCCCCGAACCA
SF05773 2009	CAAGGATTTAATACTTTCTTTTCTGAAACAGGTTCGACAGCCCTCTTCCAACAAAGTGCCGATGGATACATATGGATTTTTGATTTGATTTCGGGAAACTTACATGTACATATCGCTCCCCCTATCTAGGCCAGGGAAAATATGTTCCCCGAACCA
B1566 (UAMH 12045)	CAAGGATTTAATACTTTCTTTTCTGAAACAGGTTCGACAGCCCTCTTCCAACAAAGTGCCGATGGATACATATGGATTTTTGATTTGATTTCGGGAAACTTACATGTACATATCGCGCCCCCTATCTAGGCCAGGGAAAATATGTTCCCCGAACCA
TB00016 2005	CAAGGATTTAATACTTTCTTTTCTGAAACAGGTTCGACAGCCCTCTTCCAACAAAGTGCCGATGGATACATATGGATTTTTGATTTGATTTCGGGAAACTTACATGTACATATCGCGCCCCCTATCTAGGCCAGGGAAAATATGTTCCCCGAACCA
SF06072 2009	CAAGGATTTAATACTTTCTTTTCTGAAACAGGTTCGACAGCCCTCTTCCAACAAAGTGCCGATGGATACATATGGATTTTTGATTTGATTTCGGGAAACTTACATGTACATATCGCCCCCCCGATCTAGGCCAGGGAAAATATGTTCCCCGAACCA
SF06354 2009	CAAGGATTTAATACTTTCTTTTCTGAAACAGGTTCGACAGCCCTCTTCCAACAAAGTGCCGATGGATACATATGGATTTTTGATTTGATTTCGGGAAACTTACATGTACATATCGCCCCCCCGATCTAGGCCAGGGAAAATATGTTCCCCGAACCA
371	CAAGGATTTAATACTTTCTTTTCTGAAACAGGTTCGACAGCCCTCTTCCAACAAAGTGCCGATGGATACATATGGATTTTTGATTTGATTTCGGGAAACTTACATGTACATATCGCCCCCCCTATCTAGGCCAGGGAAAATATGTTCCCCGAACCA
663	CAAGGATTTAATACTTTCTTTTCTGAAACAGGTTCGACAGCCCTCTTCCAACAAAGTGCCGATGGATACATATGGATTTTTGATTTGATTTCGGGAAACTTACATGTACATATCGCCCCCCCTATCTAGGCCAGGGAAAATATGTTCCCCGAACCA
664	CAAGGATTTAATACTTTCTTTTCTGAAACAGGTTCGACAGCCCTCTTCCAACAAAGTGCCGATGGATACATATGGATTTTTGATTTGATTTCGGGAAACTTACATGTACATATCGCCCCCCCTATCTAGGCCAGGGAAAATATGTTCCCCGAACCA
ATCC 26197 (GA-1)	CAAGGATTTAATACTTTCTTTTCTGAAACAGGTTCGACAGCCCTCTTCCAACAAAGTGCCGATGGATACATATGGATTTTTGATTTGATTTCGGGAAACTTACATGTACATATCGCCCCCCCTATCTAGGCCAGGGAAAATATGTTCCCCGAACCA
ATCC 26199 (V)	CAAGGATTTAATACTTTCTTTTCTGAAACAGGTTCGACAGCCCTCTTCCAACAAAGTGCCGATGGATACATATGGATTTTTGATTTGATTTCGGGAAACTTACATGTACATATCGCCCCCCCTATCTAGGCCAGGGAAAATATGTTCCCCGAACCA
ATCC 28306	CAAGGATTTAATACTTTCTTTTCTGAAACAGGTTCGACAGCCCTCTTCCAACAAAGTGCCGATGGATACATATGGATTTTTGATTTGATTTCGGGAAACTTACATGTACATATCGCCCCCCCTATCTAGGCCAGGGAAAATATGTTCCCCGAACCA
ATCC 62541 (601)	CAAGGATTTAATACTTTCTTTTCTGAAACAGGTTCGACAGCCCTCTTCCAACAAAGTGCCGATGGATACATATGGATTTTTGATTTGATTTCGGGAAACTTACATGTACATATCGCCCCCCCTATCTAGGCCAGGGAAAATATGTTCCCCGAACCA
ATCC 66136 (637)	CAAGGATTTAATACTTTCTTTTCTGAAACAGGTTCGACAGCCCTCTTCCAACAAAGTGCCGATGGATACATATGGATTTTTGATTTGATTTCGGGAAACTTACATGTACATATCGCCCCCCCTATCTAGGCCAGGGAAAATATGTTCCCCGAACCA
En	CAAGGATTTAATACTTTCTTTTCTGAAACAGGTTCGACAGCCCTCTTCCAACAAAGTGCCGATGGATACATATGGATTTTTGATTTGATTTCGGGAAACTTACATGTACATATCGCCCCCCCTATCTAGGCCAGGGAAAATATGTTCCCCGAACCA
F270	CAAGGATTTAATACTTTCTTTTCTGAAACAGGTTCGACAGCCCTCTTCCAACAAAGTGCCGATGGATACATATGGATTTTTGATTTGATTTCGGGAAACTTACATGTACATATCGCCCCCCCTATCTAGGCCAGGGAAAATATGTTCCCCGAACCA
FR00742 2009	CAAGGATTTAATACTTTCTTTTCTGAAACAGGTTCGACAGCCCTCTTCCAACAAAGTGCCGATGGATACATATGGATTTTTGATTTGATTTCGGGAAACTTACATGTACATATCGCCCCCCCTATCTAGGCCAGGGAAAATATGTTCCCCGAACCA
K940	CAAGGATTTAATACTTTCTTTTCTGAAACAGGTTCGACAGCCCTCTTCCAACAAAGTGCCGATGGATACATATGGATTTTTGATTTGATTTCGGGAAACTTACATGTACATATCGCCCCCCCTATCTAGGCCAGGGAAAATATGTTCCCCGAACCA
SF06266 2009	CAAGGATTTAATACTTTCTTTTCTGAAACAGGTTCGACAGCCCTCTTCCAACAAAGTGCCGATGGATACATATGGATTTTTGATTTGATTTCGGGAAACTTACATGTACATATCGCCCCCCCTATCTAGGCCAGGGAAAATATGTTCCCCGAACCA
SF14600 2008	CAAGGATTTAATACTTTCTTTTCTGAAACAGGTTCGACAGCCCTCTTCCAACAAAGTGCCGATGGATACATATGGATTTTTGATTTGATTTCGGGAAACTTACATGTACATATCGCCCCCCCTATCTAGGCCAGGGAAAATATGTTCCCCGAACCA
UAMH 4042	CAAGGATTTAATACTTTCTTTTCTGAAACAGGTTCGACAGCCCTCTTCCAACAAAGTGCCGATGGATACATATGGATTTTTGATTTGATTTCGGGAAACTTACATGTACATATCGCCCCCCCTATCTAGGCCAGGGAAAATATGTTCCCCGAACCA
UAMH 5634	CAAGGATTTAATACTTTCTTTTCTGAAACAGGTTCGACAGCCCTCTTCCAACAAAGTGCCGATGGATACATATGGATTTTTGATTTGATTTCGGGAAACTTACATGTACATATCGCCCCCCCTATCTAGGCCAGGGAAAATATGTTCCCCGAACCA
CH-10	CAAGGATTTAATACTTTCTTTTCTGAAACAGGTTCGACAGCCCTCTTCCAACAAAGTGCCGATGGATACATATGGATTTTTGATTTGATTTCGGGAAACTTACATGTACATATCGCCCCCCCTATCTAGGCCAGGGAAAATATGTTCCCCGAACCA
Ro	CAAGGATTTAATACTTTCTTTTCTGAAACAGGTTCGACAGCCCTCTTCCAACAAAGTGCCGATGGATACATATGGATTTTTGATTTGATTTCGGGAAACTTACATGTACATATCGCCCCCCCTATCTAGGCCAGGGAAAATATGTTCCCCGAACCA
TB00011 2006	CAAGGATTTAATACTTTCTTTTCTGAAACAGGTTCGACAGCCCTCTTCCAACAAAGTGCCGATGGATACATATGGATTTTTGATTTGATTTCGGGAAACTTACATGTACATATCGCCCCCCCTATCTAGGCCAGGGAAAATATGTTCCCCGAACCA
UAMH 5584	CAAGGATTTAATACTTTCTTTTCTGAAACAGGTTCGACAGCCCTCTTCCAACAAAGTGCCGATGGATACATATGGATTTTTGATTTGATTTCGGGAAACTTACATGTACATATCGCCCCCCCTATCTAGGCCAGGGAAAATATGTTCCCCGAACCA
UAMH 7800	CAAGGATTTAATACTTTCTTTTCTGAAACAGGTTCGACAGCCCTCTTCCAACAAAGTGCCGATGGATACATATGGATTTTTGATTTGATTTCGGGAAACTTACATGTACATATCGCCCCCCCTATCTAGGCCAGGGAAAATATGTTCCCCGAACCA
396	CAAGGATTTAATACTTTCTTTTCTGAAACAGGTTCGACAGCCCTCTTCCAACAAAGTGCCGATGGATACATATGGATTTTTGATTTGATTTCGGGAAACTTACATGTACATATCGCCCCCCCTATCTAGGCCAGGGAAAATATGTTCCCCGAACCA
397	CAAGGATTTAATACTTTCTTTTCTGAAACAGGTTCGACAGCCCTCTTCCAACAAAGTGCCGATGGATACATATGGATTTTTGATTTGATTTCGGGAAACTTACATGTACATATCGCCCCCCCTATCTAGGCCAGGGAAAATATGTTCCCCGAACCA
ATCC MYA-2586(ER-3)	CAAGGATTTAATACTTTCTTTTCTGAAACAGGTTCGACAGCCCTCTTCCAAGAAAGTGCCGATGGATACATATGGATTTTTGATTTGATTTCGGGAAACTTACATGTACATATCGCCCCCCCTATCTAGGCCAGGGAAAATATGTTCCCCGAACCA
SF05792 2009	CAAGGATTTAATACTTTCTTTTCTGAAACAGGTTCGACAGCCCTCTTCCAACAAAGTGCCGATGGATACATATGGATTTTTGATTTGATTTCGGGAAACTTACATGTACATATCGCCCCCCCTATCTAGGCCAGGGAAAATATGTTCCCCGAACCA
FR00346 2009	CAAGGATTTAATACTTTCTTTTCTGAAACAGGTTCGACAGCCCTCTTCCAACAAAGTGCCGATGGATACATATGGATTTTTGATTTGATTTCGGGAAACTTACATGTACATATCGCCCCCCCGATCTAGGCCAGGGAAAATATGTTCCCCGAACCA
B3003 (UAMH 12046)	CAAGGATTTAATACTTTCTTTTCTGAAACAGGTTCGACAGCCCTCTTCCAACAAAGTGCCGATGGATACATATGGATTTTTGATTTGATTTCGAGAAACTTACATGTACATATCGCCCCCCCTATCTAGGCCAGGGAAAATATGTTCCCCGAACCA
TB00024 2005	CAAGGATTTAATACTTTCTTTTCTGAAACAGGTTCGACAGCCCTCTTCCAAGAAAGTGCCGATGGATACATATGGATTTTTGATTTGATTTCGGGAAACTTACATGTACATATCGCCCCCCCTATCTAGGCCAGGGAAAATATGTTCCCCGAACCA
TB00042 2005	CAAGGATTTAATACTTTCTTTTCTGAAACAGGTTCGACAGCCCTCTTCCAAGAAAGTGCCGATGGATACATATGGATTTTTGATTTGATTTCGGGAAACTTACATGTACATATCGCCCCCCCTATCTAGGCCAGGGAAAATATGTTCCCCGAACCA
