# Supplementary material for: Phylogenetic Analysis Reveals a Cryptic Species Blastomyces gilchristii, sp. nov. within the Human Pathogenic Fungus Blastomyces dermatitidis
Source: PLoS One. 2013 Mar 22;8(3):e59237. doi: 10.1371/journal.pone.0059237 (PMC3606480; doi:10.1371/journal.pone.0059237)
Supplement: Table S1 — Characteristics of B. dermatitidis isolates studied. (PDF) [file pone.0059237.s002.pdf]

Table S1. Characteristics of *Blastomyces spp.* isolates studied

Isolate Identification Number[d]	Geographic Region of Isolation	Analysis Region[e]	Source[a]	Year	PS[b]	ST[c]	Mating Type	Reference
TB00016/2005	Shoal Lake, Ontario, Canada	NOW	Patient (SPT)	2005	Bg	29	HMG	This study
TB00017/2005	Kenora, Ontario, Canada	NOW	Patient (TISS/FLD)	2005	Bg	29	HMG	“ "
TB00018/2005	Kenora, Ontario, Canada	NOW	Patient (SPT)	2005	Bg	29	α Box	“ "
TB00019/2005	Kenora, Ontario, Canada	NOW	Patient (SPT)	2005	Bg	29	HMG	“ "
TB00020/2005	Kenora, Ontario, Canada	NOW	Patient (SPT)	2005	Bg	29	HMG	“ "
TB00021/2005	Kenora, Ontario, Canada	NOW	Patient (SPT)	2005	Bg	29	HMG	“ "
TB00022/2005	Kenora, Ontario, Canada	NOW	Patient (SPT)	2005	Bg	29	α Box	“ "
TB00023/2005	Kenora, Ontario, Canada	NOW	Patient (SPT)	2005	Bg	29	HMG	“ "
TB00024/2005	Kenora, Ontario, Canada	NOW	Patient (SPT)	2005	Bd	4	HMG	“ "
TB00025/2005	Kenora, Ontario, Canada	NOW	Patient (SPT)	2005	Bg	29	α Box	“ "
TB00038/2005	Morson, Ontario, Canada	NOW	Patient (SPT)	2005	Bg	29	HMG	“ "
TB00040/2005	Sioux Lookout, Ontario, Canada	NOW	Patient (SPT)	2005	Bg	30	α Box	“ "
TB00042/2005	Thunder Bay, Ontario, Canada	NOW	Patient (SPT)	2005	Bd	1	HMG	“ "
TB00011/2006	Red Lake, Ontario, Canada	NOW	Patient (SPT)	2006	Bd	7	α Box	“ "
TB00017/2006	Kenora, Ontario, Canada	NOW	Patient (SPT)	2006	Bg	29	α Box	“ "
TB00018/2006	Keewatin, Ontario, Canada	NOW	Patient (SPT)	2006	Bg	29	HMG	“ "
TB00022/2006	Kenora, Ontario, Canada	NOW	Patient (SPT)	2006	Bg	29	HMG	“ "
TB00029/2006	Kenora, Ontario, Canada	NOW	Patient (SPT)	2006	Bg	29	α Box	“ "
TB00032/2006	North Spirit Lake, Ontario, Canada	NOW	Patient (SPT)	2006	Bg	27	HMG	“ "
TB00014/2008	Thunder Bay, Ontario, Canada	NOW	Patient (FLD/TISS)	2008	Bd	1	N/A[f]	“ "
TB00037/2008	Keewatin, Ontario, Canada	NOW	Patient (SPT)	2008	Bg	29	HMG	“ "
FR00742/2009	Mississauga, Ontario, Canada	O	Patient (BAL)	2009	Bg	32	HMG	“ "
FR00059/2009	Little Current, Ontario, Canada	O	Patient (SPT)	2009	Bg	31	HMG	“ "
FR00404/2009	Birch Island, Ontario, Canada	O	Patient (SPT)	2009	Bd	11	N/A[f]	“ "
FR00346/2009	Ottawa, Ontario, Canada	O	Patient (TISS)	2009	Bd	5	N/A[f]	“ "
SF03443/2009	Ottawa, Ontario, Canada	O	Patient (BAL)	2009	Bd	20	α Box	“ "
SF14600/2008	Markham, Ontario, Canada	O	Patient (BW)	2008	Bd	19	HMG	“ "
SF05773/2009	McKerrow, Ontario, Canada	O	Patient (SPT)	2009	Bd	11	HMG	“ "
SF05792/2009	Newmarket, Ontario, Canada	O	Patient (BW)	2009	Bd	6	HMG	“ "
SF06266/2009	Mississauga, Ontario, Canada	O	Patient (BAL)	2009	Bd	18	HMG	“ "
SF06072/2009	Parry Sound, Ontario, Canada	O	Patient (SPT)	2009	Bd	9	HMG	“ "
SF06354/2009	St. George, Ontario, Canada	O	Patient (ASP)	2009	Bd	10	HMG	“ "

Table S1. Characteristics of *Blastomyces spp.* isolates studied *(Continued)*

Isolate Identification Number[d]	Geographic Region of Isolation	Analysis Region[e]	Source[a]	Year	PS[b]	ST[c]	Mating Type	Reference
ATCC 66136 (637)	Sarnia, Ontario, Canada	O	Environment (soil)	1986	Bg	31	α Box	[1]
UAMH 4042	Regina, Saskatchewan, Canada	AS	Patient (TISS/FLD)	1976	Bd	16	N/A[f]	This study
UAMH 5634	Regina, Saskatchewan, Canada	AS	Patient (BW)		Bd	17	HMG	" "
UAMH 5635	Regina, Saskatchewan, Canada	AS	Patient (SPT)		Bd	17	HMG	" "
UAMH 5584	Edmonton, Alberta, Canada	AS	Canine (BLD)	1986	Bd	28	HMG	" "
UAMH 7800	Calgary, Alberta, Canada	AS	Patient	1994	Bd	8	HMG	" "
CDC B1566 (UAMH 12045)	South Africa	A	Patient		Bg	27	α Box	[2]
B3003 (UAMH 12046)	Rwanda	A	Patient		Bd	3	HMG	[3]
CDC B1562 (UAMH 12051)	Zimbabwe	A	Patient		Bg	29	α Box	[2]
ATCC 62541 (601)	Eagle River, Wisconsin, USA	WM	Patient	1985	Bg	33	α Box	[4]
ATCC 28306	Wisconsin, USA	WM	Patient		Bd	36	HMG	[5]
ATCC MYA-2585 (ERC-2)	Eagle River, Wisconsin, USA	WM	Canine (feces)	1996	Bg	33	HMG	[6]
ATCC MYA-2586 (ER-3)[g]	Eagle River, Wisconsin, USA	WM	Environment (soil/woodpile)	1997	Bd	26	HMG	[7]
F252	Wisconsin, USA	WM	Patient		Bg	29	α Box	[8]
ATCC 62583 (599)	Eagle River, Wisconsin, USA	WM	Patient	1985	Bg	33	HMG	[9]
ATCC 60636	Eagle River, Wisconsin, USA	WM	Environment (soil)	1984	Bg	33	HMG	[4]
ATCC 60637	Tomorrow River, Wisconsin, USA	WM	Environment (soil)	1985	Bg	33	HMG	[9]
600	Eagle River, Wisconsin, USA	WM	Patient		Bg	33	HMG	[8]
588	Eagle River, Wisconsin, USA	WM	Patient		Bg	33	HMG	[8]
591	Eagle River, Wisconsin, USA	WM	Patient		Bg	33	HMG	[8]
590	Eagle River, Wisconsin, USA	WM	Patient		Bg	33	HMG	[8]
594	Eagle River, Wisconsin, USA	WM	Patient		Bg	33	α Box	[8]
241	Eagle River, Wisconsin, USA	WM	Environment		Bg	33	HMG	[8]
641	Oconto Falls, Wisconsin, USA	WM	Patient		Bg	33	HMG	[8]
642	Oconto Falls, Wisconsin, USA	WM	Patient		Bg	33	HMG	[8]
Kr	Tomorrow River, Wisconsin, USA	WM	Patient		Bg	33	α Box	[8]
SLH-14081[g]	Wisconsin, USA	WM	Patient		Bg	33	HMG	Broad
F192	Minnesota, USA	WM	Patient		Bg	30	HMG	[8]
F270	Minnesota, USA	WM	Patient		Bd	22	HMG	[8]
ATCC 26199 (V)[g]	South Carolina, USA	SEC	Patient	1970	Bd	24	HMG	[10]
371	South Carolina, USA	SEC	Patient		Bd	21	α Box	[8]
664	Traveler's Rest, South Carolina, USA	SEC	Patient		Bd	25	α Box	[8]

Table S1. Characteristics of *Blastomyces spp.* isolates studied *(Continued)*

Isolate Identification Number[d]	Geographic Region of Isolation	Analysis Region[e]	Source[a]	Year	PS[b]	ST[c]	Mating Type	Reference
663	Traveler's Rest, South Carolina, USA	SEC	Patient		Bd	34	α Box	[8]
Gu	Chicago, Illinois, USA	SEC	Patient		Bd	24	HMG	[8]
En	Chicago, Illinois, USA	SEC	Patient		Bd	35	HMG	[8]
K940	Kentucky, USA	SEC	Patient		Bd	23	HMG	[8]
K966	Kentucky, USA	SEC	Patient		Bd	23	HMG	[8]
394	Georgia, USA	SEC	Environment		Bd	36	HMG	[8]
395	Georgia, USA	SEC	Environment		Bd	36	HMG	[8]
396	Augusta, Georgia, USA	SEC	Environment	1963	Bd	12	HMG	[11]
397	Georgia, USA	SEC	Environment		Bd	13	HMG	[8]
ATCC 26197 (GA-1)	Georgia, USA	SEC	Patient (TISS)		Bd	15	α Box	[12]
CH-6	Mississippi, USA	SEC	Patient		Bd	14	α Box	This study
CH-7	Mississippi, USA	SEC	Patient		Bd	14	α Box	“ "
CH-10	Mississippi, USA	SEC	Patient		Bd	14	α Box	[8]
Ro	Louisiana, USA	SEC	Patient		Bd	2	α Box	[8]

[a] Clinical specimen types were abbreviated as follows: sputum (SPT), tissue (TISS), fluid (FLD), bronchoalveolar lavage (BAL), bronchial washings (BW), aspirate (ASP), and blood (BLD).
[b] Phylogenetic species (PS) were abbreviated as *Blastomyces dermatitidis* (Bd) or *Blastomyces gilchristii* (Bg).
[c] Multilocus Sequence Typing (MLST) Sequence Type (ST).
[d] Alternate identification numbers used in the literature are provided in brackets.
[e] Isolates were divided into analysis regions as described in the Materials and Methods. Analysis regions are abbreviated as follows: northwestern Ontario (NWO); central and southern Ontario (O); Alberta, Saskatchewan (AS); Wisconsin, Minnesota (WM); southern Africa (South Africa, Rwanda, and Zimbabwe) (A); southeastern and central United States (South Carolina, Georgia, Louisiana, Mississippi, Kentucky, and Illinois) (SEC).
[f] Mating type was not amplified, as the isolate could not be cultured during the manuscript revision period.
[g] Complete genome sequence available from the *Blastomyces dermatitidis* Sequencing Project, Broad Institute of Harvard and MIT (http://www.broadinstitute.org/).

REFERENCES

1. Bakerspigel A, Kane J, Schaus D (1986) Isolation of *Blastomyces dermatitidis* from an earthen floor in southwestern Ontario, Canada. J Clin Microbiol 24: 890-891.

2. Sudman MS, Kaplan W (1974) Antigenic relationship between American and African isolates of *Blastomyces dermatitidis* as determined by immunofluorescence. Appl Microbiol 27: 496-499.

3. Kaufman L, Standard PG, Weeks RJ, Padhye AA (1983) Detection of two *Blastomyces dermatitidis* serotypes by exoantigen analysis. J Clin Microbiol 18: 110-114.

4. Klein BS, Vergeront JM, Weeks RJ, Kumar UN, Mathai GM, et al. (1986) Isolation of *Blastomyces dermatitidis* in soil associated with a large outbreak of blastomycosis in Wisconsin. N Engl J Med 314: 529-534.

5. Kaplan W, Kaufman L (1963) Specific fluorescent antiglobulins for the detection and identification of *Blastomyces dermatitidis* yeast-phase cells. Mycopathol Mycol Appl 19: 173-180.

6. Baumgardner DJ, Paretsky DP (1997) Identification of *Blastomyces dermatitidis* in the stool of a dog with acute pulmonary blastomycosis. J Med Vet Mycol 35: 419-421.

7. Baumgardner DJ, Paretsky DP (1999) The in vitro isolation of *Blastomyces dermatitidis* from a woodpile in north central Wisconsin, USA. Med Mycol 37: 163-168.

8. McCullough MJ, DiSalvo AF, Clemons KV, Park P, Stevens DA (2000) Molecular epidemiology of *Blastomyces dermatitidis*. Clin Infect Dis 30: 328-335.

9. Klein BS, Vergeront JM, DiSalvo AF (1987) Two outbreaks of blastomycosis along rivers in Wisconsin. Am Rev Respir Dis 136: 1333-1338.

10. Harvey RP, Schmid ES, Carrington CC, Stevens DA (1978) Mouse model of pulmonary blastomycosis: Utility, simplicity, and quantitative parameters. Am Rev Respir Dis 117: 695-703.

11. Denton JF, DiSalvo AF (1964) Isolation of *Blastomyces dermatitidis* from natural sites at Augusta, Georgia. Am J Trop Med Hyg 13: 716-722.

12. Cox RA, Best GK (1972) Cell wall composition of two strains of *Blastomyces dermatitidis* exhibiting differences in virulence for mice. Infect Immun 5: 449-453.
