# Supplementary material for: Correction: Phylogenetic Analysis Reveals a Cryptic Species Blastomyces gilchristii, sp. nov. within the Human Pathogenic Fungus Blastomyces dermatitidis
Source: PLoS One. 2016 Dec 9;11(12):e0168018. doi: 10.1371/journal.pone.0168018 (PMC5148079; doi:10.1371/journal.pone.0168018)
Supplement: S1 Table — (DOCX) [file pone.0168018.s001.docx]

# Table S1. Characteristics of *Blastomyces spp.* isolates studied

| **Isolate Identification**  **Number^a^** | **Analysis**  **Geographic Region of Isolation _b_**  **Region** | | **Source^c^** | **Year** | **PS^d^** | **ST^e^** | **Mating Type** | **Reference** |
| --- | --- | --- | --- | --- | --- | --- | --- | --- |
| TB00016/2005 | Shoal Lake, Ontario, Canada | NOW | Patient (SPT) | 2005 | Bg | 29 | HMG | This study |
| TB00017/2005 | Kenora, Ontario, Canada | NOW | Patient (TISS/FLD) | 2005 | Bg | 29 | HMG | “ " |
| TB00018/2005 | Kenora, Ontario, Canada | NOW | Patient (SPT) | 2005 | Bg | 29 | α Box | “ " |
| TB00019/2005 | Kenora, Ontario, Canada | NOW | Patient (SPT) | 2005 | Bg | 29 | HMG | “ " |
| TB00020/2005 | Kenora, Ontario, Canada | NOW | Patient (SPT) | 2005 | Bg | 29 | HMG | “ " |
| TB00021/2005 | Kenora, Ontario, Canada | NOW | Patient (SPT) | 2005 | Bg | 29 | HMG | “ " |
| TB00022/2005 | Kenora, Ontario, Canada | NOW | Patient (SPT) | 2005 | Bg | 29 | α Box | “ " |
| TB00023/2005 | Kenora, Ontario, Canada | NOW | Patient (SPT) | 2005 | Bg | 29 | HMG | “ " |
| TB00024/2005 | Kenora, Ontario, Canada | NOW | Patient (SPT) | 2005 | Bd | 4 | HMG | “ " |
| TB00025/2005 | Kenora, Ontario, Canada | NOW | Patient (SPT) | 2005 | Bg | 29 | α Box | “ " |
| TB00038/2005 | Morson, Ontario, Canada | NOW | Patient (SPT) | 2005 | Bg | 29 | HMG | “ " |
| TB00040/2005 | Sioux Lookout, Ontario, Canada | NOW | Patient (SPT) | 2005 | Bg | 30 | α Box | “ " |
| TB00042/2005 | Thunder Bay, Ontario, Canada | NOW | Patient (SPT) | 2005 | Bd | 1 | HMG | “ " |
| TB00011/2006 | Red Lake, Ontario, Canada | NOW | Patient (SPT) | 2006 | Bd | 7 | α Box | “ " |
| TB00017/2006 | Kenora, Ontario, Canada | NOW | Patient (SPT) | 2006 | Bg | 29 | α Box | “ " |
| TB00018/2006 | Keewatin, Ontario, Canada | NOW | Patient (SPT) | 2006 | Bg | 29 | HMG | “ " |
| TB00022/2006 | Kenora, Ontario, Canada | NOW | Patient (SPT) | 2006 | Bg | 29 | HMG | “ " |
| TB00029/2006 | Kenora, Ontario, Canada | NOW | Patient (SPT) | 2006 | Bg | 29 | α Box | “ " |
| TB00032/2006 | North Spirit Lake, Ontario, Canada | NOW | Patient (SPT) | 2006 | Bg | 27 | HMG | “ " |
| TB00014/2008 | Thunder Bay, Ontario, Canada | NOW | Patient (FLD/TISS) | 2008 | Bd | 1 | N/A^f^ | “ " |
| TB00037/2008 | Keewatin, Ontario, Canada | NOW | Patient (SPT) | 2008 | Bg | 29 | HMG | “ " |
| FR00742/2009 | Mississauga, Ontario, Canada | O | Patient (BAL) | 2009 | Bg | 32 | HMG | “ " |
| FR00059/2009 | Little Current, Ontario, Canada | O | Patient (SPT) | 2009 | Bg | 31 | HMG | “ " |
| FR00404/2009 | Birch Island, Ontario, Canada | O | Patient (SPT) | 2009 | Bd | 11 | N/A^f^ | “ " |
| FR00346/2009 | Ottawa, Ontario, Canada | O | Patient (TISS) | 2009 | Bd | 5 | N/A^f^ | “ " |
| SF03443/2009 | Ottawa, Ontario, Canada | O | Patient (BAL) | 2009 | Bd | 20 | α Box | “ " |
| SF14600/2008 | Markham, Ontario, Canada | O | Patient (BW) | 2008 | Bd | 19 | HMG | “ " |
| SF05773/2009 | McKerrow, Ontario, Canada | O | Patient (SPT) | 2009 | Bd | 11 | HMG | “ " |
| SF05792/2009 | Newmarket, Ontario, Canada | O | Patient (BW) | 2009 | Bd | 6 | HMG | “ " |
| SF06266/2009 | Mississauga, Ontario, Canada | O | Patient (BAL) | 2009 | Bd | 18 | HMG | “ " |
| SF06072/2009 | Parry Sound, Ontario, Canada | O | Patient (SPT) | 2009 | Bd | 9 | HMG | “ " |
| SF06354/2009 | St. George, Ontario, Canada | O | Patient (ASP) | 2009 | Bd | 10 | HMG | “ " |

# Table S1. Characteristics of *Blastomyces spp.* isolates studied *(Continued)*

| **Isolate Identification**  **Number^a^** | **Analysis**  **Geographic Region of Isolation _b_**  **Region** | | **Source^c^** | **Year** | **PS^d^** | **ST^e^** | **Mating Type** | **Reference** |
| --- | --- | --- | --- | --- | --- | --- | --- | --- |
| ATCC 66136 (637) | Sarnia, Ontario, Canada | O | Environment (soil) | 1986 | Bg | 31 | α Box | [81] |
| UAMH 4042 | Regina, Saskatchewan, Canada | AS | Patient (TISS/FLD) | 1976 | Bd | 16 | N/A^f^ | This study |
| UAMH 5634 | Regina, Saskatchewan, Canada | AS | Patient (BW) |  | Bd | 17 | HMG | “ " |
| UAMH 5635 | Regina, Saskatchewan, Canada | AS | Patient (SPT) |  | Bd | 17 | HMG | “ " |
| UAMH 5584 | Edmonton, Alberta, Canada | AS | Canine (BLD) | 1986 | Bd | 28 | HMG | “ " |
| UAMH 7800 | Calgary, Alberta, Canada | AS | Patient | 1994 | Bd | 8 | HMG | “ " |
| CDC B1566 (UAMH 10245) | South Africa | A | Patient |  | Bg | 27 | α Box | [74] |
| CDC B3003 (UAMH 10246) | Rwanda | A | Patient |  | Bd | 3 | HMG | [82] |
| CDC B1562 (UAMH 10251) | Zimbabwe | A | Patient |  | Bg | 29 | α Box | [74] |
| ATCC 62541 (601) | Eagle River, Wisconsin, USA | WM | Patient | 1985 | Bg | 33 | α Box | [60] |
| ATCC 28306 | Wisconsin, USA | WM | Patient |  | Bd | 36 | HMG | [83] |
| ATCC MYA-2585 (ERC-2) | Eagle River, Wisconsin, USA | WM | Canine (feces) | 1996 | Bg | 33 | HMG | [84] |
| ATCC MYA-2586 (ER-3)^g^ | Eagle River, Wisconsin, USA | WM | Environment (soil/woodpile) | 1997 | Bd | 26 | HMG | [63] |
| F252 | Wisconsin, USA | WM | Patient |  | Bg | 29 | α Box | [33] |
| ATCC 62583 (599) | Eagle River, Wisconsin, USA | WM | Patient | 1985 | Bg | 33 | HMG | [70] |
| ATCC 60636 | Eagle River, Wisconsin, USA | WM | Environment (soil) | 1984 | Bg | 33 | HMG | [60] |
| ATCC 60637 | Tomorrow River, Wisconsin, USA | WM | Environment (soil) | 1985 | Bg | 33 | HMG | [70] |
| 600 | Eagle River, Wisconsin, USA | WM | Patient |  | Bg | 33 | HMG | [33] |
| 588 | Eagle River, Wisconsin, USA | WM | Patient |  | Bg | 33 | HMG | [33] |
| 591 | Eagle River, Wisconsin, USA | WM | Patient |  | Bg | 33 | HMG | [33] |
| 590 | Eagle River, Wisconsin, USA | WM | Patient |  | Bg | 33 | HMG | [33] |
| 594 | Eagle River, Wisconsin, USA | WM | Patient |  | Bg | 33 | α Box | [33] |
| 241 | Eagle River, Wisconsin, USA | WM | Environment |  | Bg | 33 | HMG | [33] |
| 641 | Oconto Falls, Wisconsin, USA | WM | Patient |  | Bg | 33 | HMG | [33] |
| 642 | Oconto Falls, Wisconsin, USA | WM | Patient |  | Bg | 33 | HMG | [33] |
| Kr | Tomorrow River, Wisconsin, USA | WM | Patient |  | Bg | 33 | α Box | [33] |
| SLH-14081^g^ | Wisconsin, USA | WM | Patient |  | Bg | 33 | HMG | Broad |
| F192 | Minnesota, USA | WM | Patient |  | Bg | 30 | HMG | [33] |
| F270 | Minnesota, USA | WM | Patient |  | Bd | 22 | HMG | [33] |
| ATCC 26199 (V)^g^ | South Carolina, USA | SEC | Patient | 1970 | Bd | 24 | HMG | [85] |
| 371 | South Carolina, USA | SEC | Patient |  | Bd | 21 | α Box | [33] |
| 664 | Traveler's Rest, South Carolina,  USA | SEC | Patient |  | Bd | 25 | α Box | [33] |

# Table S1. Characteristics of *Blastomyces spp.* isolates studied *(Continued)*

| **Isolate Identification**  **Number^a^** | **Analysis Geographic Region of Isolation _b_**  **Region** | | **Source^c^** | **Year** | **PS^d^** | **ST^e^** | **Mating Type** | **Reference** |
| --- | --- | --- | --- | --- | --- | --- | --- | --- |
| 663 | Traveler's Rest, South Carolina,  USA | SEC | Patient |  | Bd | 34 | α Box | [33] |
| Gu | Chicago, Illinois, USA | SEC | Patient |  | Bd | 24 | HMG | [33] |
| En | Chicago, Illinois, USA | SEC | Patient |  | Bd | 35 | HMG | [33] |
| K940 | Kentucky, USA | SEC | Patient |  | Bd | 23 | HMG | [33] |
| K966 | Kentucky, USA | SEC | Patient |  | Bd | 23 | HMG | [33] |
| 394 | Georgia, USA | SEC | Environment |  | Bd | 36 | HMG | [33] |
| 395 | Georgia, USA | SEC | Environment |  | Bd | 36 | HMG | [33] |
| 396 | Augusta, Georgia, USA | SEC | Environment | 1963 | Bd | 12 | HMG | [86] |
| 397 | Georgia, USA | SEC | Environment |  | Bd | 13 | HMG | [33] |
| ATCC 26197 (GA-1) | Georgia, USA | SEC | Patient (TISS) |  | Bd | 15 | α Box | [87] |
| CH-6 | Mississippi, USA | SEC | Patient |  | Bd | 14 | α Box | This study |
| CH-7 | Mississippi, USA | SEC | Patient |  | Bd | 14 | α Box | “ " |
| CH-10 | Mississippi, USA | SEC | Patient |  | Bd | 14 | α Box | [33] |
| Ro | Louisiana, USA | SEC | Patient |  | Bd | 2 | α Box | [33] |

a

Alternate identification numbers used in the literature are provided in brackets.

b

Isolates were divided into analysis regions as described in the Materials and Methods. Analysis regions are abbreviated as follows: northwestern

Ontario (NWO); central and southern Ontario (O); Alberta, Saskatchewan (AS); Wisconsin, Minnesota (WM); southern Africa (South Africa, Rwanda, and Zimbabwe) (A); southeastern and central United States (South Carolina, Georgia, Louisiana, Mississippi, Kentucky, and Illinois) (SEC).

c

Clinical specimen types were abbreviated as follows: sputum (SPT), tissue (TISS), fluid (FLD), bronchoalveolar lavage (BAL), bronchial washings (BW), aspirate (ASP), and blood (BLD).

d

Phylogenetic species (PS) were abbreviated as *Blastomyces dermatitidis* (Bd) or *Blastomyces gilchristii* (Bg).

e

Multilocus Sequence Typing (MLST) Sequence Type (ST).

f

Mating type was not amplified, as the isolate could not be cultured during the manuscript revision period.

g

Complete genome sequence available from the *Blastomyces dermatitidis* Sequencing Project, Broad Institute of Harvard and MIT (http://www.broadinstitute.org/).
